# Printable Conductive Hydrogels for Electrochemical Biosensing and Soft Bioelectronic Interfaces

**DOI:** 10.1002/advs.202521216

**Published:** 2026-04-23

**Authors:** Lukas Hein, Renan Colucci, Xin Wei, Tsvetomir Ivanov, Katharina Landfester, Ulrike Kraft, Maria Villiou

**Affiliations:** ^1^ Max Planck Institute for Polymer Research (MPIP) Mainz Germany

**Keywords:** 3d printing, conductive hydrogels, conductive polymers, gate electrodes, glucose sensors, organic electrochemical transistors (OECTs), soft bioelectronics

## Abstract

The development of flexible, conductive biomaterials is key to advancing next‐generation biosensors and wearable health monitoring systems. However, combining printability, mechanical tunability, biocompatibility, and electronic performance within a single hydrogel remains a significant challenge. Here, we present a facile method to fabricate biofunctional conducting poly(ethylene glycol)–poly(pyrrole) (PEG–PPy) hydrogels via 3D‐printing. The soft and flexible PEG–PPy hydrogels feature tunable mechanical properties and can be easily loaded with bioreceptors, enabling integration into sensing platforms. The composite consists of a poly(ethylene glycol) diacrylate matrix and a conductive polypyrrole filler. By optimizing photopolymerization conditions, we enable extrusion printing of complex, multi‐layered structures with excellent shape fidelity (printability ≈ 1). The resulting hydrogels exhibit tunable stiffness (15–120 kPa), high cytocompatibility (>90%), and robust mechanical integrity. Integration of the hydrogel as a gate electrode in an organic electrochemical transistor yielded transconductance values comparable to conventional Ag/AgCl gates, confirming its electrochemical performance. Furthermore, embedding glucose oxidase into the hydrogel enabled enzymatic glucose sensing over a physiologically relevant range (1–100 mm). This cost‐effective, multifunctional, and versatile PEG–PPy hydrogel platform offers a scalable route toward soft, flexible, printable electronic interfaces.

## Graphical abstract

Flexible, conductive hydrogels that integrate printability, mechanical tunability, biocompatibility, and electronic performance remain challenging to achieve. Here, we develop 3D‐printable poly(ethylene glycol)–poly(pyrrole)‐ hydrogels with tissue‐like mechanics, high cytocompatibility, and robust electrochemical function. Integrated into organic electrochemical transistors, they enable enzymatic glucose sensing, providing a scalable platform for bioelectronic interfaces, wearable diagnostics, and soft sensing technologies.

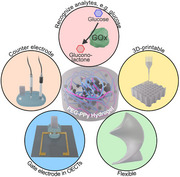

## 1 Introduction

The development of flexible and conductive materials has accelerated significantly in recent years, driven by the demand for next‐generation bioelectronic platforms capable of interfacing seamlessly with soft biological tissues [1, 2]. Among these, hydrogels have proven to be a particularly thrilling material as they contain a high water content, are naturally biocompatible, and are mechanically tunable – properties that make them capable of closely mimicking native body tissue environments [3, 4]. Their ionically permeable and mechanically flexible 3D polymer networks not only facilitate encapsulation of biologically active species such as antibodies and enzymes, but are also capable of behaving as universal scaffolds for drug delivery, tissue regeneration, and biosensing [5, 6, 7].

Polymers like Poly(ethylene glycol) (PEG), poly(N‐isopropylacrylamide), poly(acrylamide), alginate, or gelatin are often utilized to fabricate hydrogels. PEG is widely recognized as a “gold‐standard” biomaterial due to its excellent biocompatibility, bioinertness, and intrinsically low‐fouling characteristics [8, 9]. However, despite their favorable mechanical and biological properties, these hydrogels are inherently electrically insulating and therefore cannot support electronic conduction on their own [1, 10].

Incorporating conjugated polymers such as poly(3,4‐ethylenedioxythiophene): polystyrene sulfonate (PEDOT:PSS) [11], c(PPy) [12], and poly(aniline) [13] into hydrogel networks enables the resulting composites to support mixed ionic/electronic conduction. Among these, PPy and PEDOT:PSS are particularly well suited for bioelectronics due to their favorable biocompatibility profiles [14]. Such conductive hydrogels have therefore found use in electrochemical biosensing [15], neural interfacing [16], and organic electrochemical transistors (OECTs) [17].

Although integrating one or two conjugated polymers into hydrogel matrices allows for the production of bioelectronic components with high conductivity, biocompatibility, and stability, achieving these properties in soft materials remains a significant challenge. Conjugated polymers generally possess limited flexibility due to their intrinsic morphological characteristics, and although the named examples above are considered biocompatible, elevated concentrations of the conjugated polymers can induce cellular toxicity [18, 19]. Importantly, these responses are often attributed not to the polymer backbones themselves, but to residual dopants, additives, or specific morphological features. For example, polystyrene sulfonate (PSS), the common dopant in PEDOT:PSS, may induce cytotoxicity when present in excess or when leached from the material into the surrounding environment [20, 21].

This creates a trade‐off between conductivity, biocompatibility, and mechanical properties, i.e., flexibility or stiffness [22, 23]. In order to preserve conductivity, the use of additives like ionic liquids [24, 25], dimethyl sulfoxide (DMSO) [26, 27], and ethylene glycol [28] has been adopted in the past. However, these additives often compromise biocompatibility and/or result in a Young's modulus greater than 100 kPa, which cannot properly mimic the mechanical properties of skin, intestines, or other soft tissues.

An alternative approach to introducing conductive additives that may compromise biocompatibility is the direct integration of soft hydrogels into organic electrochemical transistor (OECT) architectures. When the hydrogel serves as an active OECT component rather than merely a passive supporting electrode [29], the device can maintain full biocompatibility while accommodating a moderate reduction in electronic conductivity. This is enabled by the intrinsic volumetric signal amplification of OECTs [30], which permits efficient ion–electron coupling even in soft, hydrated materials.

These hybrid systems based on a conducting hydrogel integrated into the OECTs architecture [31] have gained significant attention, owing to their self‐amplification power, high transconductance, low‐voltage operation, and compatibility with aqueous environments [28, 32, 33] For instance, Dai et al. [27] developed a poly(3,3′‐bis[2‐[2‐(2‐methoxyethoxy)ethoxy]ethoxy]2,2′:5′,2′′‐terthiophene)‐based OECTs with tissue‐mimetic mechanical properties and enhanced electrical properties. Bai et al. [34] introduced a hydrogel‐encapsulated OECT platform for continuous glucose monitoring. Lee et al. [35] constructed an ultrathin poly(vinyl alcohol)‐based OECT for signal acquisition and intimate contact with tissue. While these studies underscore the potential of employing soft, conducting hydrogels to replace rigid conventional semiconductors in OECTs, they primarily focused on the channel. However, given their unique combination of ionic conductivity, softness, and biocompatibility, hydrogels also show strong potential to serve as gate electrodes. An additional advantage of employing soft biofunctional hydrogels as gate electrodes is that bioreceptors can be immobilized directly within the hydrated gate matrix, rather than on the channel itself, thereby preventing unintended alterations to the channel's ionic and electronic transport characteristics [36, 37, 38]. However, the transition from stiff films to fully hydrated conducting hydrogels as gate electrodes necessitates challenging prerequisites such as electrochemical stability, high signal‐to‐noise ratio, and efficient charge injection.

**FIGURE 1:**
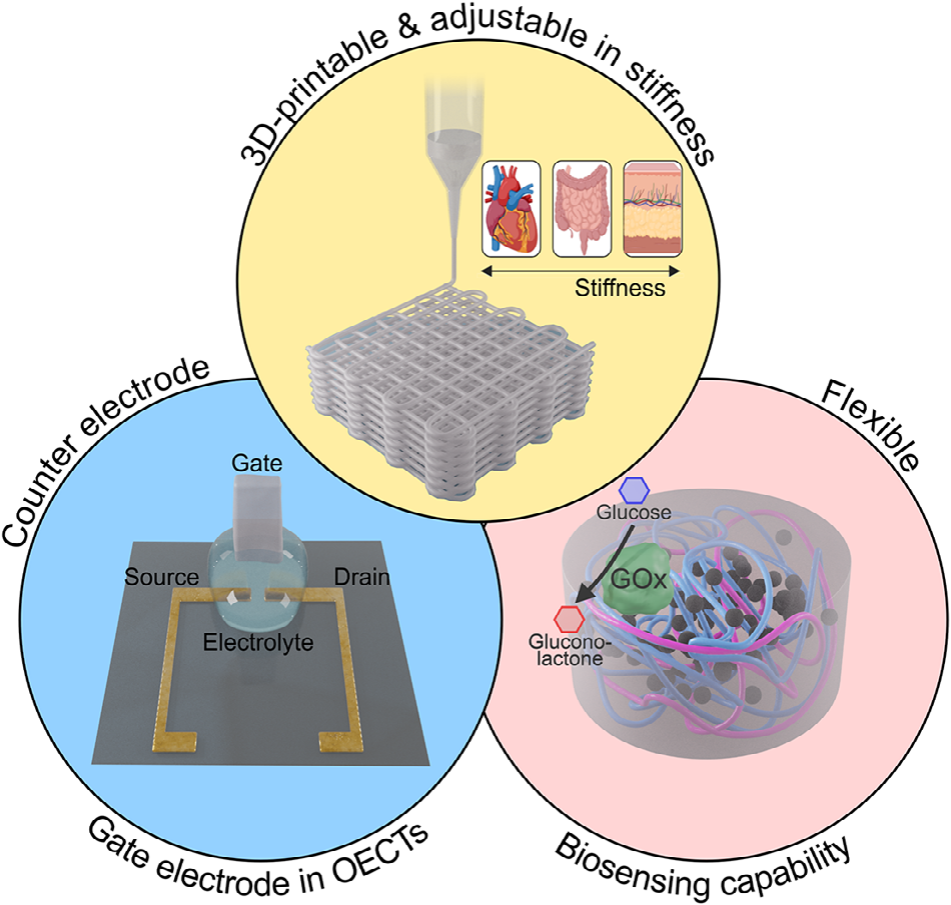
Overview: Conductive, extrusion‐based 3D‐printable PEG‐PPy hydrogels with tunable stiffness, allowing the mechanical properties to be tailored to mimic different tissues, including heart, intestine, and skin. The PEG‐PPy network functions as a soft gate electrode in organic electrochemical transistors (OECTs) and can immobilize enzymes such as GOx, enabling glucose sensing applications. Created with BioRender. Hein, L. (2025) https://BioRender.com/l5c9ck7.

In this study, we present a robust strategy for fabricating cost‐effective, 3D‐printable, flexible, conducting poly(ethylene glycol)–poly(pyrrole) (PEG–PPy) hydrogels via photoinitiated polymerization. By incorporating Pluronic F127 as a sacrificial templating agent and by optimizing formulation and crosslinking conditions, we achieve multi‐layered hydrogel architectures with excellent print fidelity and tunable mechanical properties tailored to match a wide range of soft biological tissues (kPa range). Importantly, we demonstrate that these hydrogels can function effectively as gate electrodes in OECTs, achieving transconductance and stability comparable to those of standard Ag/AgCl electrodes. We further validate their biofunctionality by enzymatically functionalizing the hydrogel with glucose oxidase (GOx), enabling selective biosensing across physiologically relevant glucose concentrations. By combining ionic and electronic conductivity with high biocompatibility and mechanical softness, this material platform addresses a critical need in the field of bioelectronic applications, paving the way for soft, conformable, and implantable bioelectronic devices. Beyond glucose sensing, the hydrogel's modularity and printability position it as a versatile platform for future multimodal biosensors targeting diverse biomarkers at the interface of soft tissue (Figure 1).

## 2 Results and Discussion

### 2.1 Engineering PEG‐PPy Inks for Developing Printable Hydrogels

**FIGURE 2:**
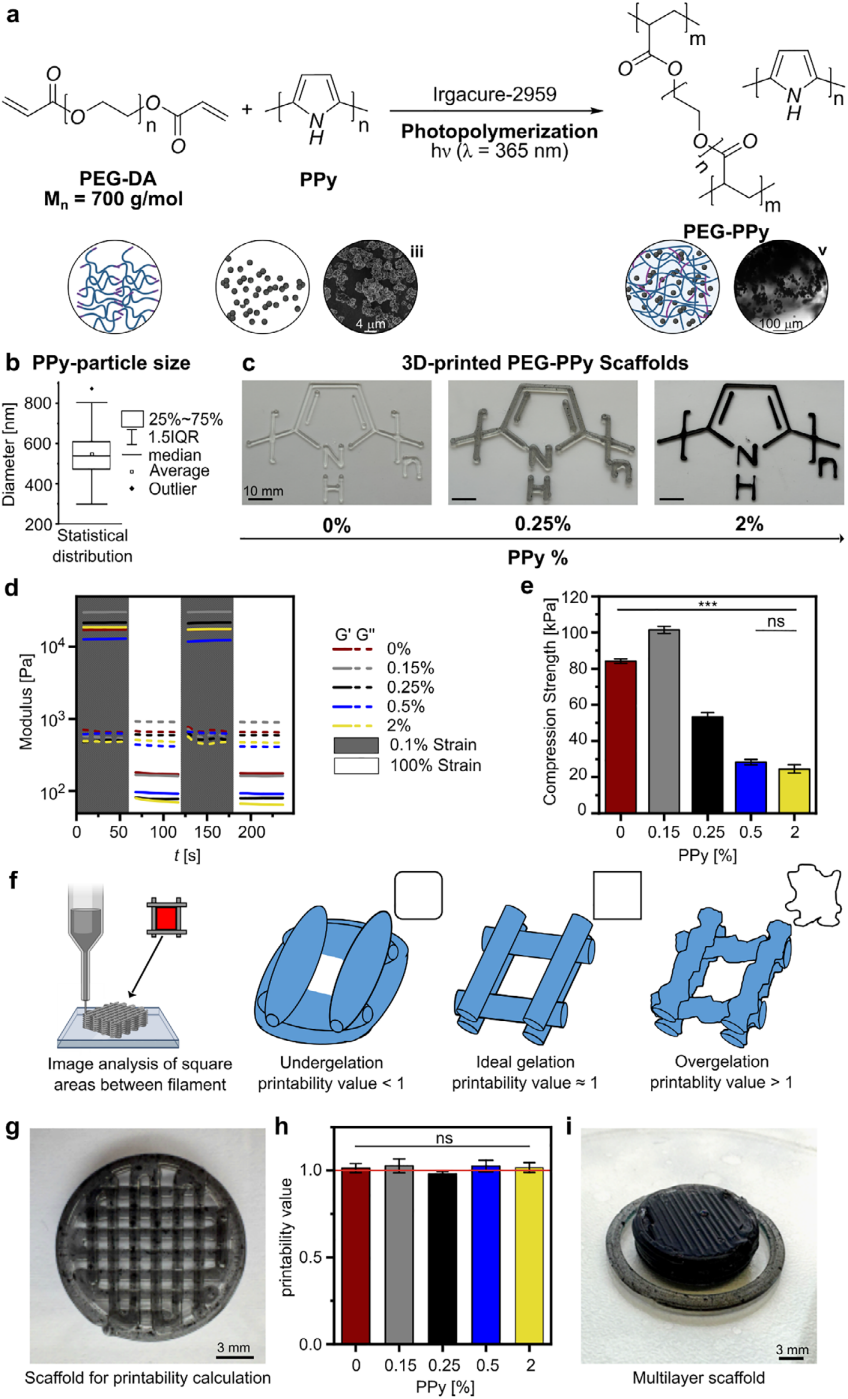
(a) Synthesis of PEG‐PPy conductive hydrogels by photopolymerization using Irgacure 2959. PPy and the composite material were imaged by scanning electron microscopy (SEM) (iii, Figure S3) and optical microscopy (v, Figure S5), respectively. (b) Particle size distribution of PPy calculated from SEM. (c) Extrusion‐based 3D printing (Stainless steel 18 Ga / 38 mm diameter nozzle, 37°C, printing speed 6 mm s^−1^, post‐curing with equal conditions as for hydrogels) of PEG‐PPy composites using 20% PEG‐DA, 2% v/v Irgacure‐2959, and 40% Pluronic F127 as a sacrificial template for temporary support. These composites contain increasing amounts of conductive polymer (PPy). (i–iii) Scale Bars: 10 mm. (d) Dynamic time sweeps of PEG‐PPy inks varying between 0.1% strain and 100% strain. (e) Compression test after UV‐crosslinking scaffolds with Pluronic F127. Number of samples: 3 (*n* = 3). Data is presented as the mean ± the standard deviation and analyzed by variance analysis (ANOVA) with ns *p* > 0.05, ^***^ *p* < 0.001. (f) Schematic illustration of printability assessment. Square areas between filaments are imaged and analyzed with the ideal gelation, achieving a printability value Pr of 1. (g) Lattice structured scaffolds for printability determination. Scale Bar: 3 mm. (h) Printability of PEG‐PPy composites calculated from (g) Number of samples: 5 (*n* = 5). Data is presented as the mean ± the standard deviation and analyzed by variance analysis (ANOVA) with ns *p* > 0.05. (i) Printing of multilayer models (9‐layer). Scale Bar: 3 mm. Created with BioRender; Hein, L. (2025) https://BioRender.com/hb8bzi.

PPy was synthesized following the method described by Seike et al. [39], with minor modifications. The product was analyzed by solid‐state hydrogen magic angle spinning nuclear magnetic resonance spectroscopy (^1^H‐MAS‐NMR), scanning electron microscopy (SEM) and Fourier transformed infrared spectroscopy (FT‐IR) (Figures S1–S3). SEM revealed spherical particles with an average diameter of 550 nm (Figure 2a,b). Furthermore, we observed clusters/agglomerations in the µm‐range as was also reported by Seike et al. [39].

PEG‐PPy composite hydrogels were developed by adding the conductive polymer (PPy) in poly(ethylene glycol)‐diacrylate (PEG‐DA, *M_n_*  =  700 g mol^−1^) solution, with 2‐hydroxy‐4′‐(2‐hydroxyethoxy)‐2‐methylpropiophenon (Irgacure 2959) serving as photoinitiator. The mixture was sonicated, stirred at ambient temperature, and then instantly photopolymerized under UV‐irradiation to obtain a poly(ethylene glycol)‐based hydrogel‐matrix embedded with conductive PPy (Figure 2a; Figures S4 and S5).

To investigate the effect of PPy incorporation on the hydrogels properties, we tested multiple concentrations of PEG‐DA (10, 15, 20 wt.%) and PPy (0–4 wt.%) at similar conditions (2% v/v photoinitiator, *λ* = 365 nm, *I* = 135 mW cm^−2^, irradiation dose = 16.2 J cm^−2^) (Table 1). Stable composite hydrogels were successfully synthesized at PPy concentrations of up to 4%. Beyond this concentration, gelation did not occur within the applied UV dose illustrating the limitations of photopolymerization (Table 1). The yielded hydrogels exhibit inherent flexibility, which can be tuned by the concentration of PEG‐DA (Figure S6).

**TABLE 1:** Synthesis of PEG‐PPy conductive hydrogels with various amounts of PEG‐DA (10–20 wt.%) and PPy (0% – 4%).+ illustrates successful gelation/synthesis. – displays no gelation/limitation of photopolymerization.

PPy [%]	0	0.25	0.5	0.75	1.0	1.5	2.0	2.5	3.0	3.5	4.0
PEG‐DA (10%)	+	+	+	+	+	+	+	+	−	−	−
PEG‐DA (15%)	+	+	+	+	+	+	+	+	+	+	−
PEG‐DA (20%)	+	+	+	+	+	+	+	+	+	+	+

The observed limitations in gelation can be attributed to the influence of PPy on the composite hydrogel system. During photopolymerization, PEG‐DA undergoes crosslinking through radical polymerization of its acrylate groups, forming a 3D hydrogel network. However, as the PPy concentration increases, this crosslinking process becomes progressively hindered. PPy absorbs and scatters UV light, lowering the efficiency of photoinitation within the hydrogel matrix. Additionally, the PPy particles act as physical barriers, restricting the mobility and interaction of PEG‐DA monomers. As a result, higher PPy concentrations lead to a decreased crosslinking density, ultimately preventing complete gelation within the applied irradiation time (120 s). This cross‐linking reduction also manifests as a visibly softer PEG‐PPy hydrogel, highlighting a direct correlation between PPy loading and hydrogel stiffness.

To further advance the PEG‐PPy system toward additive manufacturing, we optimized the formulation for extrusion‐based 3D printing. Pluronic F‐127 was incorporated into the precursor solution to modulate shear‐thinning behavior and viscosity, facilitating smooth and continuous filament extrusion for formulations containing up to 2 wt.% PPy (Figure 2c; Figure S7).

Rheological evaluation confirms that the inks exhibit solid‐like behavior at low oscillatory strain (G′ > G″), while transitioning into a flowable sol state under high strain (Figure 2d). This reversible gel–sol–gel transition supports shape retention after extrusion and indicates that supramolecular interactions primarily govern structural recovery. The influence of PPy content on rheology was further investigated (Figure S8): the storage modulus (G′) initially increases from ∼17 to ∼30 kPa with 0.15 wt.% PPy addition, suggesting reinforcement of the hydrogel network. However, at 0.5 wt.% PPy, G′ decreases to ∼12 kPa, before rising again to ∼18 kPa at 2 wt.% PPy, highlighting a non‐linear contribution of PPy to network mechanical integrity.

Mechanical properties of the printed and photocrosslinked hydrogels were examined under compression (Figure 2e). The formulation containing 0.15 wt.% PPy exhibited the highest compressive strength (101.44 ± 1.97 kPa), consistent with the rheological reinforcement observed at this concentration. At higher PPy loadings, the compressive strength decreased markedly (e.g., 24.53 ± 2.29 kPa at 2 wt.% PPy), likely due to reduced UV penetration and attenuated crosslinking efficiency resulting from increased light absorption by the PPy phase.

To quantitatively assess printability and shape fidelity, model infill structures were analyzed using the method described by Ouyang et al. [40]. Briefly, the printed lattice structures were analyzed by examining a cylinder, which consist of a two layer grid structure, and the printability value *Pr* of the enclosed area is defined by Equation (1), with *L* being the perimeter and *A* the enclosed area [40]:

(1) $$Pr=\frac{L^{2}}{16A}$$

A printability value close to 1 indicates ideal printing fidelity (Figure 2f). When analyzing the lattice scaffold (Figure 2g) of all formulations up to 2% PPy, excellent printability was observed, as depicted in Figure 2h, with printability values of 1.01 ± 0.03, 1.03 ± 0.04, 0.98 ± 0.01, and 1.03 ± 0.03 for 0%, 0.15%, 0.25%, and 0.5% PPy, respectively. Notably, at 2% of PPy a value of 1.02 ± 0.03 was maintained, demonstrating the versatility of the ink formulation even at higher conductive polymer content.

Utilizing the excellent printability of the material, we fabricated multi‐layered constructs and successfully produced scaffolds consisting of up to nine layers while maintaining high shape fidelity and structural precision (Figure 2i).

Overall, these findings highlight the promise of PEG‐PPy composites for additive manufacturing, providing a foundation for the development of conductive, mechanically tunable 3D‐printed sensors.

### 2.2 Multifaceted Characterization of PEG‐PPy Hydrogels: From Material Properties to Biocompatibility

**FIGURE 3:**
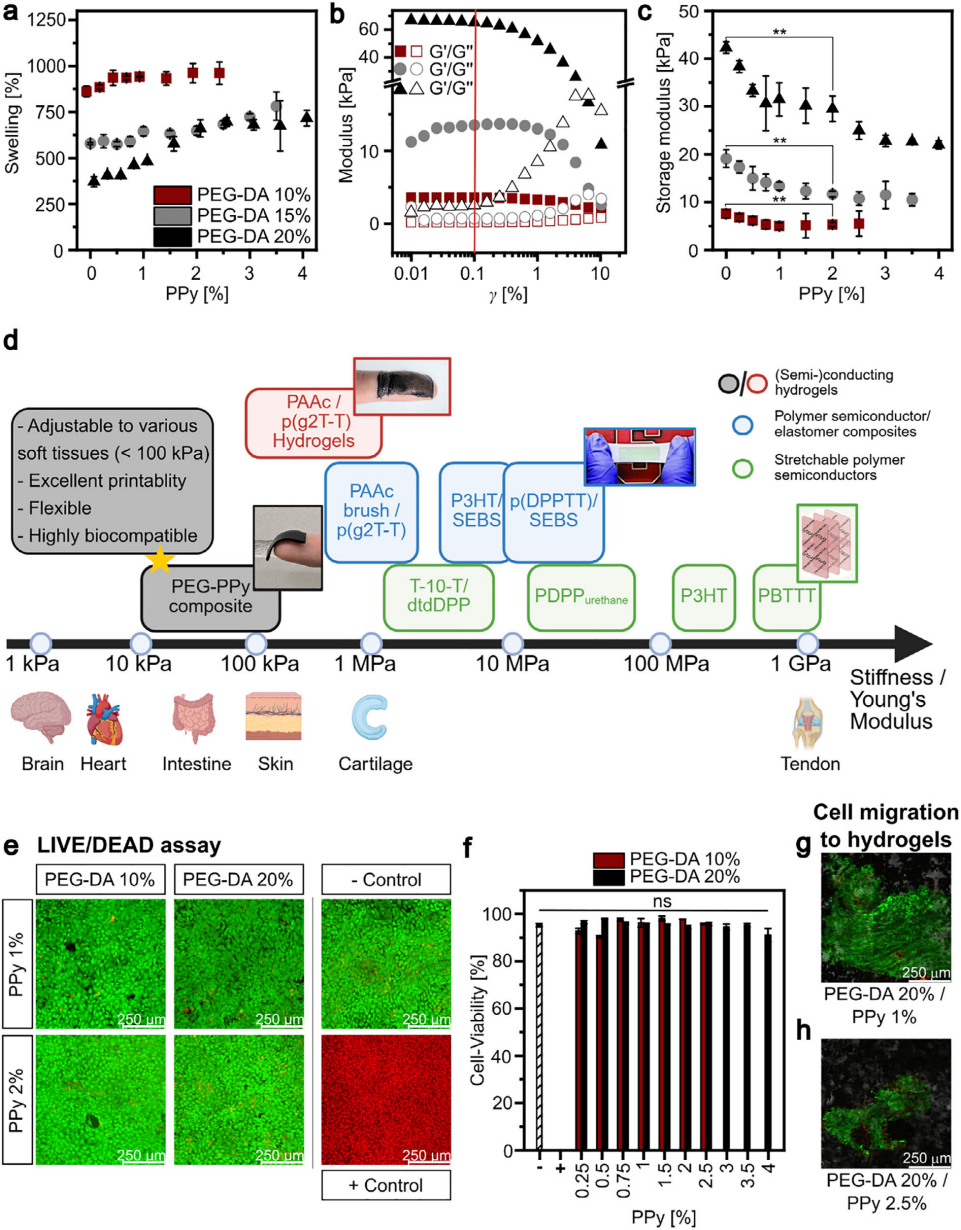
Characterization of PEG‐PPy hydrogels. (a) Swelling (*Q*) behavior, (b) Dynamic strain sweeps (DSS) and (c) Storage modulus (G') of PEG‐PPy hydrogels obtained from plate‐plate rheology (8 mm plates with solvent trap, *T*  =  25°C, 0.1% oscillation strain, 10 rad s^−1^ oscillation frequency, constant axial force of 0.3 N). Number of samples: 3 (*n* = 3). Data is presented as the mean ± the standard deviation and analyzed by variance analysis (ANOVA) with ns *p* > 0.05, ^*^ *p* < 0.05, ^**^ *p* < 0.01 ^***^ *p* < 0.001. (d) Calculated Young's modulus / stiffness range of hydrogel composites (*E*  =  16, 35, or 90 kPa for 2% PPy and 10%, 15% or 20% PEG‐DA respectively) in comparison to published work [27, bib46–51]. Reproduced from Dai et al., *Science*, https://doi.org/10.1126/science.adp9314 2024, AAAS [27]. Reproduced from Xu et al., *Science*, https://doi.org/10.1126/science.aah4496 2017, AAAS [48]. Reprinted from B. O'Connor et al. *ACS nano* 2010, *4*, 7538–7544. Copyright 2010 American Chemical Society [51]. Biocompatibility studies of PEG‐PPy hydrogels after 20 h of cell culture: (e) Fluorescence images of NIH‐3T3 (ACC 59) cells. Viable cells are stained with calcein‐AM (green), while dead cells are stained with propidium iodide (red). (f) Cell viability after exposure to PEG‐PPy hydrogels, compared to untreated control groups. Number of technical replicates: 2 (*n* = 2). Data is presented as the mean ± the standard deviation and analyzed by variance analysis (ANOVA) with ns *p* > 0.05. (g,h) Cell migration to a PEG‐PPy hydrogels after LIVE/DEAD assay. Living cells are observed by fluorescence images. Created with BioRender. Hein, L. (2025) https://BioRender.com/jz3ne1d.

To gain a more comprehensive understanding of the behavior and performance of the PEG‐PPy composite hydrogels developed in Section 2.1, several characterization methods were employed. Swelling behavior was assessed by immersing the hydrogels in ultrapure water, with weight measurements taken after reaching swelling equilibrium (72 h) and after subsequent drying (at 50°C for 72 h). The effects of varying PEG‐DA concentrations (10%–20%) and different amounts of PPy (0%–4%) on swelling were examined. As illustrated in Figure 3a, an increase in PEG‐DA concentration resulted in a decrease in swelling, which can be attributed to a denser crosslinked network. Specifically, the PEG‐DA 10% hydrogel exhibited a swelling ratio of 862% ± 29%, while the PEG‐DA 20% hydrogel swelled to only 371% ± 28%. The swelling behavior of the hydrogels was further influenced by the incorporation of PPy, with all hydrogels exhibiting a near‐linear increase in swelling as the PPy concentration increased (Figure 3a). For instance, the 15% PEG‐DA hydrogel swelled to 578% ± 29%, while the inclusion of 3% PPy increased the swelling to 962% ± 52%. A similar trend was observed at higher PEG‐DA concentrations, with a more pronounced swelling increase. Specifically, the 20% PEG‐DA gel swelled to 371% ± 28%, while the 2% PEG‐PPy composite swelled to a significantly higher value (661% ± 46%). These results suggest that the incorporation of PPy reduces the crosslinking density of the hydrogels, likely due to PPy's absorption of UV light, which impedes photoinitiation and consequently decreases crosslinking efficiency.

Furthermore, the gel content (Figure S9) was evaluated to determine the extent of polymer incorporation into the hydrogel network, which plays a crucial role in the material's mechanical, structural, and electrical properties. The gel content was assessed by immersing the hydrogels in ultrapure water for 48 h at ambient temperature to remove residual unreacted polymer, followed by drying at 50°C for 72 h and weighing the dried gel. The mass of the gel was then compared to the initial mass of the hydrogel precursor. For all three PEG‐DA concentrations (10%, 15% and 20%), gel content exceeded 95% in the absence of PPy. However, with increasing amounts of PPy, a decrease in gel content was observed. These findings align with the observed swelling behavior and suggest that the reduction in gel content is primarily attributed to the impaired photoinitiation process and the decreased crosslinking density caused by the increased presence of PPy in the polymeric network.

The mechanical properties of the PEG‐PPy composite gels were further investigated to gain a deeper understanding of how these changes in structure influenced their performance. The mechanical properties of the composite PEG‐PPy gels were characterized by dynamic frequency sweeps (DFS), dynamic strain sweeps (DSS), and dynamic time sweeps (DTS) with a rheometer equipped with a plate‐plate geometry and a solvent trap at 25°C and 40°C. DFS and DSS were first performed to determine the linear viscoelastic regime (LVR) of the hydrogels, which was found between 2.5 and 15 rad s^−1^ frequency and strain values of 0.01%–0.2% (Figure 3b; Figure S10). Subsequently, DTS measurements were carried out at 0.1% strain and 5 rad s^−1^ frequency to obtain the storage modulus (G’) and loss modulus (G′) of the composite gels, at 25°C. All measured gels exhibited a higher storage modulus (G′  =  3 – 49 kPa) than loss modulus (G′  =  0.1 – 3 kPa), confirming that the photopolymerization process successfully induced crosslinking, and the gels displayed solid‐like behavior.

As the PEG‐DA concentration increased from 10% to 15% and 20%, the G″ increased from 8 to 20 and 42 kPa, respectively (Figure 3c). In contrast, an exponential decrease in G′ was observed with increasing PPy concentration. At a concentration of 2% PPy, the storage modulus G′ of all three utilized PEG‐DA concentrations decreased from 8, 20, and 42 to 5, 12, and 30 kPa, respectively. These findings are consistent with the compressive and rheological measurements of the printable gels (Figure 2d; Figure S8) and also with the swelling and gel content results (Figure 3a; Figure S9). Together, these results support the conclusion that the incorporation of PPy impairs the photopolymerization process, leading to a reduction in crosslinking efficiency and resulting in altered mechanical properties. For the loss modulus G″, we found increasing values with higher crosslinking density, but no trend within the individual PEG‐DA concentrations (Figure S11).

To further quantify the stiffness of the hydrogels, we calculated the Young's modulus (*E*) using the storage modulus (G′) and Poisson's ratio (∼0.5 for hydrogels) as shown in Equation (2) [41]:

(2)

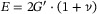




The calculated *E* ranged from 24 to 15 kPa (0%−2.5% PPy) for the 10% PEG‐DA gels, 60 to 40 kPa (0%−3.5% PPy) for the 15% PEG‐DA gels, and 127 to 66 kPa (0%−4% PPy) for the 20% PEG‐DA gels (Figure 3c). These values are comparable to the mechanical properties of various soft tissues in the human body (Figure 3d), such as skin (∼100 kPa), smooth muscle, breast tissue, and intestinal tissue (10 – 80 kPa) [42]. Hence, by adjusting the crosslinker concentration and PPy content, we can target different tissue types.

Such fine‐tuning of hydrogel stiffness can be achieved through various strategies. Adjusting the composition of the hydrogel, such as modifying the concentration of PEG‐DA and the amount of PPy, directly influences the mechanical properties by altering the crosslinking density and mesh size. For example, increasing the PEG‐DA concentration typically enhances crosslinking, resulting in a stiffer material (Figure 3c). Similarly, varying the PPy content can influence the photopolymerization process, as higher concentrations of PPy can reduce the crosslinking density due to its UV absorption properties, leading to a softer hydrogel (Figure 3a–c). Additionally, adjusting the molecular weight (MW) of PEG in the formulation can further impact the network's flexibility and mechanical strength, with higher molecular weights generally enhancing the hydrogel's elasticity and mechanical stability [43]. The photoinitiator type and concentration, along with the irradiation dose during the photopolymerization process, also play a crucial role in determining the extent of crosslinking and, consequently, the hydrogel's mechanical properties [44]. By carefully controlling these factors − composition, PPy concentration, PEG molecular weight, photoinitiator type and concentration, and irradiation parameters – hydrogels can be engineered with tailored mechanical properties to meet the specific requirements of diverse biomedical applications.

We also investigated the degradability of the composite hydrogels and the potential influence of the PPy in this process (Figures S12–S14). The degradability study was conducted by developing PEG‐DA hydrogels containing 10%, 15%, or 20% PEG‐DA with or without 2% PPy – the latter concentration selected based on an optimal balance of mechanical properties and conductivity, as discussed in Section 2.1. The hydrogels were then immersed in phosphate‐buffered saline (PBS) and incubated at 37 °C to simulate physiological conditions. The weight loss of the hydrogels was monitored by weighing in regular intervals over a period of 70 days, with the results normalized to the initial weight (day 0). Our findings revealed that significant degradation occurred only in the 10% PEG‐DA hydrogels, where swelling was also more pronounced. In contrast, no notable degradation was observed at higher crosslinker concentrations (Figures S12–S14). For the 10% PEG‐DA hydrogel, an exponential decrease in weight, with approximately 30% of the hydrogel mass degraded after 70 days. Notably, the composite hydrogel containing 2% PPy exhibited a slower degradation profile compared to the unmodified PEG‐DA hydrogel.

Furthermore, a biocompatibility study was conducted to evaluate the toxicity of the PEG‐PPy composite hydrogels. NIH‐3T3 murine fibroblast cells were seeded on a polystyrene plate and exposed to the hydrogels in a 2D cell culture system. After 24 h of contact, the hydrogels were removed, and the cells were stained with Calcein‐AM (green, for live cells) and propidium iodide (red, for dead cells Figure 3e). As shown in Figure 3f, over 90% cell viability was observed, and fibroblasts were seen migrating toward the hydrogels (Figure 3g,h). Cell viability was calculated by Equation (3) [45]:

(3) $$\begin{array}{ccc} & & Cell\;Viability\;\%\;\\ & & =\frac{total\;n^{\circ }\;of\;cells-n^{\circ }\;of\;stained\;cells}{total\;n^{\circ }\;of\;cells}\cdot 100\% \end{array}$$

No significant differences in cell viability were observed between the different PEG‐DA and PPy concentrations. These results confirm that the composite hydrogels are non‐cytotoxic. This, combined with their tunable mechanical properties and degradation rates, underscores their potential for safe use in in situ biosensing.

### 2.3 Unveiling the Electrical and Electrochemical Properties of PEG‐PPy Hydrogels

The composite PEG‐PPy hydrogels were further characterized for their electrochemical properties, which are critical for their prospective application in biosensing. PPy is well‐regarded for its excellent electrical conductivity, making it a promising candidate for biosensing platforms [52]. Additionally, literature reports highlight PPy's notable environmental stability and its ability to promote cell adhesion and proliferation across various cell types [53], findings that align with our experimental results (Section 2.2). Importantly, PPy exhibits enhanced integration with biological tissues, rendering it particularly suitable for applications that require both reliable electrical performance and sustained biocompatibility [54, 55, 56, 57]. Building on these advantageous properties, a thorough electrical and electrochemical evaluation were conducted to characterize the composite hydrogels. To quantitatively assess the electrical and electrochemical characteristics, the hydrogels underwent a series of measurements, including resistance testing, current‐voltage (*I*–*V*) profiling, cyclic voltammetry (CV), and electrochemical impedance spectroscopy (EIS).

**FIGURE 4:**
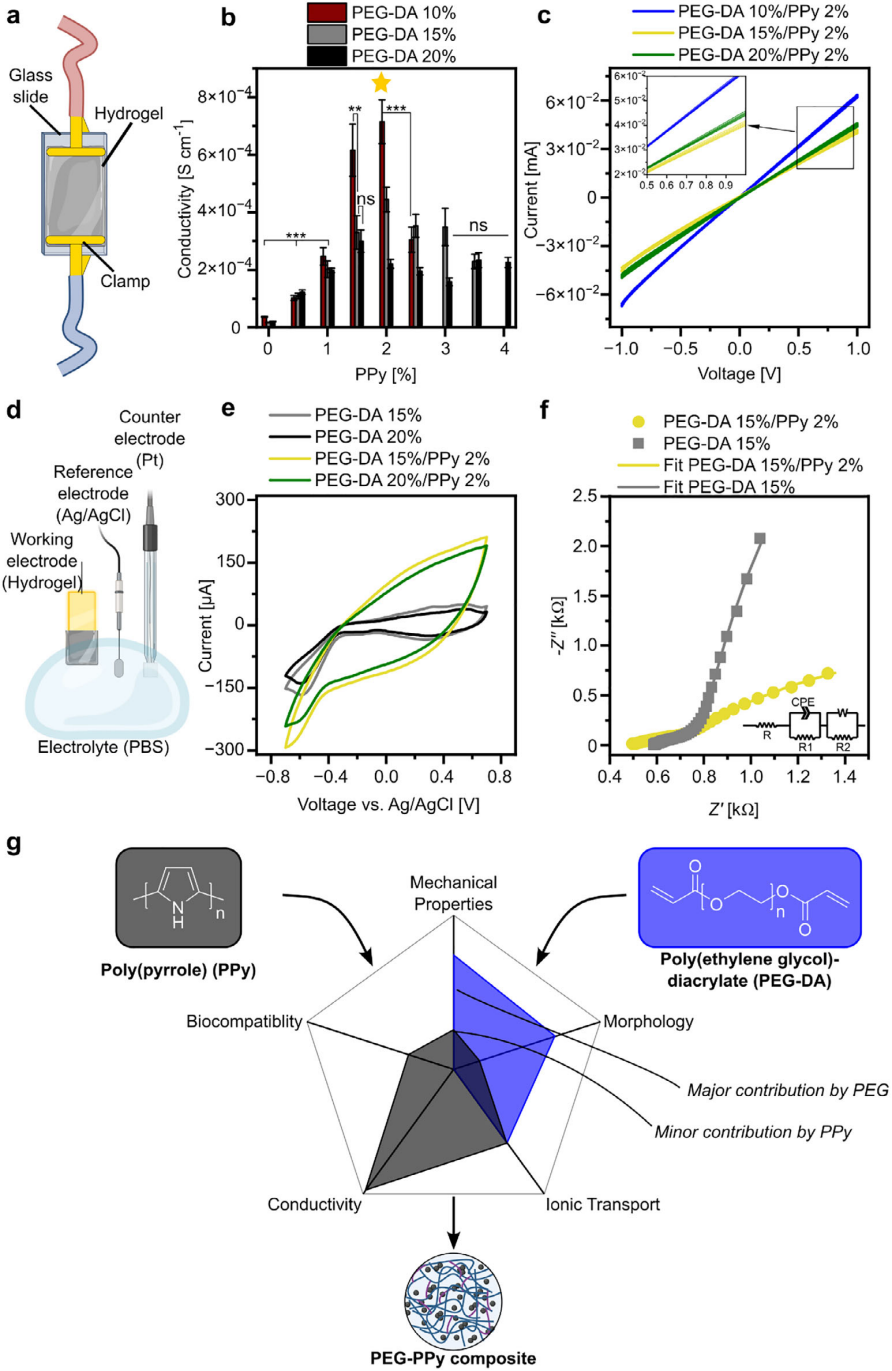
Electrochemical behavior of PEG‐PPy hydrogels. (a) Schematic setup of conductivity measurements. (b) Conductivity of composite hydrogels at various PEG‐DA concentrations and with increasing amount of PPy. The yellow star marks the 2% PPy condition for which we experienced the highest conductivity parameter. Number of technical replicates: 4 *(n* = 4). Data is presented as the mean ± the standard deviation and analyzed by variance analysis (ANOVA) with ns *p* > 0.05, ^**^ *p* < 0.01, ^***^ *p* < 0.001. (c) Current‐voltage characteristics of PEG‐PPy hydrogels using a voltage sweep from −1 to +1 V. (d) Schematic illustration of a three‐electrode setup with the hydrogel utilized as the working electrode, Ag/AgCl as reference electrode, and Pt as counter electrode. (e) CV of PEG‐PPy composites comparing 15% and 20% PEG‐DA as well as incorporation of 2% PPy. (f) Nyquist plot of EIS showing the comparison of a PEG and a PEG‐PPy hydrogel fitted by equivalent circuit modelling. The equivalent circuit of the PEG‐PPy hydrogel consists of a resistor (R) in series with a resistor (R1) and a constant phase element (CPE) in parallel, and a resistor (R2) and a Warburg element (W) in parallel. (g) Schematic illustration of the distinct roles of PEG‐DA and PPy in the hydrogel composite and how they can affect/contribute to certain properties depending on the ratio and concentration. Created with BioRender. Hein, L. (2025) https://BioRender.com/zdjpwlg.

Hydrogels with varying PEG‐DA (10%, 15%, and 20% PEG‐DA) and PPy (0‐4%) concentrations were shaped into rectangular samples for testing. As shown in Figure 4a, the composite hydrogels were placed on a glass slide with gold electrodes attached at both ends. Two probe measurements were conducted, and the conductivity was obtained by relating the resistance (*R*) to the length of the sample (*l*) and the cross‐sectional area (*A*) by Equation (4) [58]:

(4) $$\sigma =A/\left(R\cdot l\right)$$

The resistance measurements indicated that incorporating PPy as a conductive phase into the hydrogel network significantly enhanced the conductivity, independent of the crosslinker concentration (Figure 4b; Figure S15). A stepwise growth in conductivity was observed up until 2% PPy, where the hydrogel network showed peak conductivity values of 7∙10^−4 ^± 7∙10^−5^, 4∙10^−4 ^± 4∙10^−5^, and 3∙10^−4 ^± 5∙10^−5^ S cm^−1^ for 10%, 15%, and 20% PEG‐DA compositions, respectively. In comparison to pure PPy, which can achieve conductivities of up to 10^2^–10^3^ S cm^−1^, these values are 6−7 orders of magnitude lower [57]. However, this represents up to a 25‐fold increase compared to the hydrogels without PPy. A similar material previously reported by Fantino et al. displayed a resistivity of 3.5–0.013 MΩ cm [59]. This translates to a conductivity of 2.85∙10^−7^–7.69∙10^−5^ S cm^−1^. Charge transport in conjugated polymers, such as PPy, is generally described by the Variable Range Hopping (VRH) model [60], where conductivity arises from the hopping of charge carriers between localized states. Therefore, the increase of the conductivity with increasing PPy concentration up to 2% can be related to a better connection of PPy aggregates throughout the PEG‐DA matrix, which facilitates the hopping of mobile charges [60, 61]. However, beyond a PPy concentration of 2%, a noticeable decline in conductivity was observed, which plateaued across all gel compositions. This phenomenon can be attributed to several factors, but primarily inadequate photocuring during the polymerization process. Excessive amounts of PPy seemingly hinder the photopolymerization process and lead to softer, more fragile gels. This was also observed in the mechanical characterization (Figure 3). Overall, these results suggest that the balance between the mechanical strength provided by the crosslinked PEG‐DA network and the electrical performance of PPy is pivotal.

Highest conductivity was achieved at 1.5%−2% PPy compositions, where the balance between the conductive polymer content and the hydrogel's crosslinking density seemed to create the most efficient pathway for charge transport. Given that we identified these concentrations as the optimum, we proceeded to focus on 2% PPy for further analysis.

To assess the electrical behavior in more detail, we measured the current‐voltage (*I*–*V*) characteristics of the gel compositions containing 2% PPy using the same setup employed for the resistance measurements (Figure 4a; Figure S16a). A voltage sweep from −1 to +1 V was applied, and five consecutive measurements were taken to evaluate the stability and reproducibility of the material. The results, shown in Figure 4c, revealed a linear increase in current with increasing voltage, confirming that all samples exhibited near‐ohmic behavior, meaning the resistance remains constant within this voltage range. Furthermore, the *I*–*V* curves from the consecutive measurements demonstrated minimal deviation of less than 2%, indicating that the gels possess excellent electrical stability. Additionally, the influence of the material's flexibility on its electrical performance was evaluated by monitoring the resistance under controlled bending conditions. No statistically significant change in resistance was observed for bending angles up to 90°, confirming that the hydrogel maintains stable electrical behavior under mechanical deformation (Figure S16b–d). This stability is an important attribute for applications in biosensing, where constant performance over time is essential.

The electrochemical behavior of the PEG‐PPy composite hydrogels was evaluated using CV and EIS. Both measurements were performed in a three‐electrode configuration, using a platinum (Pt) counter electrode, a silver/silver chloride (Ag/AgCl) reference electrode, and the PEG‐PPy hydrogel as the working electrode (Figure 4d). The hydrogel was placed between two glass slides coated with a thin layer of gold (100 nm), and it was used as a self‐standing electrode (Figure S17). PBS (1X, pH 7.4) was used as the electrolyte, and 2 mm of the hydrogel electrode were submerged into it. Figure 4e shows the CV measurement, where a quasi‐rectangular shape with no obvious redox peaks is observed, characteristic of capacitive behavior. This indicates that the hydrogels store charge primarily through double‐layer capacitance and/or pseudo‐capacitance [62, 63, 64, 65]. Additionally, the area enclosed by the CV loops increases significantly with the addition of PPy, indicating enhanced charge storage capacity [62]. This is attributed to improved electronic conductivity and swelling properties. EIS [66] data were analyzed by fitting the spectra to the equivalent circuit shown in Figure 4f. The equivalent circuit comprises a series resistance (Rs), followed by two parallel branches: the first (CPE‖R1) accounts for non‐ideal capacitive behavior and charge transfer phenomena occurring at the interfaces; the second (R2‖Warburg) represents processes associated with ion transport and diffusional limitations within the material [66].

The fitting yielded R‐squared values ∼1 for all samples, indicating good agreement (Table 2) between the experimental data and the equivalent circuit. The choice of equivalent circuit was based on two key observations. First, the Nyquist plots exhibit two distinct semicircles (Figure 4f), with the high‐frequency beginning at a real impedance value greater than zero. This suggests a system composed of a series resistor (Rs) connected to two parallel branches. Given the non‐ideal capacitive behavior commonly observed in hydrogels, constant phase elements (CPEs) were used instead of ideal capacitors. Second, during circuit fitting with two CPE elements, we observed that one of the CPE exponents (n) approaches 0.5, which is characteristic of diffusion‐controlled processes [66, 67]. Therefore, it was physically meaningful to replace that CPE with a Warburg impedance element to more accurately describe ion diffusion in the system.

**TABLE 2:** Electrochemical values observed for equivalent circuit fitting of EIS data (Figure 4f).

Sample	Rs [Ω]	R1 [kΩ]	Q [µS s^n^]	n	R2 [Ω]	W [kΩ s^1/2^ ]	R‐squared
PEG‐DA 15%	581.3 ± 0.8	28 ± 4	157 ± 1	0.981 ± 0.006	291 ± 9	2.16± 0.05	0.9999
PEG‐DA 20%	711 ± 1	80 ± 10	105± 1	0.939 ± 0.004	309 ± 5	4.15 ± 0.09	0.9999
PEG‐DA 15% /PPy 2%	491 ± 1	2.8 ± 0.2	370 ± 10	0.75 ± 0.02	270 ± 10	4.3 ± 0.1	0.9997
PEG‐DA 20% /PPy 2%	494 ± 2	2.3 ± 0.1	241 ± 9	0.79 ± 0.02	300 ± 10	5.6 ± 0.3	0.9991

The values for each parameter can be checked on Table 2. Rs, associated with the electrolyte resistance, showed variability between 491 and 711 Ω, likely due to challenges in ensuring consistent submersion of self‐standing hydrogels, an experimental limitation worth noting. The first resistor (R1) represents the ions transport into the hydrogel. It increases from 28 to 80 kΩ with PEG‐DA content due to higher crosslinking density, which restricts ion mobility. With the addition of PPy R1 substantially decreases to 2.8 and 2.3 kΩ, for 15% and 20% of PEG‐DA, respectively, due to the combination of improved swelling and higher electrical conductivity. The CPE parameter Q followed similar trends, decreasing about 35% with PEG‐DA and increasing about 135% with PPy, in agreement with the CV results. The CPE exponent n remained above 0.7, indicating moderately non‐ideal capacitive behavior; notably, n decreased with PPy addition, owing to increased morphological disorder or ion–polymer interactions, despite improved electronic conductivity. The second resistance (R2) increased with PEG‐DA content, consistent with reduced water uptake and a tighter network structure. PPy reduced R2 by forming electronic pathways through the matrix that facilitate ion compensation, thereby ensuring electrostatic neutrality. However, Warburg impedance also increased about 100% with PPy, suggesting hindered ionic diffusion, potentially due to structural effects or electrostatic interactions between ions and the PPy chains.

Overall, the characterization results demonstrate that PEG and PPy play complementary and distinct roles within the composite hydrogel, depending on their relative concentrations. PEG primarily forms the mechanical scaffold, regulating the hydrogel's structural integrity, morphology, and diffusive transport. In contrast, PPy introduces electronic conductivity and modulates ionic transport and biointerface interactions, contributing to the composite's electrochemical functionality (Figure 4g).

### 2.4 Introduction of PEG‐PPy Hydrogels as Gate Electrodes in Organic Electrochemical Transistors (OECT)

**FIGURE 5:**
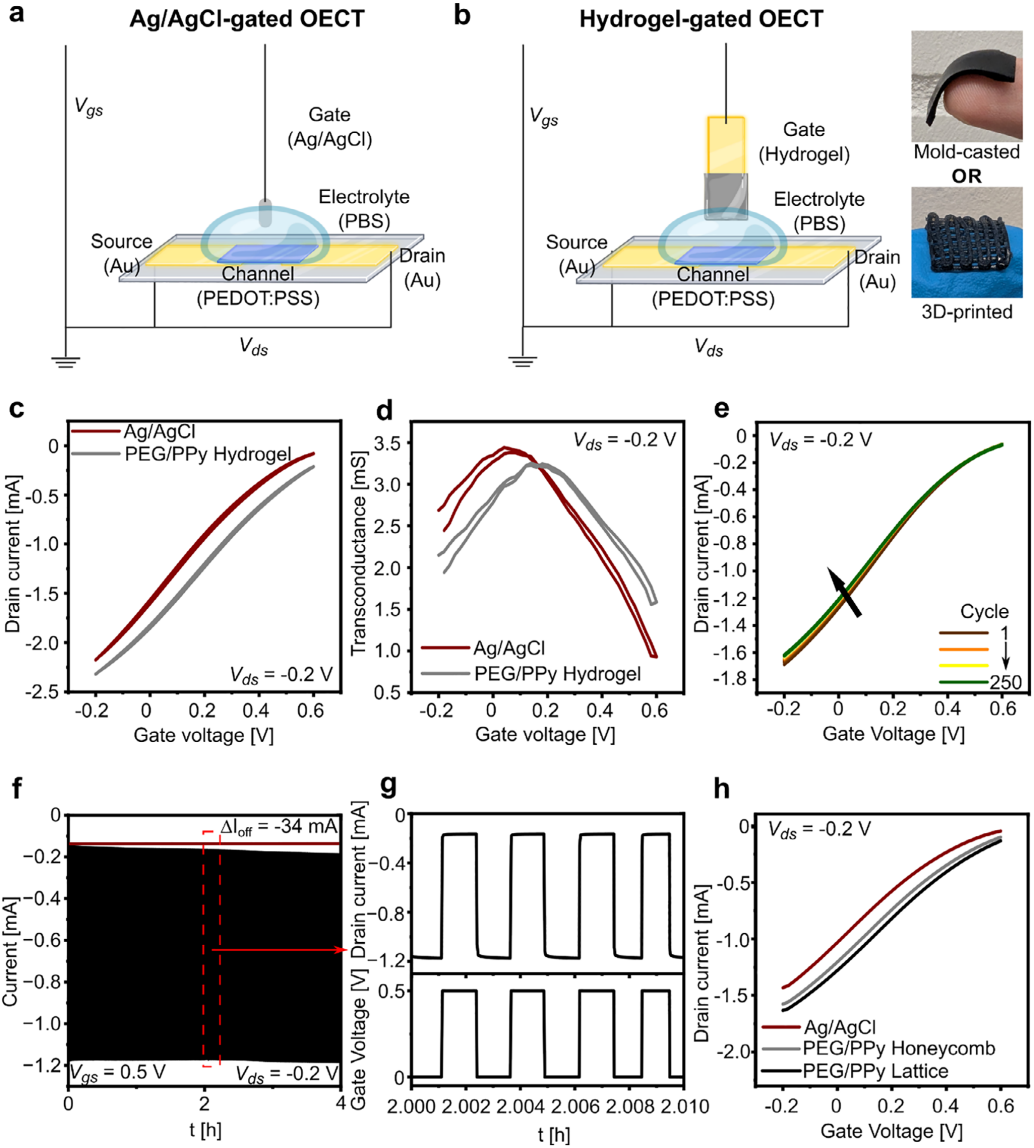
Comparison of OECT performance (a) Schematic setup of a Ag/AgCl‐gated OECT and (b) a hydrogel‐gated OECT (c) Transfer curve and (d) respective transconductance for an PEDOT:PSS‐based OECT gated with a standard Ag/AgCl pellet (red line) and a PEG‐PPy hydrogel (10%–2%) (gray line). Stability of hydrogel‐gated OECT. (e) Transfer curves over 250 cycles. (f) Pulsed measurement over 4 h. (g) Zoom into pulsed measurements showing the pulsed gate voltage and drain current response. (h) Transfer curves of Ag/AgCl‐gated OECT in comparison to 3D‐printed PEG/PPy scaffolds (lattice and honeycomb structure). Created with BioRender. Hein, L. (2025) https://BioRender.com/mtoz7dk.

Considering the electrochemical characteristics of the PEG‐PPy hydrogel, it is a promising candidate to use as a gate electrode in OECTs. OECTs are three‐terminal devices consisting of an organic semiconducting film (channel) defined by two source and drain contacts and connected to a gate electrode through an electrolyte (Figure 5a,b). The channel conductivity is modulated by the gate voltage. Upon application of a gate‐source voltage, ions from the electrolyte are driven into or out of the organic mixed ionic–electronic conductor, resulting in doping or dedoping of the channel [28]. The choice of gate electrode plays a critical role in OECT performance, as it determines how the applied gate voltage is distributed between the gate/electrolyte and electrolyte/channel interfaces. For non‐polarizable electrodes such as Ag/AgCl, the voltage drop at the gate/electrolyte interface is negligible. In contrast, polarizable electrodes such as gold (Au) or semiconducting polymers form a capacitive double layer at the gate/electrolyte interface, which divides the applied gate‐source voltage between the two interfaces. In this case, to minimize voltage loss at the gate, it is required that the gate capacitance is at least ten times larger than that of the channel [28]. Here, we compare the performance of OECTs using either Ag/AgCl pellets or PEG–PPy hydrogels as gate electrodes.

The architecture of the OECTs used here is shown in Figure 5a,b. The drain and the source contacts were fabricated from Cr/Au, the channel consisted of PEDOT:PSS, the electrolyte was PBS solution (1X, pH 7.4), and the gate electrode was either an Ag/AgCl pellet or the PEG‐PPy hydrogel. The channel dimensions were 0.3 mm (width) x 1 mm (length), with the thickness ranging from 62 to 96 nm, the area of the submerged hydrogel was 54 mm. Given the volumetric capacitance of PEDOT:PSS (∼30 F cm^−3^) [37], the channel capacitance of the OECTs ranges from 0.56 to 0.86 µF, which is more than ten times lower than the PEG‐PPy hydrogel capacitance (Table 2). PEDOT:PSS‐based OECTs operate in depletion mode. This means that when a positive gate voltage is applied, cations from the electrolyte migrate within the channel and deplete the charge carriers, decreasing the drain current. This behavior can be seen in transfer curves presented in Figure 5c and in the output and transfer curves in Figure S18. One of the figures of merit of an OECT is the transconductance (*g_m_*), which is calculated from the first derivative of the transfer curves. The transconductance displays how good an OECT can amplify the gate signal. When comparing the transconductance of both tested gate electrodes, almost equal transconductance is observed (Figure 5d, Table 3; Figure S19).

**TABLE 3:** Comparison of the OECT performance for hydrogel‐gated and Ag/AgCl‐gated devices. The PEDOT:PSS channel dimensions were 0.3 mm (width) × 1 mm (length), and PBS was used as the electrolyte.

	Hydrogel‐gate	Ag/AgCl‐gate
g_m_ (max)	3.2 ± 0.1 mS (*n* = 3)	3.6 ± 0.2 mS (*n* = 2)
V_gs_ (g_m_ max)	150 ± 3 mV (*n* = 3)	70 ± 30 mV (*n* = 2)
OCP	93 ± 8 mV (*n* = 3)	0.44 ± 0.05 mV (*n* = 2)
I_on_/I_off_	12 ± 3 (*n* = 3)	31 ± 6 (*n* = 2)
Ig	< µA (*n* = 3)	< µA (*n* = 2)
τ_off_	0.5 ± 0.1 ms (*n* = 3)	0.4 ms (*n* = 1)
τ_on_	0.7 ± 0.2 ms (*n* = 3)	0.7 ms (*n* = 1)
Stability (transfer – 250 cycles)	Δg = – 4.83% (*n* = 1)	Δg = – 4.44% (*n* = 1)

A comparison between hydrogel‐gated and Ag/AgCl‐gated OECTs is provided in Table 3. The Ag/AgCl‐gated devices exhibited a peak transconductance of 3.6 ± 0.2 mS at 70 ± 30 mV, whereas PEG‐PPy hydrogel‐gated OECTs reached 3.2 ± 0.1 mS at 150 ± 3 mV. This positive shift in the transconductance peak is attributed to the different electrochemical potentials of the two gate materials [68, 69]. In fact, the difference in the open circuit potential of 92 ± 8 mV (0.44 ± 0.05 mV for Ag/AgCl and 93 ± 8 mV for the hydrogel gate) is fully consistent with the voltage shift observed in the OECT characteristics (Figure S20).

The I_on_/I_off_ ratio for the hydrogel‐gated OECTs remained in the same order of magnitude (∼10–30) as for Ag/AgCl‐gated devices, and the gate current remained below the µA range for both configurations. Pulsed‐bias measurements were conducted to evaluate turn‐on and turn‐off transient responses (Figure S21), yielding response times of approximately 0.5 ms for OECTs with both electrode systems. Cycling stability measurements (Figure 5e; Figure S22) demonstrated excellent stability with only a 4.83% reduction in transconductance after 250 transfer‐curve cycles, comparable to the 4.44% decrease observed with Ag/AgCl gating. Additionally, under pulsed operation for 4 h (∼7.5k pulses) (Figure 5f,g), hydrogel‐gated OECTs showed only a 4.83% decrease in transconductance. This minimal decrease in electrochemical performance over 250 transfer cycles and 4 h of pulsed operation validates the stability of the PEG‐PPy hydrogel despite the minor structural heterogeneities observed in Figure S5. Furthermore, 3D‐printed hydrogel scaffolds with a honeycomb and lattice structure were fabricated (Figure S23), highlighting that extrusion‐based printing enables the creation of customizable geometries while offering an additive and material‐efficient processing route that is compatible with a wide range of substrates, which may be beneficial for future device integration. When integrated as gate electrodes, these structured hydrogels exhibited electrochemical performance comparable to mold‐cast hydrogels (Figure 5c,h).

Overall, these results confirm that the PEG‐PPy hydrogel is a viable and stable alternative to conventional Ag/AgCl gate electrodes, providing similar transconductance performance and operating in a comparable gate‐voltage range. These characteristics are essential for reliable bioelectronic applications, where signal instability can compromise data quality and diagnostic validity. For further context, OECTs using a PEDOT:PSS gate (Figure S24) exhibited similar transconductance values but required a substantially higher gate voltage (∼0.6 at −0.2 V drain‐source bias) compared to the hydrogel gate.

The hydrogel brings many advantages over the standard Ag/AgCl electrode, especially in biosensing. Due to its elastic/flexible properties as well as its biocompatibility, it is well‐suited for applications on/in the body. In addition, its adjustable stiffness enables the hydrogel to be tailored to the desired tissue. With a facile modification/adjustment of the hydrogel different bioreceptors, such as enzymes, antibodies or aptamers, can be entrapped into the electrode, enabling versatile options for functionalization of biosensors for detecting a broad range of analytes [70]. These findings highlight the promise of PEG‐PPy composites for additive manufacturing, combining printability, mechanical robustness, and electrical conductivity in a single platform for soft electronics and biosensing applications. For comparison, Table S1 summarizes the performance of related hydrogel systems reported in the literature. Notably, our hydrogel represents the only system demonstrated to be extrusion‐printable while simultaneously maintaining tissue‐like mechanical softness and competitive OECT transconductance.

### 2.5 Glucose Oxidase Activity in Enzyme‐Loaded PEG‐PPy Conductive Hydrogels

**FIGURE 6:**
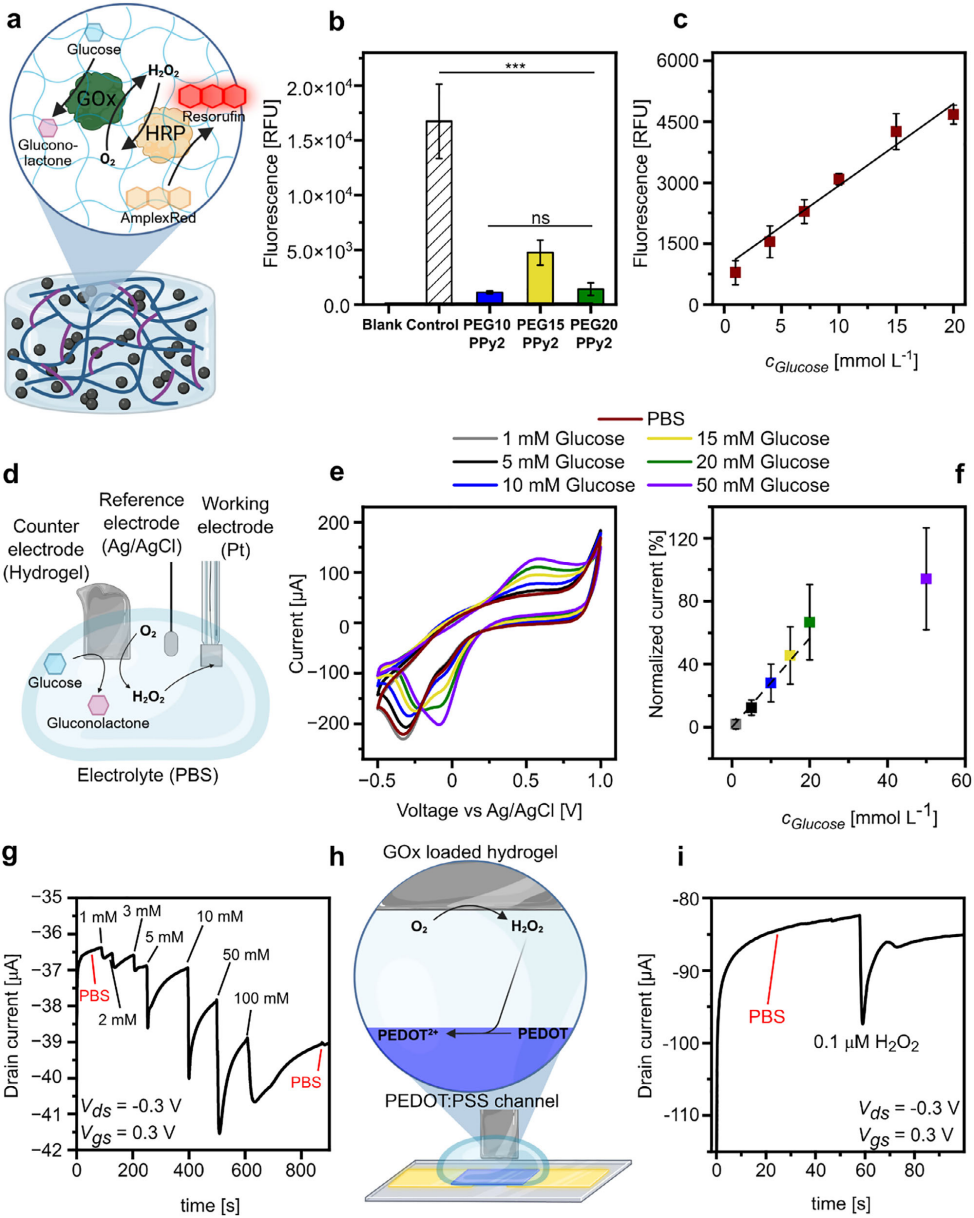
Fluorescence studies of enzymatic entrapped hydrogels. (a) Schematic representation of GOx and HRP encapsulated in PEG‐PPy composite hydrogels. Enzymatic cascade reaction catalyzed by GOx and HRP in the presence of glucose and Amplex Red. (b) Fluorescence assay showing resorufin production from GOx/HRP across various hydrogel compositions (10%–20% PEG‐DA, 2% PPy, 100 mm glucose). (c) Fluorescence assay of resorufin with increasing glucose concentration. Number of repetitions: 3 (*n* = 3). Data is presented as the mean ± the standard deviation and analyzed by variance analysis (ANOVA) with ns *p* > 0.05, ^***^ *p* < 0.001. Electrochemical glucose sensing. (d) Three electrode setup: Working electrode (Pt), counter electrode (hydrogel PEG‐PPy‐GOx), reference electrode (Ag/AgCl). The given concentrations refer to the amount of glucose. (e) Cyclic voltammetry measurements with a potential range of −0.5–1.0 V and a scan rate of 100 mV∙s^−1^. The given concentrations refer to the amount of glucose. (f) Analytical curve of the glucose sensor. The normalized current variation at the oxidation peak is shown as mean ± standard deviation (*n* = 4 devices). (g) Drain current response of a hydrogel‐gated OECT upon addition of various glucose concentrations. PEDOT:PSS channel dimensions: 0.240 mm (width) x 0.080 mm (length). (h) Schematic illustration of the OECT mechanism with H_2_O_2_ oxidizing PEDOT:PSS. (i) Test of H_2_O_2_ addition to a standard Ag/AgCl‐gated OECT (V_ds_ = −0.3 V, V_gs_ = 0.3 V). PEDOT:PSS channel dimensions: 1 mm (width) x 0.3 mm (length). Created with BioRender. Hein, L. (2025) https://BioRender.com/31p3fl6.

After thorough characterization of the PEG‐PPy composite hydrogels, enzymes were incorporated to evaluate their potential for enzymatic biosensing applications. PPy was first mixed in a precursor solution containing polyethylene glycol diacrylate (PEG‐DA) at concentrations of 10%, 15%, or 20% (w/v), along with 2% (v/v) Irgacure 2959 as the photoinitiator. GOx (5 mg mL^−^^1^ in PBS) and horseradish peroxidase (HRP, 1 mg mL^−^^1^ in PBS) were then added at 1% (v/v) each. The enzyme‐loaded solution was cast into molds and photopolymerized, yielding composite hydrogels with enzymes immobilized within the polymer matrix. This immobilization facilitates enzymatic catalysis within the hydrogel network, enabling real‐time detection and quantification of glucose through a coupled enzymatic reaction. Specifically, GOx catalyzes the oxidation of glucose to gluconolactone, generating hydrogen peroxide (H_2_O_2_) as a byproduct, which HRP then uses to oxidize the non‐fluorescent substrate Amplex Red into the highly fluorescent resorufin (Figure 6a) [71, 72]. Fluorescence intensity is directly proportional to glucose concentration, enabling sensitive and specific quantification through the indirect detection of H_2_O_2_ [73]. This validates the potential of the enzyme‐entrapped PEG‐PPy hydrogels for biosensing applications.

To further explore this capability, the enzymatic activity was evaluated across hydrogels containing 2% PPy and varying PEG‐DA concentrations. As shown in Figure 6a, glucose and Amplex Red were introduced to initiate the cascade reaction within the hydrogel, confirming functional enzyme activity. For the assay, 14 mm diameter hydrogel discs were placed in molds submerged in 5 mL of HEPES buffer. D‐(+)‐glucose was added to reach a final concentration of 100 mm and incubated for 10 min at room temperature. Then, 1 mm Amplex Red in DMSO was introduced and allowed to react for 1 min at room temperature. The resulting supernatants were collected and analyzed by fluorescence spectroscopy, compared to blank samples containing only HEPES buffer and controls with enzymes in bulk solution (Figure 6b).

Supernatants from hydrogels without enzymes (lacking both GOx and HRP) established baseline resorufin fluorescence levels of 50, 34, and 41 RFU for 10%, 15%, and 20% PEG‐DA with 2% PPy, respectively, indicating minimal background signal. In contrast, the bulk enzyme solution (containing GOx and HRP) generated significantly higher resorufin fluorescence (16 737 ± 3,388 RFU), attributed to unrestricted diffusion and more efficient enzymatic conversion of Amplex Red to resorufin. Hydrogels showed fluorescence values of 1,116, 4,745, and 1,410 RFU for 10%, 15%, and 20% PEG‐DA, respectively. Although the 15% PEG‐DA hydrogel exhibited the highest fluorescence, differences among the gel compositions were not statistically significant.

To further validate this system for electrochemical sensing, the enzymatic cascade reaction was repeated with varying glucose concentrations (1, 4, 7, 10, 15, and 20 mm), simulating physiological blood sugar levels (Figure 6c). Hydrogel discs were incubated in HEPES buffer containing glucose and 1 mm Amplex Red for 10 min. A linear increase in the fluorescence intensity of resorufin corresponded directly with rising glucose concentrations (Figure 6c), demonstrating that the enhanced fluorescence accurately reflects increased glucose levels and confirming the system's capability to detect glucose within physiologically relevant ranges using fluorescence as a readout.

Following the successful entrapment of GOx within the hydrogel matrix, its enzymatic activity was confirmed by detecting H_2_O_2_, the direct byproduct of glucose oxidation (Figure 6a). As H_2_O_2_ generation is a key intermediate in both fluorescence‐ and electrochemical‐based glucose sensing, its detection serves as essential proof of enzyme functionality within the hydrogel. Building on this, we proceeded to evaluate electrochemical glucose detection as a proof of concept using the three‐electrode setup shown in Figure 6d.

Electrochemical sensing was performed using either CV or chronoamperometry (Figure 6e; Figure S25) [74, 75]. In all cases, composite hydrogels containing immobilized GOx were synthesized as previously described. For these measurements, the concentration of GOx in the pre‐hydrogel mixture was increased from 1% v/v (5 mg mL^−1^) to 12.5% v/v (40 mg mL^−1^) to enhance signal strength.

In the electrochemical cell, a platinum wire served as the working electrode, a standard Ag/AgCl electrode as the reference, and the enzyme‐loaded hydrogel was employed as the counter electrode. This configuration was chosen to satisfy the requirements of the enzymatic glucose‐sensing mechanism. Glucose is first oxidized by GOx within the hydrogel to form gluconolactone, generating H_2_O_2_ as a by‐product. The measurable signal originates from the subsequent electrochemical oxidation of H_2_O_2_ at the working electrode. Since the hydrogel does not efficiently catalyze H_2_O_2_ oxidation and therefore cannot function as a working electrode (Figure S26), platinum, well known for its excellent electrocatalytic activity toward H_2_O_2_ oxidation [76, 77, 78, 79], was selected as the working electrode in this setup.

All measurements were conducted in phosphate‐buffered saline (PBS, 1X, pH 7.4). For chronoamperometric measurements, a constant potential of 0.6 V was applied, while CV experiments were conducted across a potential window of −0.5 to 1.0 V at a scan rate of 100 mV s^−1^. Baseline signals were recorded in PBS prior to glucose addition. Subsequently, glucose was added incrementally to reach final concentrations of 1, 5, 10, 15, 20, and 50 mm. Each measurement was initiated following a 2 min equilibration period.

As shown in Figure 6e, upon incremental glucose addition, the oxidation peak current at 0.6 V increased progressively. This increase reflects the higher amount of H_2_O_2_ generated and subsequently oxidized at the Pt electrode, consistent with the mechanism described above. Additional replicates (*n* = 3) are included in Figure S27. From these four individual samples, the statistical change of the normalized current was determined (Figure 6f). A linear increase is observed, the extent of which depends on the glucose concentration. The limit of detection (LOD) was estimated using Equation (5), based on the International Union of Pure and Applied Chemistry (IUPAC) definition [80]:

(5) $$LOD=\frac{\left[{\left(\frac{\Delta I}{I_{0}}\right)}_{blank}+3\times \sigma -Intercept\right]}{Slope}$$

We determined the LOD as 1.4 ± 0.3 mm. Furthermore, the saturation level is indicated at 50 mm glucose. The increasing error can be attributed to the difficulty of submerging the exact same amount of the hydrogel for each sample.

A similar concentration‐dependent trend was observed in the chronoamperometry measurements (Figure S25). An increase in steady‐state current was observed with increasing glucose concentrations. While 1 mm glucose did not induce a noticeable current shift, at 5 mm the current began to rise. Incremental addition of glucose yielded a stepwise increase in current.

Moreover, as a control experiment, we evaluated three systems: PEG hydrogel with GOx (Figure S28a–d), a PEG hydrogel without GOx (Figure S28e), and a PEG/PPy hydrogel without GOx (Figure S28f). Under cyclic voltammetry measurements, only a small oxidation peak is observed for the PEG hydrogel containing GOx, while both hydrogel variants without Gox show no oxidation peak. These control experiments demonstrate that the ability to entrap enzymes is essential for sensing, while the conductive phase (PPy) enhances sensor sensitivity.

We further evaluated the hydrogel's sensing capabilities in an OECT configuration [74, 81] by incorporating a GOx‐loaded PEG–PPy hydrogel as the gate electrode (Figure 6b). During OECT operation, a drain voltage of −0.3 V and a gate voltage of 0.3 V were applied, while the drain current was monitored over time. In these measurements, the OECTs exhibited an initial transient period (<40 s) followed by a small continuous drift (Figure S29), which is commonly observed in OECT systems [36, 82, 83]. Initial addition of PBS in the electrolyte confirmed system stability, with no observable current shift (Figure 6g). Upon incremental addition of glucose (1 to 100 mm), a clear increase in drain current was observed (Figure 6g). This response is consistent with prior reports and arises from the generation of H_2_O_2_, which can oxidize neutral PEDOT (Figure 6h) [84, 85]. Indeed, direct addition of H_2_O_2_ to the electrolyte to reach a concentration of 0.1 µm in an Ag/AgCl‐gated OECT results in a similar increase in the drain current, as shown in Figure 6i.

Additional sensors with different channel geometries were evaluated, and the corresponding analytical curves, showing drain‐current variations as a function of glucose concentration, are presented in Figure S30. In all cases, glucose detection was clearly demonstrated. Although some variation in signal magnitude was observed, the overall sensing trends remained consistent, and channel geometry did not appear to be the dominant factor governing the response.

We attribute the minor differences primarily to variations in GOx retention within the hydrogel (Figure S31) and to typical influences in enzymatic sensing systems, such as local diffusion dynamics and gradual enzyme activity changes related to oxidative by‐products or pH microgradients [86, 87]. Importantly, the data support that hydrogel‐gated OECTs benefit from the intrinsic signal amplification of OECTs and are therefore capable of reaching lower detection limits than conventional three‐electrode sensors [74].

PEG–PPy hydrogels were loaded with enzymes to test their suitability for biosensing applications. The GOx‐loaded PEG–PPy hydrogels exhibited excellent performance in both fluorescence and electrochemical‐based glucose detection, functioning reliably as either counter or gate electrodes. In the conventional three‐electrode configuration, the sensors provide a clear, concentration‐dependent response with a low detection limit (1.4 mm) and reproducible operation, validating the effectiveness of the enzymatic hydrogel design. The strong correlation between glucose concentration and signal output highlights the material's capability as a fully soft, conductive, and biocompatible sensing interface. In addition to glucose detection, the hydrogel's tunable composition, extrusion printability, and operational stability establish it as a versatile and scalable platform.

However, the system also exhibits certain limitations. The current platform is best suited for short‐term measurements where signals are evaluated relative to a baseline. As illustrated in Figure S31, enzyme leakage may compromise long‐term sensing performance, particularly under conditions requiring repeated or continuous operation. Additionally, the observed drift in OECT measurements (Figure S29) may limit reliability during prolonged operation. Addressing these limitations through material optimization, particularly by enhancing enzyme immobilization, will be essential for enabling continuous glucose monitoring applications.

## 3 Conclusion

In this study, we successfully developed and characterized PEG–PPy composite hydrogels and inks tailored for applications in soft bioelectronics. The materials demonstrated excellent rheological properties, enabling high‐resolution extrusion‐based 3D printing with structural fidelity and stackability, exceeding nine layers, independent of PPy content. Bulk hydrogels were further optimized by systematically varying the crosslinking density (PEG‐DA 10%–20%) and PPy concentration (0%–4%), resulting in a broad tunability of mechanical properties ranging from ∼15 to ∼100 kPa – closely mimicking soft biological tissues such as myocardium and dermis. Notably, the lower stiffness range, rarely explored in bioelectronic materials, expands the application potential of these hydrogels in compliant tissue interfaces. Throughout all formulations, high biocompatibility (>90%) was consistently maintained, as confirmed by live/dead assay using NIH‐3T3 fibroblasts.

The hydrogels also exhibited promising long‐term stability, with minimal degradation observed over 70 days for higher crosslinking densities, and further stabilization achieved by PPy incorporation. Electrical characterization revealed peak conductivity values up to 8∙10^−^^4^ S cm^− 1^ at 2% PPy and 10% PEG‐DA. Electrochemical impedance spectroscopy provided insight into the underlying equivalent circuit model, confirming both electrical integrity and predictable impedance behavior.

Demonstrating their practical utility, the PEG–PPy hydrogels were employed as gate electrodes in OECTs, achieving a transconductance of 3.19 mS at 150 mV, comparable to conventional Ag/AgCl pellet electrodes (3.6 mS at 67 mV). Furthermore, the hydrogel‐gated OECTs exhibited excellent stability, showing less than a 5% decrease in transconductance after 250 transfer‐curve cycles and after 4 h of pulsed‐mode operation. This suggests that with a minor increase in gate voltage, soft hydrogel‐based electrodes can effectively replace traditional rigid metal counterparts. Importantly, the hydrogel offers substantial advantages over Ag/AgCl and Au, including improved biocompatibility, mechanical tunability, and facile chemical functionalization, making it a superior alternative for interfacing with biological systems.

As a proof of concept, we demonstrated enzyme functionalization of the hydrogel with GOx, enabling glucose detection in both electrochemical cells and OECT platforms. Cyclic voltammetry and chronoamperometric measurements showed a LOD of 1.4 ± 0.3 mm, with a saturation up to 50 mm, while OECT‐based sensing allowed detection down to 1 mm. These detection windows encompass the physiologically relevant glucose concentration range (5–20 mm).

In summary, the PEG–PPy hydrogel platform presents a versatile, soft, and biocompatible material system that unifies printability, electrochemical functionality, and biological interfacing. Its capacity to substitute standard rigid electrodes while offering biochemical adaptability holds strong promise for advancing the field of bioelectronics, particularly in personalized diagnostics, wearable devices, and implantable biosensors.

## 4 Experimental Section/Methods

### 4.1 Materials

Poly(ethylene glycol) diacrylate (PEG‐DA, average M_n_ = 700 g mol^−1^), phosphate buffered saline (PBS, pH 7.4) solution, *N*‐2‐hydroxyethylpiperazine‐*N*‐2‐ethane sulfonic acid (HEPES, 1 m), Amplex Red, *D*‐(+)‐glucose, Horseradish peroxidase (HRP, lyophilized powder, ∼150 U mg^−1^), Glucose oxidase (Type VII, lyophilized powder, ≥100 000 units g^−1^), ethylene glycol, dodecylbenzenesulfonic acid (DBSA) and (3‐glycidyloxypropyl)trimethoxysilane (GOPS) were purchased from Sigma–Aldrich. Pyrrole was purchased from Alfa Aesar. Poly(3,4‐ethylenedioxythiophene): polystyrene sulfonate (PEDOT:PSS) was purchased from Heraeus (Heraeus PH1000). Water free Ferric (III) chloride was purchased from Fluka Chemie AG. 2‐hydroxy‐4′‐(2‐hydroxyethoxy)‐2‐methylpropiophenon (Irgacure 2959) and Pluronic F127 were purchased from BASF. Calcein AM and propidium iodide (PI) were purchased from ThermoFisher Scientific (Invitrogen). All aqueous solutions were prepared with Milli‐Q/Millipore water. No further purification was used unless stated.

### 4.2 Poly(pyrrole) (PPy) Synthesis

PPy was synthesized by an oxidative polymerization following the literature procedure (Seike et al.) with minor modifications [39]. Prior to synthesis pyrrole was distilled to remove residual impurities and stabilizing agents. A round‐bottom flask was purged with nitrogen and 150 mL water were added to the flask. 1.546 mL pyrrole were added to the solution and stirred at ambient temperature. Meanwhile, 8.49 g FeCl_3_ were mixed in a beaker with 45 mL water. The suspension was shortly mixed and then slowly added to the monomer containing solution. The reaction is carried out under N_2_‐atmosphere and room temperature for 22 h. The precipitate was collected and purified by multiple cycles (8x) of centrifugation (3k rcf, 10 min) & redispersion in water. Finally, the polymer is lyophilized for 72 h, yielding a black powder in 88% yield. The final product is characterized by solid‐state hydrogen magic angle spinning nuclear magnetic resonance spectroscopy (^1^H‐MAS‐NMR), scanning electron microscopy (SEM), and Fourier‐transformed infrared spectroscopy (FT‐IR):

^1^H‐MAS‐NMR: δ [ppm] = 11 (N‐H), 7 (aromatic‐H).

FT‐IR: 3450 cm^−1^ (N‐H), 2930/2850 cm^−1^ (C‐H, asymmetric), 1550 cm^−1^ (C‐C), 1450 cm^−1^ (C‐N), 1040 cm^−1^ (C‐H).

SEM: spherical particles with 550 nm diameter average.

### 4.3 Nuclear Magnetic Resonance (NMR)

^1^H MAS spectroscopy measurements were performed using a *Bruker Avance III* solid‐state NMR spectrometer operating at 700 MHz 1H Larmor frequency with 25 kHz MAS, 100 kHz rf, and 3 ms CP contact. The spectra were analyzed with MNova software.

### 4.4 Scanning Electron Microscopy (SEM)

Electron microscopy was carried out by applying the sample onto a sticky EM‐holder. Non‐adherent particles were removed by blowing nitrogen onto the sample. The morphology of the particles was recorded using a *Zeiss GeminiSEM 560*. The images were analyzed with ImageJ software.

### 4.5 Fourier Transform Infrared Spectroscopy (FT‐IR)

IR spectra were obtained on a *Bruker VERTEX 70* FT‐IR spectrometer and processed with *OPUS software* to obtain baseline‐corrected absorbance spectra. The KBr pellets were prepared by mixing 5 mg of sample with 360 mg of finely powdered potassium bromide immediately before each measurement.

### 4.6 Pre‐Hydrogel Mixture Preparation

Various concentrations of the hydrogel‐forming polymer PEG‐DA and the conductive polymer PPy were tested. For this purpose, different precursor solutions were prepared by mixing PEG‐DA solution (10, 15, or 20 wt.%) with previously synthesized PPy (0–4 wt.%) and 2% v/v photoinitiator solution (Irgacure 2959, EtOH/H_2_O (7:3), 20 wt.%). The solution is first sonicated at room temperature for 5 min and then stirred for 10 min.

### 4.7 Hydrogel Development

PEG‐PPy composite hydrogels were developed by transferring the pre‐hydrogel mixture into a polystyrene mold of different dimensions and irradiating it with UV‐light in a *honle LEDcube100IC* UV‐chamber (*λ* = 365 nm, *I* = 50% (≈ 135 mW/cm^2^)) for 120 s, *H_e_*  =  16.2 J cm^−2^).

### 4.8 Swelling Analysis

Photoinitiated PEG‐PPy hydrogels were synthesized as described above, submerged in MiliQ‐water and stored in the fridge. The water was exchanged after 24 and 48 h. The gels were swollen for a total of 72 h and weighted after reaching swelling equilibrium (*m_q_*). Then the gels were dried at 50°C for 72 h. The gels were weighted again after being fully dried (*m_d_*). The swelling (Q) is determined by Equation (6) [88]:

(6) $$Q\;\;\left[\%\right]=\frac{m_{q}-m_{d}}{m_{d}}\;\cdot 100\%$$

### 4.9 Gel Content Analysis

To determine how much polymer was incorporated into the hydrogel network the gel content (*G*) was analyzed. As mentioned above, the synthesized hydrogels were cleansed from residual substrates by washing/swelling them in ultrapure water for 48 h. The gels were dried at 50°C for 72 h and weighted after being fully dried (*m_d_*). The dried mass (*m_d_*) was compared to the initial mass of precursor (*m_p_*) by utilizing Equation (7) [89]:

(7) $$G\;\left[\%\right]=\frac{m_{d}}{m_{p}}\;\cdot 100\%$$

### 4.10 Degradation Study

PEG‐PPy hydrogels (14 mm diameter) were developed and measured in weight immediately after. The gels were submerged in 2 mL solution of either PBS‐solution (1X, pH 7.4) or cell culture medium (DMEM (high‐glucose) + 10% h.i. FBS + 1% P/S). Afterward the gels were incubated at 25°C or 37°C. Weight assessment of the hydrogels were carried out after 24, 48, 72 h, 6, 7, 9 d, 2 weeks. Afterward, the hydrogels were measured weekly for a total of 2 months. For a measurement, the hydrogel was transferred out of their solution and residual liquid was removed. The normalized weight (*N*) was determined by Equation (8) [90]:

(8) $$N=\frac{m_{t}}{m_{t=0}}$$

### 4.11 Conductivity Measurements

To assess the conductivity of the synthesized PEG‐PPy composite hydrogels, the material was cut into a rectangle form. The form factor of each sample was measured by a caliper. Then the sample was placed on a glass slide, and two electrodes were attached. A voltage was applied utilizing a *Keithley 2000* Multimeter. The conductivity value of each sample is accessible by the measured resistance (*R*), the length of the sample (*l*) and the cross‐section of electrode and hydrogel (*A*) using Equation (9) [58]:

(9) $$\sigma =A/\left(R\cdot l\right)$$

### 4.12 Current‐Voltage Characteristics

PEG‐PPy composite hydrogels were prepared in a similar manner as for the conductivity measurements. The gels were cut into rectangle shape, placed onto a glass slide, and two electrodes were attached. A voltage sweep, from −1 to +1 V, was carried out utilizing a *Keithley 4200 semiconductor parameter analyzer*. The sweep was repeated for a total of 5 times.

### 4.13 Rheology

Hydrogels were cut to 8 mm discs using a biopunch. The sample was placed on a *Discovery HR‐3 hybrid rheometer* with a plate‐plate geometry (8 mm) and a solvent trap filled with water. First, all gels were analyzed by dynamic frequency sweeps (DFS) in the range of 0.1–100 rad s^−1^ and a constant oscillation strain of 0.1%. Then a dynamic strain sweep (DSS) was initiated in the range of 0.01%–10% and a constant frequency of 10 rad s^−1^. For all gels the linear viscoelastic regime (LVR) was found between 2.5–15 rad s^−1^ frequency and 0.01%–0.2% strain. Hence, dynamic time sweeps (DTS) were carried out using 0.1% oscillation strain, 5 rad s^−1^ frequency, and a constant axial force of 0.3 N. All measurements were carried out at 25°C or 40°C.

Inks incorporating PEG and PPy were also evaluated for the 3D bioprinting process. Inks were preserved on ice prior to the rheology test. Around 50 µL of the inks were transported to the plate‐plate geometry (8 mm) and a solvent trap filled with water. All inks were analyzed with DSS with frequency fixed at 10 rad s^−1^ and temperature at 37°C. 0.3% strain was found to be within LVR for all the inks and was selected for future test. Furthermore, DTS is carried out with a frequency set to 10 rad s^−1^, temperature set to 37°C, and oscillation strain altering between 0.1% and 100% every 1 min.

### 4.14 Cell Culture

Murine embryo fibroblast cells (NIH‐3T3, ACC 59) were routinely cultivated in Dulbecco's Modified Eagle's Media (DMEM high‐glucose (gibco), with 10% heat inactivated fetal bovine serum (h.i. FBS) and 1% penicillin‐streptomycin (P/S, gibco)). Cell cultures were stored in an incubator at 37°C and 5% CO_2_. Cells were passaged weekly in a standard procedure: The cells were first treated with 4 mL trypsin/EDTA‐solution (0.25%, gibco), incubated for 5 min at 37°C, 5% CO_2_ and 5 mL DMEM + 10% h.i. FBS was added. The suspension was centrifuged at 300 rcf for 5 min. After centrifugation, the supernatant was removed and the cells were redispersed in 1 mL cell culture medium. Cell count was assessed utilizing a *Countess II automated cell counter*. Approximately 1∙10^5^ cells were seeded into a 75 mL flask with 12 mL cell culture medium.

### 4.15 Live/Dead Assay

General assessment of cytotoxicity and biocompatibility of the PEG‐PPy composite hydrogels was carried out in a LIVE/DEAD assay. Approximately 20 000 murine embryo fibroblast cells (NIH‐3T3, ACC 59, P.13) were seeded in a polystyrene plate and incubated at 37°C, 5% CO_2_ overnight. The material was cut to pieces of 40 mg. All gels were sterilized by storing in ethanol‐solution (70%) for 10 min. Subsequently, the gels were washed multiple times with PBS‐solution to remove residual ethanol. Then a sterilized hydrogel sample was carefully placed on the 2D‐cell culture and exposed to it for 20 h at 37°C, 5% CO_2_. The positive and negative controls were not exposed to any hydrogel sample. After 20 h of incubation the hydrogel samples were removed from the cell cultures. The negative control was treated with 1 mL 70% ethanol for 5 min. All cells were treated with 200 µL PBS‐solution containing 1 µm Calcein AM (ThermoFisher Scientific) and 1 µm PI (ThermoFisher Scientific) for 13 min at ambient temperature. Afterward, the wells were rinsed with PBS‐solution twice. The fluorescent cells were imaged under a *Leica TCS SP5 II* confocal laser scanning microscope (CLSM) with stimulated emission depletion (STED) utilizing a 10x dry objective in 2x – 5.92x zoom. Fluorescence of calcein‐stained viable cells was observed by excitation with a 488 nm laser line and measuring the emission between 502 and 546 nm. On the other hand, fluorescence of PI‐stained dead cells was detected by excitation with a 561 nm laser line and examination of emission between 591 and 790 nm. Cell viability was evaluated from counting the live and dead cells using ImageJ software. The cell viability was determined utilizing following Equation (10) [43].

(10) $$Viability\;\;\left[\%\right]=\frac{n^{\circ }\;\mathrm{of}\;\mathrm{alive}\;\left(\mathrm{green}\right)\;\mathrm{cells}}{{\mathrm{total}\;n}^{\circ }\;\mathrm{of}\;\left(\mathrm{stained}\right)\;\mathrm{cells}\;}\;\cdot 100\%$$

### 4.16 Enzyme Loaded Composite Hydrogels

To entrap enzymes like GOx and/or HRP in the hydrogel matrix the pre‐hydrogel mixture preparation was slightly altered. As described above PEG‐DA solution of various concentrations (10–20 wt.%) were mixed with PPy (0 or 2 wt.%) and 2% v/v photoinitiator solution. After sonication and stirring, 1% v/v GOx‐solution (5 mg mL^−1^ in PBS) and 0.6% v/v HRP‐solution (1 mg mL^−1^ in PBS) were added. The hydrogel synthesis was carried out as stated above. Enzyme loaded hydrogels were stored in the fridge in PBS‐solution.

### 4.17 Fluorescence Studies

Enzyme loaded PEG‐PPy composite gels (14 mm diameter) were synthesized in various PEG‐DA concentrations (10–20 wt.%) and PPy amounts (0 or 2 wt.%). After synthesis, gels were transferred into a polystyrene mold and submerged in 900 µL 4‐(2‐hydroxyethyl)‐1‐piperazineethanesulfonic acid (HEPES, 5 mm). 100 µL *D*‐(*+*)‐glucose solution (1 m) were added and the enzymatic reaction was carried out for 10 min. Afterward 2 µL AmplexRed solution (1 mm in DMSO) were added and reaction was carried out for another 5 min at ambient temperature. Then the supernatant was removed and analyzed in a plate reader. HEPES‐solution (5 mm) was utilized as a reference. For plate reader analytics the solutions were linearly shaken for 5 s with 1 mm amplitude and 1440 rpm frequency. A wavelength of *λ* = 555 nm (10 nm bandwidth, monochromator) was used for excitation and emission was measured at *λ* = 595 nm (10 nm bandwidth, monochromator). The study was continued by repeating the enzymatic reaction with different concentrations of glucose (20, 15, 10, 7.5, 4, 1 mm).

### 4.18 Enzyme Leakage Study

GOx‐Cy5–loaded hydrogels (10 wt.% PEG‐DA, 2 wt.% PPy) were prepared following the standard synthesis protocol, substituting 1% v/v GOx solution (5 mg mL^−^^1^ in PBS) with 1% v/v Cy5‐labeled GOx solution of the same concentration. The hydrogels were immersed in 3 mL PBS (1×, pH 7.4) and stored under gentle static conditions. At 1, 2, 3, 7, 10, and 14 days, aliquots of the supernatant were collected and analyzed using a microplate reader, with PBS serving as the blank and GOx‐Cy5 solution as the reference standard. Samples were linearly shaken for 5 s (1 mm amplitude, 1440 rpm) prior to measurement. Fluorescence was recorded by excitation at *λ*  =  600 nm and emission was measured at *λ*  =  670 nm (10 nm bandwidth each). Raw fluorescence intensities were baseline‐corrected using the blank signal and subsequently normalized to the reference solution to determine the percentage of GOx released into the supernatant over time.

### 4.19 Ink Preparation

The ink for 3D printing consists of 2 g PEG‐DA, 4 g Pluronic F127, 0.05 g photoinitiator (Irgacure 2959) and PPy in 10 g of Milli‐Q water. The solution was thoroughly mixed on ice and transferred into a 10 mL syringe. The ink was preserved in 4°C until further use.

### 4.20 3D Bioprinting

The inks were printed using a extrusion based 3D bioprinter (Brinter One, Brinter, USA). A stainless steel needle gauge of 18G was used for extrusion and a temperature controlled tool was selected to keep the ink temperature to 37°C. The pressure was fined tuned for smooth flow of filament. Printing speed set to 6 mm s^−1^. After the printing, the printed construct is moved into the UV chamber (honle LEDcube100IC, λ = 365 nm,I = 135 mW cm^−2^) for 120 s.

### 4.21 Printability

Printability was evaluated based on the methods of Ouyang et al. cylinders with the diameter of 15 mm and infill density of 50% were printed using 18GA needle. Every cylinder consists of two layer of grid structure, and the circularity of the enclosed area is defined as the Equation (11):

(11) $$C=\frac{4\pi A}{L^{2}}$$

where *L* means perimeter and *A* means area. For a square the circularity is equal to π/4. The printability is defined based on Equation (1).

When the pattern is printed as desired, the enclosed area wound demonstrate a square shape, and the printability would be close to 1 [40].

### 4.22 Compression Properties

Cylinders with the diameter of 15 mm and height of 5 mm were printed using the previous inks with 100% infill, followed by UV irradiation under the same condition. Compression properties of the final printed products are evaluated through a universal testing machine (*ZwickRoell*, Germany) equipped with a 50 N loadcell, and a 10 mm diameter indentation compression module. A pre‐load of 0.02 N was set with 2 mm min^−1^ pre‐load speed. The compression speed was 5 mm min^−1^ and maximum compression strain was 60%.

### 4.23 Electrochemical Measurements

CV, EIS and open‐circuit potential (OCP) were performed in a three‐electrode configuration using a potentiostat (*PalmSens4*). A Pt electrode acted as the counter electrode, an Ag/AgCl pellet was utilized as the reference electrode and the hydrogel was used as a self‐standing working electrode, unless otherwise specified. CV measurements were performed within a potential range between −0.7 and 0.7 V at a scan rate of 100 mV s^−1^. EIS measurements were performed in OCP (50 kHz to 0.5 Hz, Eac 10 mV). For all the measurements, a PBS solution was used as electrolyte.

### 4.24 Glucose Sensing

Similar to the electrochemical measurements, glucose sensing was carried out by measuring the current change in a three‐electrode setup by either CV or chronoamperometry. In both cases an enzyme loaded composite hydrogel was first manufactured as described above. For electrochemical measurements we increased the amount of GOx‐solution utilized for synthesis from 1% v/v (5 mg mL^−1^) to 12.5% v/v (40 mg mL^−1^). The hydrogel was attached to a glass slide coated with 5 nm Cr and 50 nm Au used as a self‐standing electrode. Measurements were carried out by immersing the working electrode (Pt), the reference electrode (Ag/AgCl) and the counter electrode (hydrogel) in PBS (1X, pH 7.4). CV and chronoamperometry were carried out with a *PalmSense4* Potentiostat/Galvanostat. For cyclic voltammetry a potential in the range of −0.5–1.0 V with a scan rate of 100 mV s^−1^ and for chronoamperometric measurements a potential of 0.6 V was applied and measurements were carried out for 60 s. Glucose sensing was done by adding a glucose solution (1 m in PBS) to the electrolyte to obtain a 1, 5, 10, 15, 20, and 50 mm glucose solution respectively. 1 and 2 min after each addition, the measurement was run. The limit of detection (LOD) was estimated using Equation (12), based on the International Union of Pure and Applied Chemistry (IUPAC) definition [80]:

(12) $$LOD=\frac{\left[{\left(\frac{\Delta I}{I_{0}}\right)}_{blank}+3\times \sigma -Intercept\right]}{Slope}$$

where (Δ*I*/*I*_0_)_blank_ corresponds to the average relative signal change observed for the blank condition, defined as the addition of pure PBS. Since this value was negative, the blank was normalized to zero to ensure a physically meaningful result. The term σ represents the relative standard deviation of the blank response, measured as 2%. The intercept and slope were obtained from the linear fit of the analytical curve shown in Figure 6f.

### 4.25 OECT

Organic electrochemical transistors were fabricated using a standard microfabrication approach, as described in previous reports [91, 92]. Briefly, Cr/Au (7 nm/100 nm) source and drain contacts were deposited on cleaned glass substrates and patterned using photolithography, followed by a lift‐off process. Subsequently, the substrates were coated with 2 parylene layers: the first served as an insulating protection to the gold contacts, while the second one acted as a sacrificial layer. The channel regions were then defined using a combination of photolithography and O_2_ plasma etching processes. A PEDOT:PSS suspension (Heraeus PH1000) with the addition of 5% of ethylene glycol, 0.1% dodecylbenzenesulfonic acid (DBSA), and 1% (3‐glycidyloxypropyl)trimethoxysilane (GOPS) was deposited by spin coating at 3000 rpm for 60 s. After spin coating, the sacrificial layer was peeled off, removing any PEDOT:PSS outside of the channel, and the devices were annealed at 140°C for 1 h. Finally, the OECTs were immersed in ultra‐pure water overnight to remove excess low‐molecular‐weight molecules and PSS. The geometries of the channel were 1 mm (width) x 0.3 mm (length), 0.240 mm x 0.080 and 0.1 mm x 0.050 mm, with a film thickness ranging from 62 to 96 nm.

Electrical measurements of the OECTs were conducted using a *Keithley 2604B* source meter controlled via *SweepMe! software*, phosphate buffered saline (PBS) solution as electrolyte, and either an Ag/AgCl pellet or the self‐standing hydrogel as gate electrode. Output curves (I_D_ vs. V_DS_) were obtained by sweeping V_DS_ from 0.1 to −0.5 V at gate voltages (V_GS_) ranging from −0.1 to 0.5 V in 0.1 V increments. Transfer curves (I_D_ vs V_GS_) were obtained by sweeping V_GS_ from −0.2 to 0.6 V at drain voltages (V_DS_) stepped from −0.2 to −0.4 V in −0.2 V increments. The stability test using transfer curves was performed by sweeping V_GS_ from −0.2 to 0.6 V for 250 cycles, using a constant V_DS_ = −0.2 V. Pulsed measurements were performed by applying a constant V_DS_ = −0.2 and a square‐wave pulse at the gate electrode with V_GS_ = 0.5 V. The period of the pulse was 0.1 s for the transient measurement and 0.01 s for stability tests, both with a 50% duty cycle.

### 4.26 Statistical Analysis

Analysis of the data was carried out with *MS Excel* and *Origin*. All data points are displayed as the statistical mean ± the standard deviation. For swelling analysis, gel content, degradation, rheology measurements and fluorescence studies three samples were used. For conductivity measurements and cell‐viability, 4 and 2 technical replicates were used respectively. The obtained values were analyzed by Analysis of Variance (ANOVA). Non‐significance is displayed by ns *p* > 0.05. Significance is indicated by ^*^ *p* < 0.05, ^**^ *p* < 0.01, and ^***^ *p* < 0.001.

## Author Contributions

L.H. and R.C. are co‐first/equal authors and contributed equally to this work. M.V. conceived and designed the project. L.H. and M.V. performed all the materials synthesis, hydrogel development and characterization (physicochemical, mechanical, electrical and electrochemical). R.C. and U.K. conceived, designed and performed the electrical, electrochemical characterization and glucose sensing, as well as the conceptualization, fabrication, experimentation and analysis of OECT. L.H. and R.C. conducted glucose sensing experiments to demonstrate proof of concept. X.W. and M.V. was responsible for preparing the ink, performing rheological and compression measurements of the ink and hydrogel, and 3D printing. L.H., T.I. and M.V. performed GO activity in enzyme‐loaded hydrogels. L.H., R.C., X.W., T.I., U.K. and M.V. analyzed the data. L.H., R.C., X.W., K.L, U.K., and M.V. wrote the original manuscript, which was proofread and approved by all authors. U.K., K.L., and M.V. supervised the project and secured funding.

## Funding

The authors acknowledge funding by the Max Planck Society, Carl Zeiss Stiftung (InteReg) (project number P2024‐02‐015), and Daimler and Benz Foundation (no. 32‐09/24). X. W. acknowledge the financial support from China Scholarship Council. This work was also supported by the Max Planck Graduate Center (MPGC) with the Johannes Gutenberg University Mainz.

## Conflicts of Interest

The authors declare no conflicts of interest.

## Supporting information




**Supporting File**: advs75412‐sup‐0001‐SuppMat.docx.

## Data Availability

The data that support the findings of this study are available from the corresponding author upon reasonable request.
