# Supplementary material for: Printable Conductive Hydrogels for Electrochemical Biosensing and Soft Bioelectronic Interfaces
Source: Adv Sci (Weinh). 2026 Apr 23;13(39):e21216. doi: 10.1002/advs.202521216 (PMC13335752; doi:10.1002/advs.202521216)
Supplement: Supplementary file 1 — Supporting File: advs75412‐sup‐0001‐SuppMat.docx. [file ADVS-13-e21216-s001.docx]

**Supporting Information**

**Printable Conductive Hydrogels Enable Electrochemical Biosensing and Soft Bioelectronic Interfaces**

Lukas Hein^×^, Renan Colucci^×^, Xin Wei, Tsvetomir Ivanov, Katharina Landfester*, Ulrike Kraft*, Maria Villiou*

L. Hein, R. Colucci, X. Wei, T. Ivanov, K. Landfester, U. Kraft, M. Villiou

Max Planck Institute for Polymer Research (MPIP), Ackermannweg 10, 55128 Mainz, Germany

E-mail of corresponding author K. Landfester: [landfester@mpip-mainz.mpg.de](http://landfester@mpip-mainz.mpg.de)

E-mail of corresponding author U. Kraft: kraftu@mpip-mainz.mpg.de

E-mail of corresponding author M. Villiou: villioum@mpip-mainz.mpg.de

*Corresponding Authors

^×^These authors contributed equally to this work

**Table of contents**

| Section | Page |
| --- | --- |
| S1. Synthesis and Characterization of Poly(pyrrole) (PPy) | 2 |
| S2. Physicochemical Characterization of PEG-PPy Hydrogels | 4 |
| S3. Printability of PEG-PPy Hydrogels | 6 |
| S4. Rheological Analysis of Printable Inks | 7 |
| S5. Mechanical characterization of PEG-PPy Hydrogels | 8 |
| S6. Electrical and electrochemical Properties of PEG-PPy Hydrogels | 12 |
| S7. PEG-PPy Hydrogel as a Gate Electrode in OECTs | 15 |
| S8. Glucose Oxidase Activity in Enzyme-Loaded PEG-PPy Conductive Hydrogels | 19 |
| S9. References | 24 |

**S1. Synthesis and Characterization of Poly(pyrrole) (PPy)**

As described in the Materials and Methods part poly(pyrrole) (PPy) was synthesized following the procedure of Seike et al with minor modifications. ^[1]^ Briefly, water (150 mL) and freshly distilled pyrrole (1.546 mL) were added to a round-bottom flask purged with nitrogen. Meanwhile FeCl3 (8.49 g) were dissolved in 45 mL water and slowly added to the monomer containing solution. The reaction is carried out under N2-atmosphere and room temperature for 22 h. The precipitate was collected and purified by multiple cycles of centrifugation & redispersion in water. Finally, the polymer is lyophilized for 72 h yielding a black powder in 88% yield (**Figure S1-3**).

Then, we analyzed the black powder PPy by magic angle spinning proton nuclear magnetic resonance spectroscopy (^1^H-MAS-NMR**, Figure S1**) as well as Fourier-transformed infrared spectroscopy (FT-IR; **Figure S2**) and scanning electron microscopy (SEM, **Figure S3**). The high electrical conductivity of the material led to the resonance circuit becoming out of tune and the sample heating during rotation. We therefore estimate a measurement temperature of > 40 °C. The resulting NMR spectra reveals two broad peaks at around 7 ppm (aromatic-H) and 11 ppm (N-H).


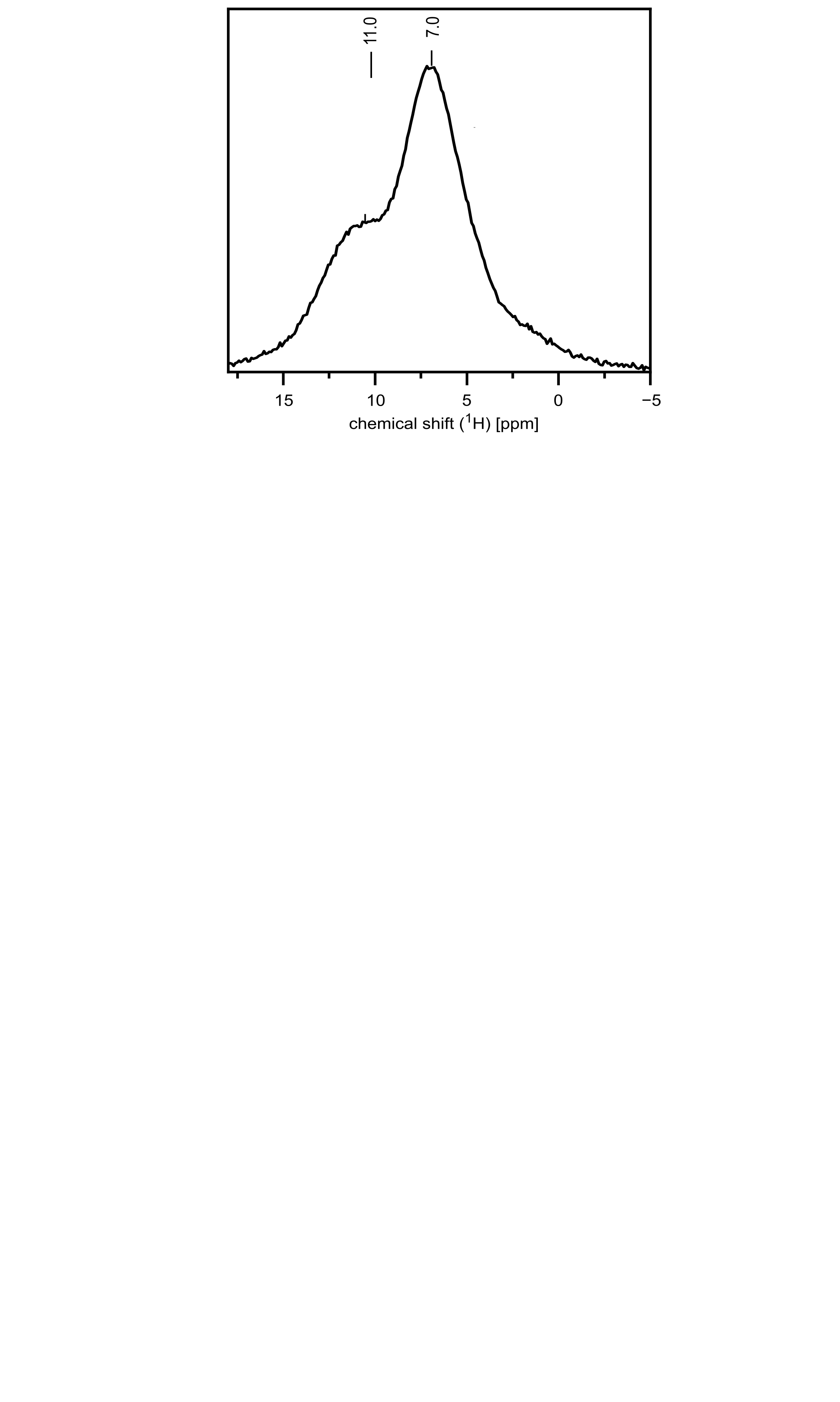


**Figure S1.** ^1^H-NMR-spectra of synthesized poly(pyrrole) performed on a Avance III solid-state NMR spectrometer operating at 700 MHz, 1H Larmor frequency with 25 kHz MAS, 100 kHz rf, and 3 ms CP contact. The NMR displays two broad peaks at 7.0 ppm and 11.0 ppm, which can be associated to the aromatic protons in the backbone and the amine group respectively.

Fourier-transformed infrared spectroscopy (FT-IR) was carried out by mixing 5 mg polymer with 360 mg KBr and forming a pellet. The infrared spectra were obtained by a Bruker VERTEX 70 FT-IR spectrometer. We observed several distinct peaks, which indicate the successful synthesis of poly(pyrrole). 3450 cm^-1^ (N-H), 2930/2850 cm^-1^ (C-H, asymmetric), 1550 cm^-1^ (C-C), 1450 cm^-1^ (C-N).


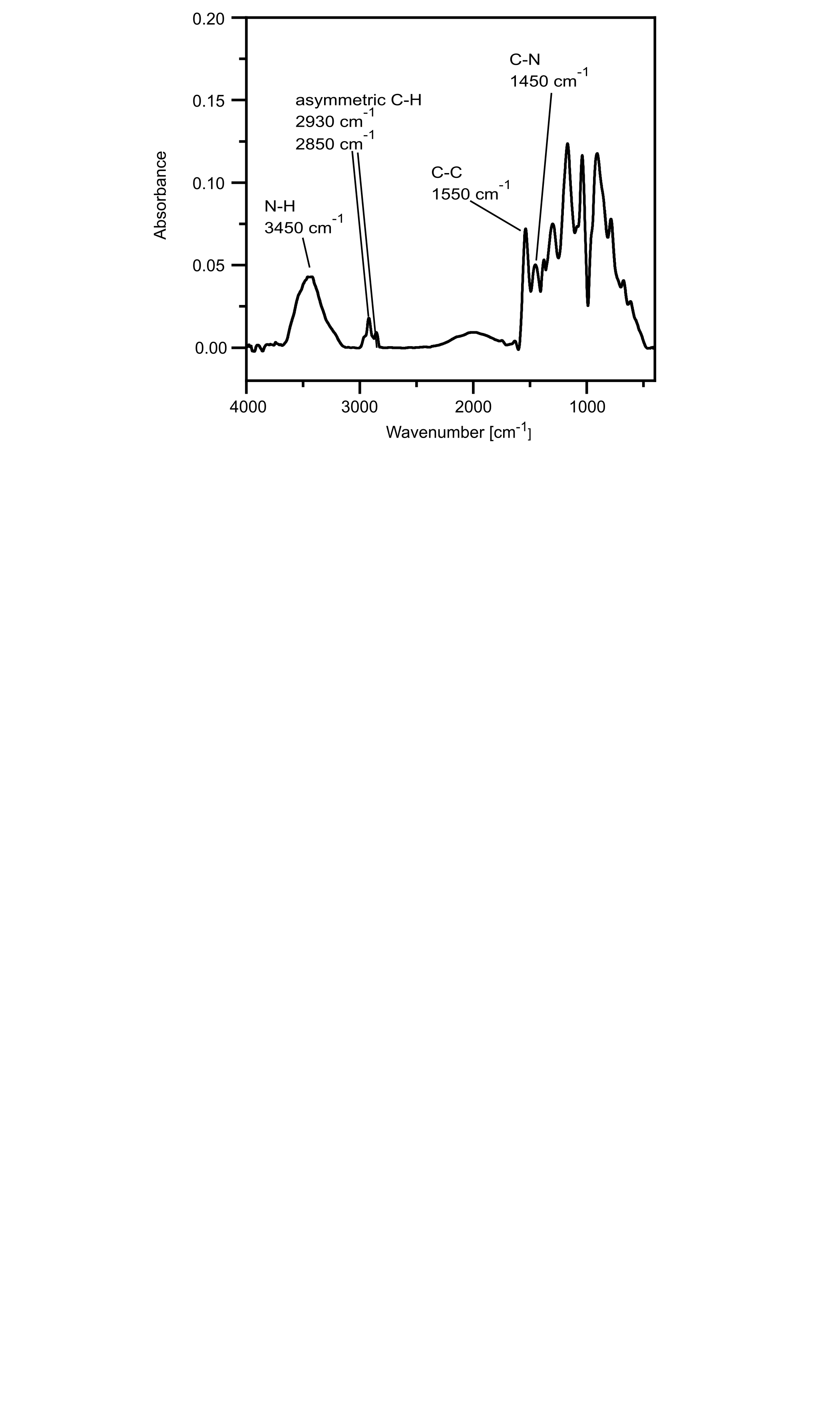


**Figure S2.** FT-IR-spectra of synthesized poly(pyrrole) performed on a Bruker VERTEX 70 FT-IR spectrometer.

Electron microscopy was carried out by applying sample onto an EM-holder. Non-adherent particles were removed by blowing nitrogen onto the sample. The morphology of the particles was recorded using a *Zeiss GeminiSEM 560*. We observed nanometer sized particles, which form agglomerates in their dry state. The images were analyzed with ImageJ software to calculate the average particle size.


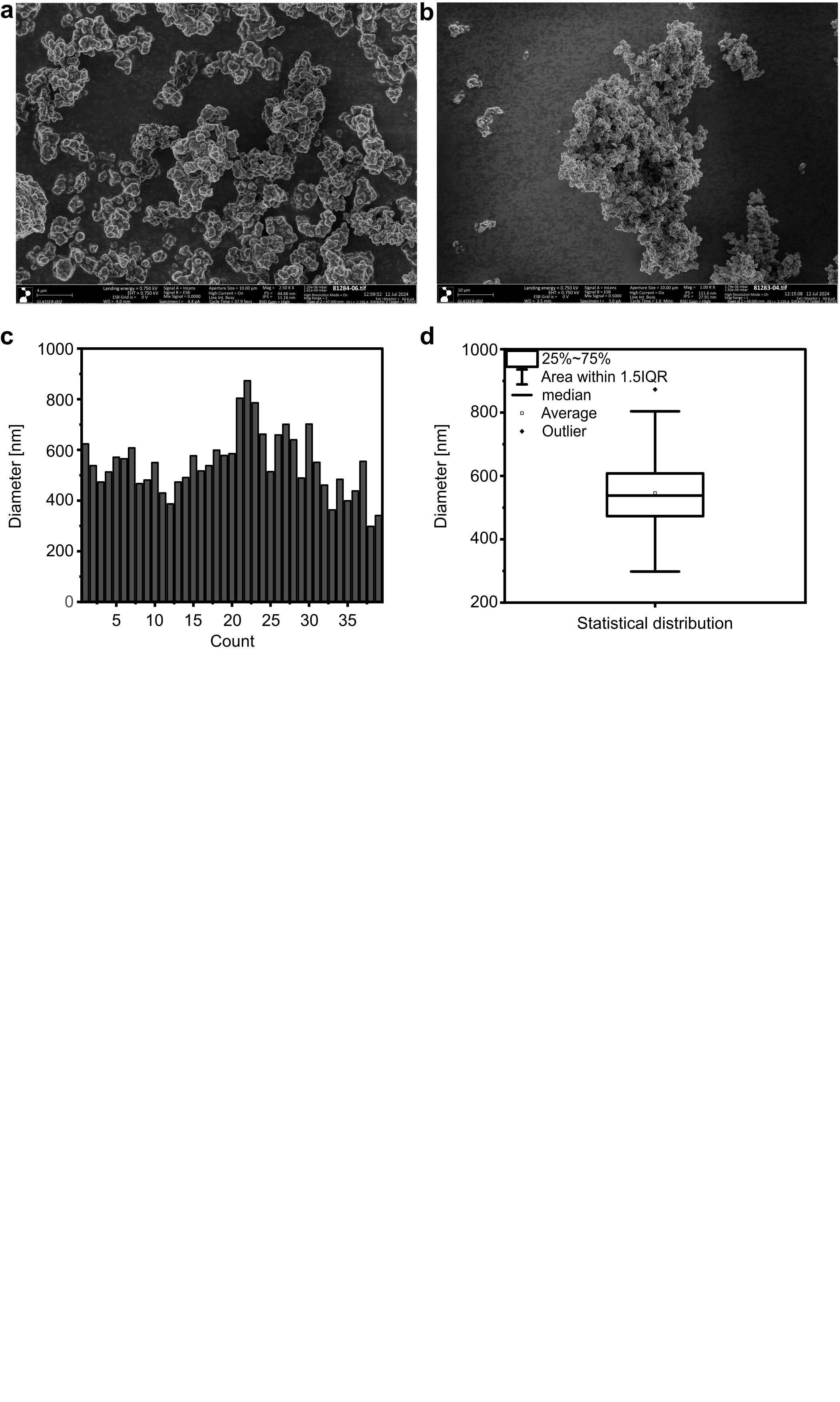


**Figure S3.** Scanning electron microscopy (SEM) of poly(pyrrole) particles recorded on a *Zeiss GeminiSEM 560.* Size analysis was carried out by ImageJ. 40 particles were measured in diameter and statistical analysis was carried out by Origin. An average diameter of 550 nm was determined.

**S2. Physicochemical Characterization of PEG-PPy Hydrogels and inks**

To prepare PEG–PPy composite hydrogels, the conductive polymer poly(pyrrole) (PPy) was dispersed in a solution of poly(ethylene glycol) diacrylate (PEG-DA, *M*_n_ = 700 g/mol), using 2-hydroxy-4′-(2-hydroxyethoxy)-2-methylpropiophenone (Irgacure 2959, I2959) as the photoinitiator. The mixture was then photopolymerized under UV irradiation to form a poly(ethylene glycol)-based hydrogel matrix embedded with conductive PPy particles. Macroscopic inspection of the hydrogels reveals progressively darker coloration with increasing PPy content (**Figure S4**).

**Figure S4.** Hydrogel formation with increasing amount of PPy. Hydrogel formation was achieved in standard procedure. With increasing amounts of PPy, the yielded composite gel became more black.


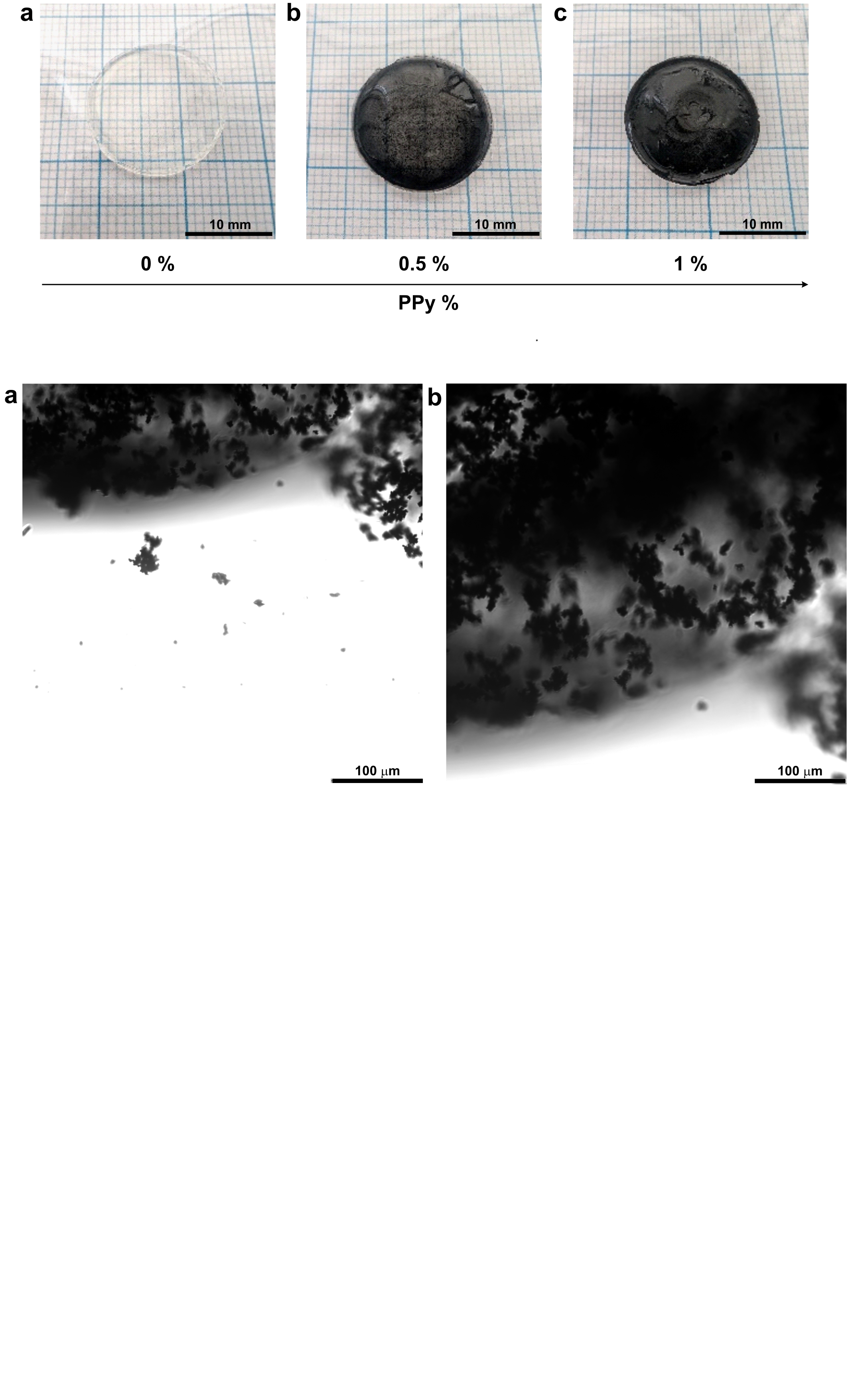


Microscopic analysis of the resulting composite revealed distinct black PPy agglomerates within the hydrogel matrix (**Figure S5**).


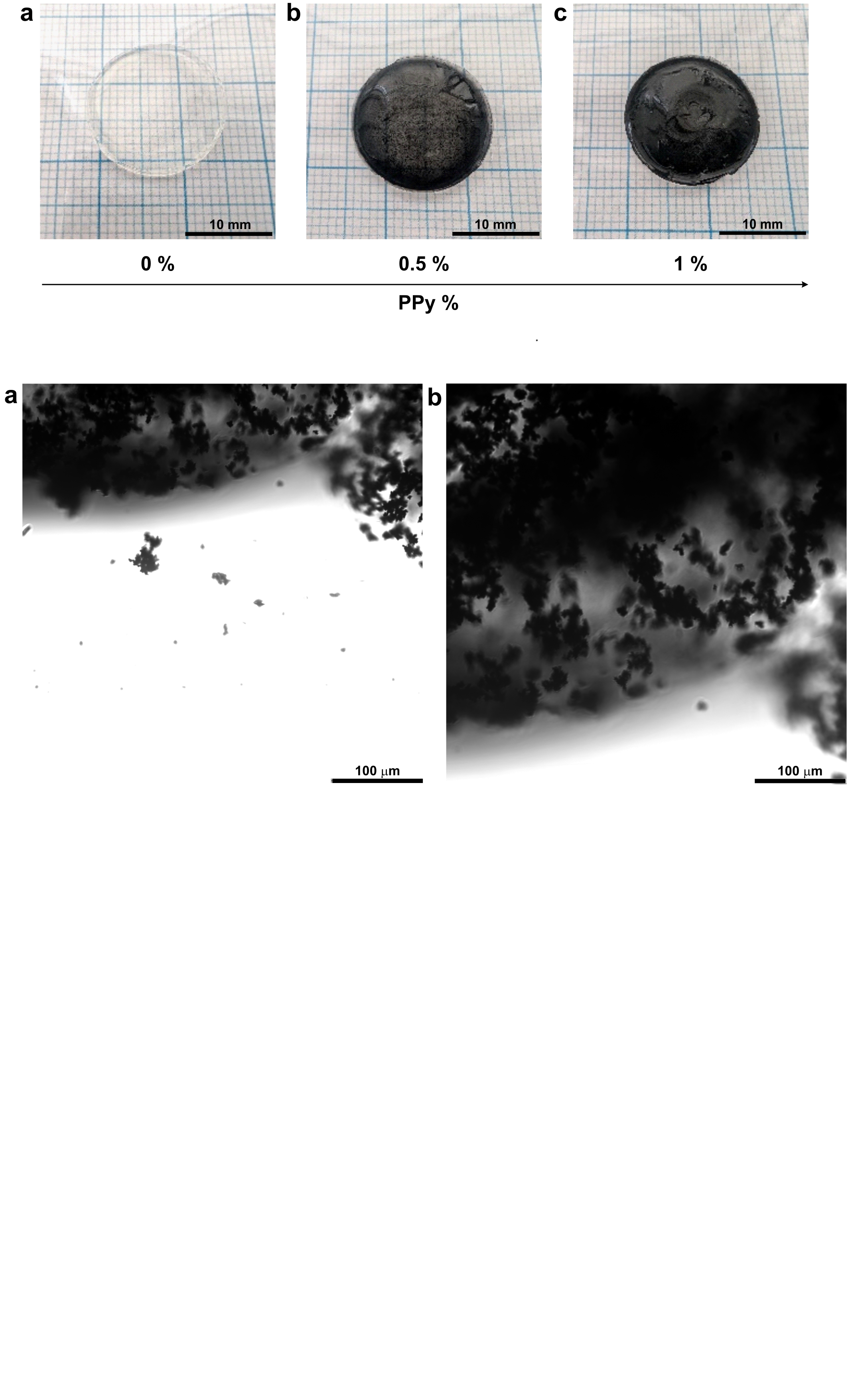


**Figure S5.** PEG-PPy hydrogels under microscope utilizing a 10x dry objective in 3.5-5.6x zoom. The images show the edge of a composite hydrogel, making the small PPy agglomerations inside of it visible. We found that the agglomerates of the conductive polymer can become micrometer sized as is also reported by Seike et al. ^[35]^

The hydrogels exhibit inherent flexibility and slight adhesiveness, which becomes evident when applied to the skin. Their ability to adhere without causing irritation further indicates their biocompatibility and non-toxic nature (**Figure S6**).

**Figure S6.** PEG-PPy hydrogels applied to the skin. The hydrogel shows inherent flexibility and can be bent without inducing structural damage or compromising its integrity.


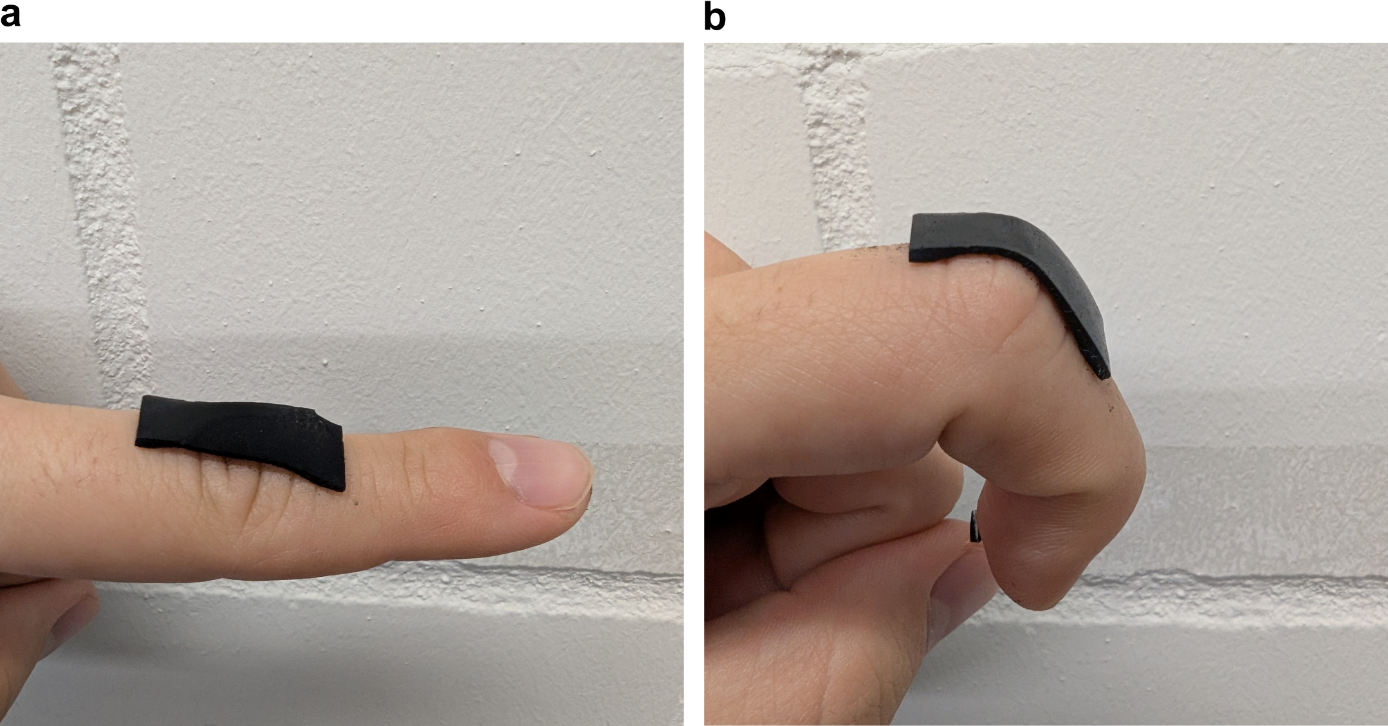


**S3. Printability of PEG-PPy Hydrogels**

To enable extrusion-based 3D printing, the composite formulation was optimized by adding Pluronic F-127 to adjust viscosity and induce shear-thinning behavior (**Figure S12**). Rheological tests confirmed gel-like properties with reversible sol–gel transitions under strain, supporting smooth extrusion up to 2% PPy content (**Figure S13**). This allowed the fabrication of multi-layered structures (up to 9 layers) with high shape fidelity (**Figure S12b-c**).


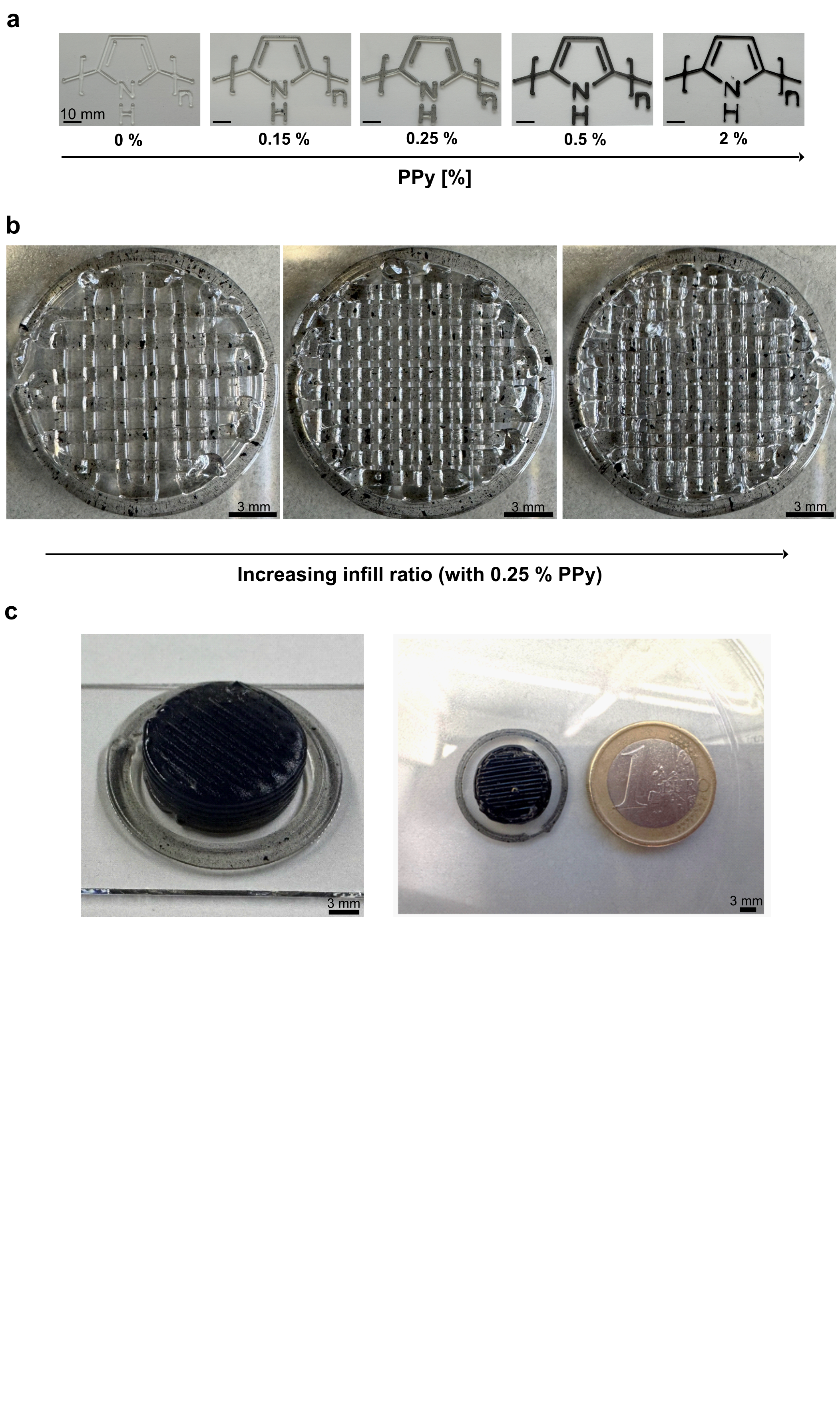


**Figure S7. Extrusion-based 3D-printings of PEG-PPy ink.** **a.** 3D-printing of chemical structure of PPy with increasing amounts of PPy inside the ink. **b.** 3D-printings of PEG-PPy ink (0.25 wt% PPy) with increasing infill ratio. **c.** Multilayered 3D-printings wit PEG-PPy ink (0.5 wt%) with up to 9 layers. (ii) Multilayered structure in comparison to 1€-coin.

**S4. Rheological Analysis of Printable Inks**

Rheological analysis revealed that adding 0.15% PPy increased the ink’s storage modulus from 17 to ~30 kPa, indicating enhanced network stiffness (**Figure S8**). However, higher PPy concentrations (0.5% and 2%) reduced the modulus to ~12 and ~18 kPa, respectively. Compression tests on UV-crosslinked, 3D-printed hydrogels (100% infill) showed the highest strength at 0.15% PPy (101.44 ± 1.97 kPa), consistent with the rheology data, suggesting PPy acts as a physical crosslinker. At 2% PPy, strength dropped sharply (24.53 ± 2.29 kPa), likely due to reduced UV penetration. These results support PEG–PPy composites as promising materials for 3D-printed, conductive, and tunable hydrogel sensors.


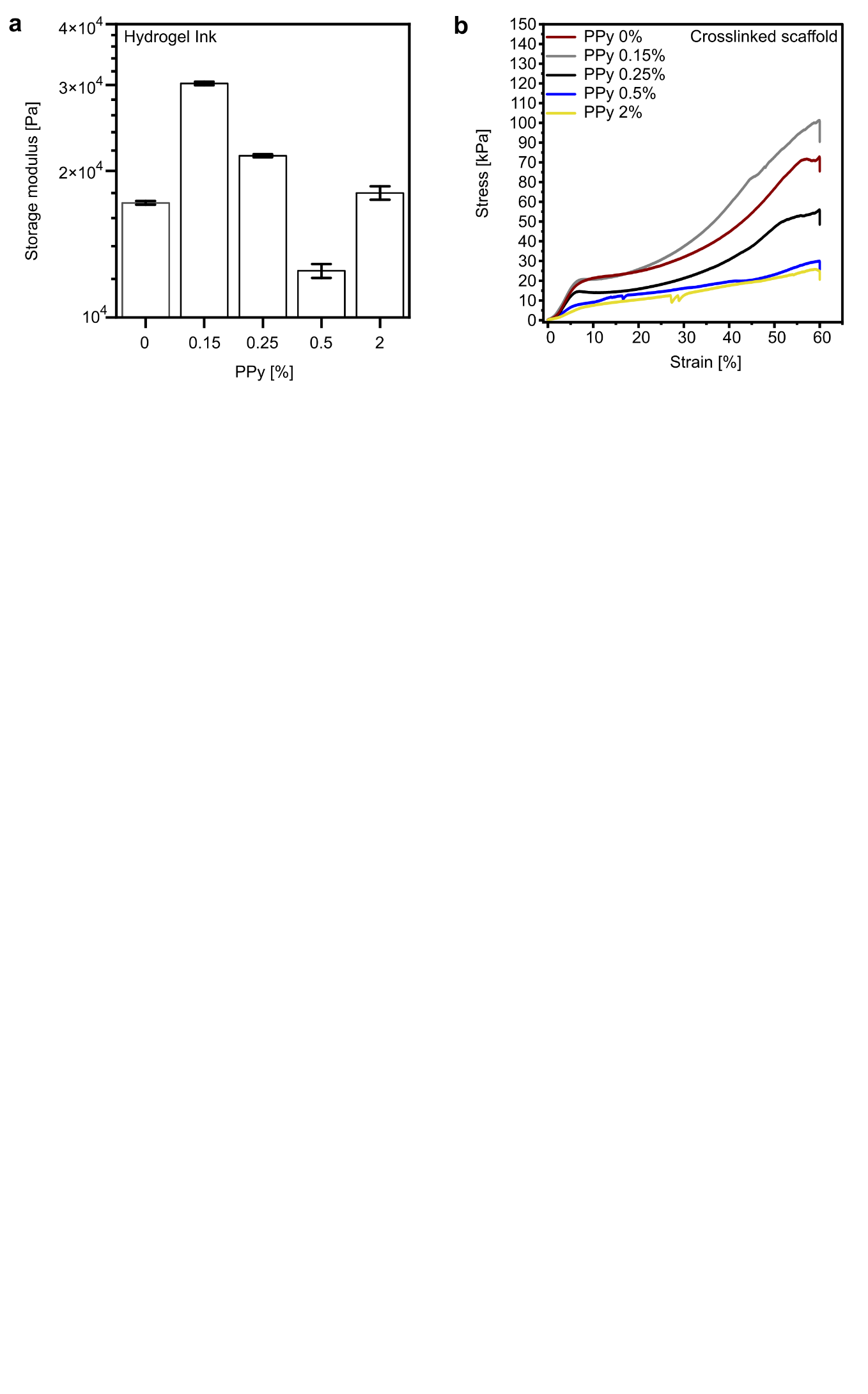


**Figure S8.** Mechanical characterization of hydrogel ink and photopolymerized scaffolds. **a.** Storage modulus of PEG-PPy inks before photopolymerization. **b.** Stress-strain curves (compression test) of PEG-PPy scaffolds after printing and photopolymerization.

**S5. Mechanical characterization of PEG-PPy Hydrogels**

To determine the amount of polymer incorporated into the 3D-network hydrogel matrix the gel content (*G*) was analyzed (**Figure S9**). The synthesized hydrogels were submerged in MilliQ-water for 48 h at ambient temperature to remove residual substrates/unreacted monomers. Then, the gels were dried at 50 °C for 72 h and weighted after being fully dried (*m_d_*). The dried mass (*m_d_*) was compared to the initial mass of precursor (*m_p_*) by utilizing following **equation S1**. ^[2]^

$G\left[ \% \right]=\frac{m_{d}}{m_{p}}\cdot100 \%$ (S1)


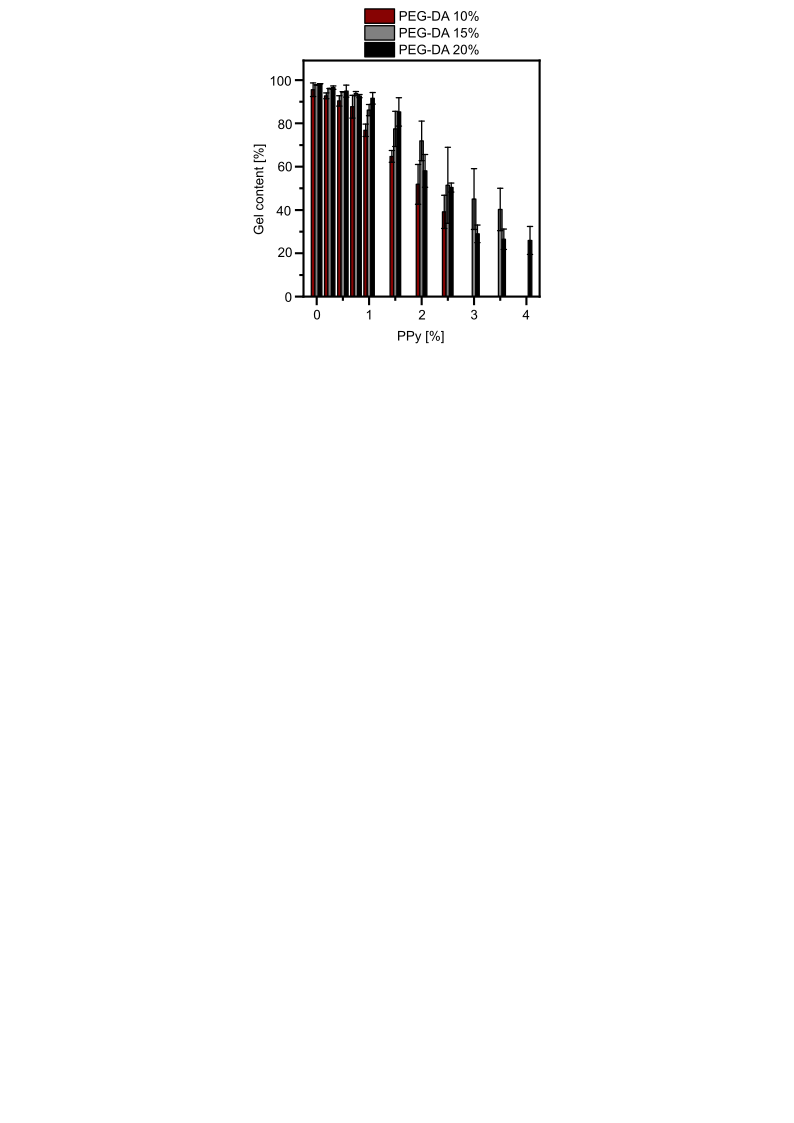


**Figure S9.** Gel content of PEG-PPy hydrogels for different crosslinking densities (PEG-DA 10 wt%, 15 wt%, and 20 wt%) as well as various amounts of conductive polymer PPy (0 wt% – 4 wt%). Number of samples: 3 (*N*= 3). Data is presented as the mean ± the standard deviation and analysed by variance analysis (ANOVA) with ns *p* > 0.05, **p* < 0.05, ***p* < 0.001

To determine the mechanical properties of the synthesized hydrogels rheology experiments were performed. Hydrogels were cut to 8 mm discs using a biopunch. The sample was placed on a *Discovery HR-3 hybrid rheometer* with a plate-plate geometry (8 mm) and a solvent trap filled with water. To determine the linear viscoelastic regime a dynamic frequency sweep (DFS) and a dynamic strain sweep were carried out for each PEG-DA concentration at a constant axial force of 0.3 N and 25 °C. In the dynamic frequency sweep the oscillation strain was kept constant at 0.1% and the frequency in a range of 0.1 rad s^-1^ − 100 rad s^-1^ was analyzed. We concluded for all three gels that the LVR was observed as a constant storage modulus (G’) found between 2.5 rad s^-1^− 15 rad s^-1^ frequency. Then a dynamic strain sweep (DSS) was initiated in the range of 0.01% − 10%, while the frequency was kept constant at 10 rad s^-1^. Again, for all three gels we observed similar behavior and concluded that the linear viscoelastic regime (LVR) was found between 0.01% − 0.2% strain (**Figure S10**).


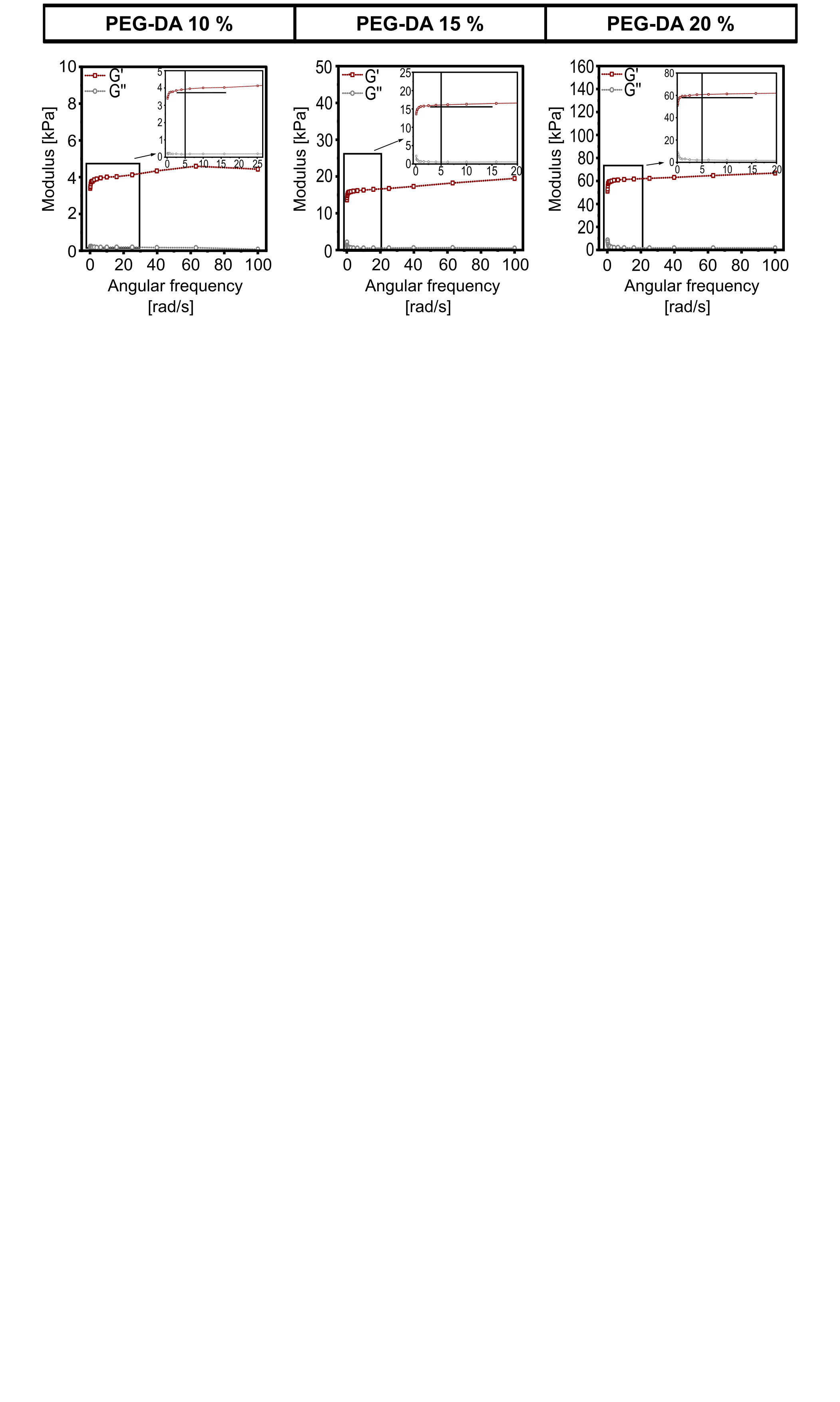


**Figure S10.** Dynamic frequency sweep (DFS) of PEG-PPy hydrogels (PPy 0.25 %). The zoomed in graphs show the linear viscoelastic regime, where the storage modulus is constant.

After determination of the LVR, we carried out dynamic time sweeps at 25 °C or 40 °C with a constant force of 0.3 N, an oscillation strain of 0.1% and an oscillation frequency of 5 rad s^-1^. Each condition was tested with three samples (**Figure S11**).


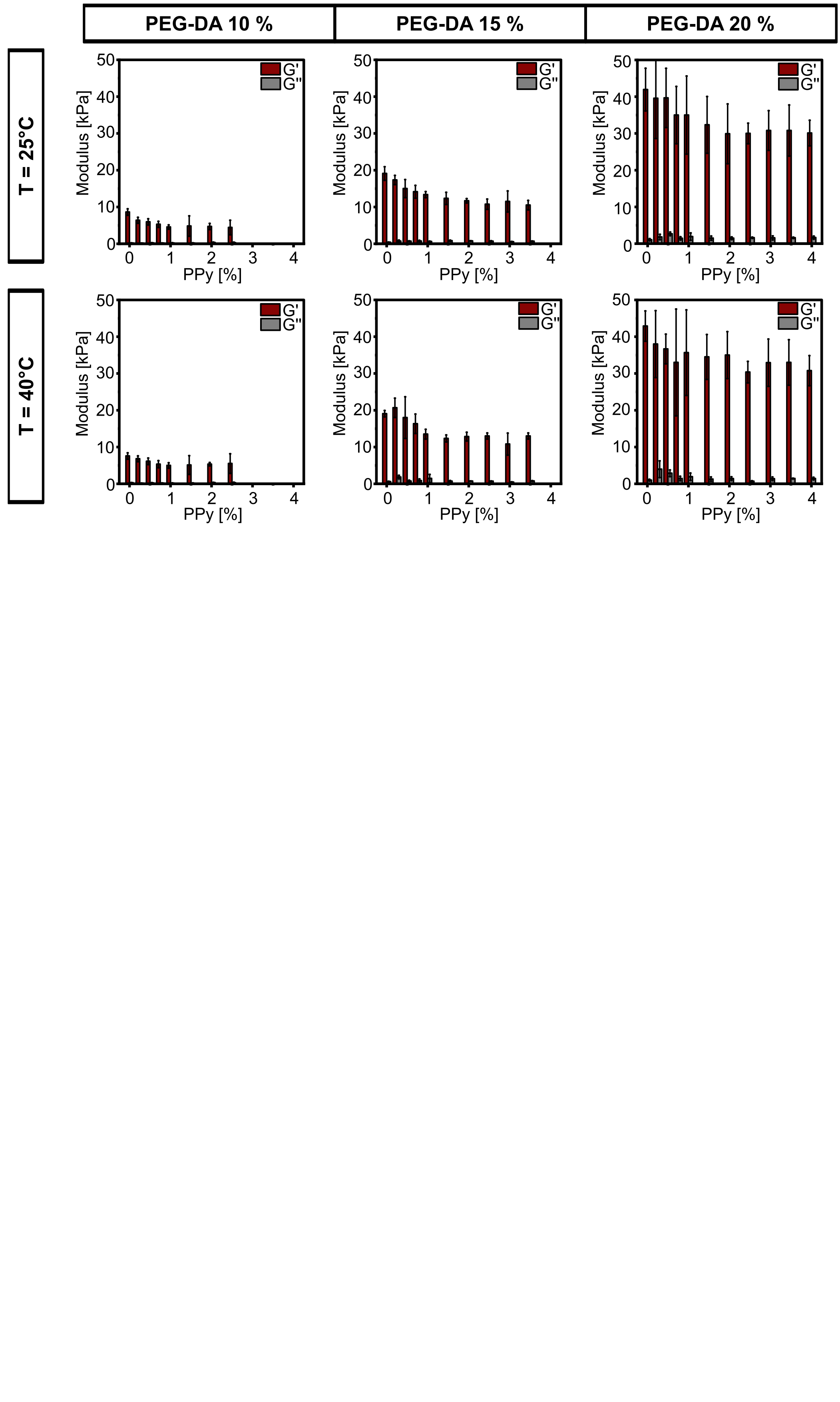


**Figure S11.** Storage modulus (G’) and loss modulus (G’’) of multiple PEG-DA and PPy concentrations measured by dynamic time sweeps (DTS). Number of samples: 3 (*N*= 3). Data is presented as the mean ± the standard deviation.

The degradation studies of the composite gels were carried out by tracking the weight loss (**Figure S12-14**). After synthesis (14 mm diameter hydrogels) the hydrogels were measured in mass ot obtain the mass at day 0 (*m_t=0_*). The gels were submerged in 2 mL solution of either PBS-solution (1X, pH =7.4) or cell culture medium (DMEM (high-glucose) + 10% h.i. FBS + 1% P/S). Afterwards the gels were incubated at 25 °C or 37 °C. Weight assessment of the hydrogels were carried out after 24 h, 48 h, 72 h, 6 d, 7 d, 9 d, 2 weeks. Afterwards the hydrogels were measured weekly for a total of 2 months. For a measurement the hydrogel was transferred out of their solution and residual liquid was removed. The normalized weight (*N*) was determined by following **equation S2**. ^[3]^

(S2)


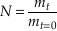

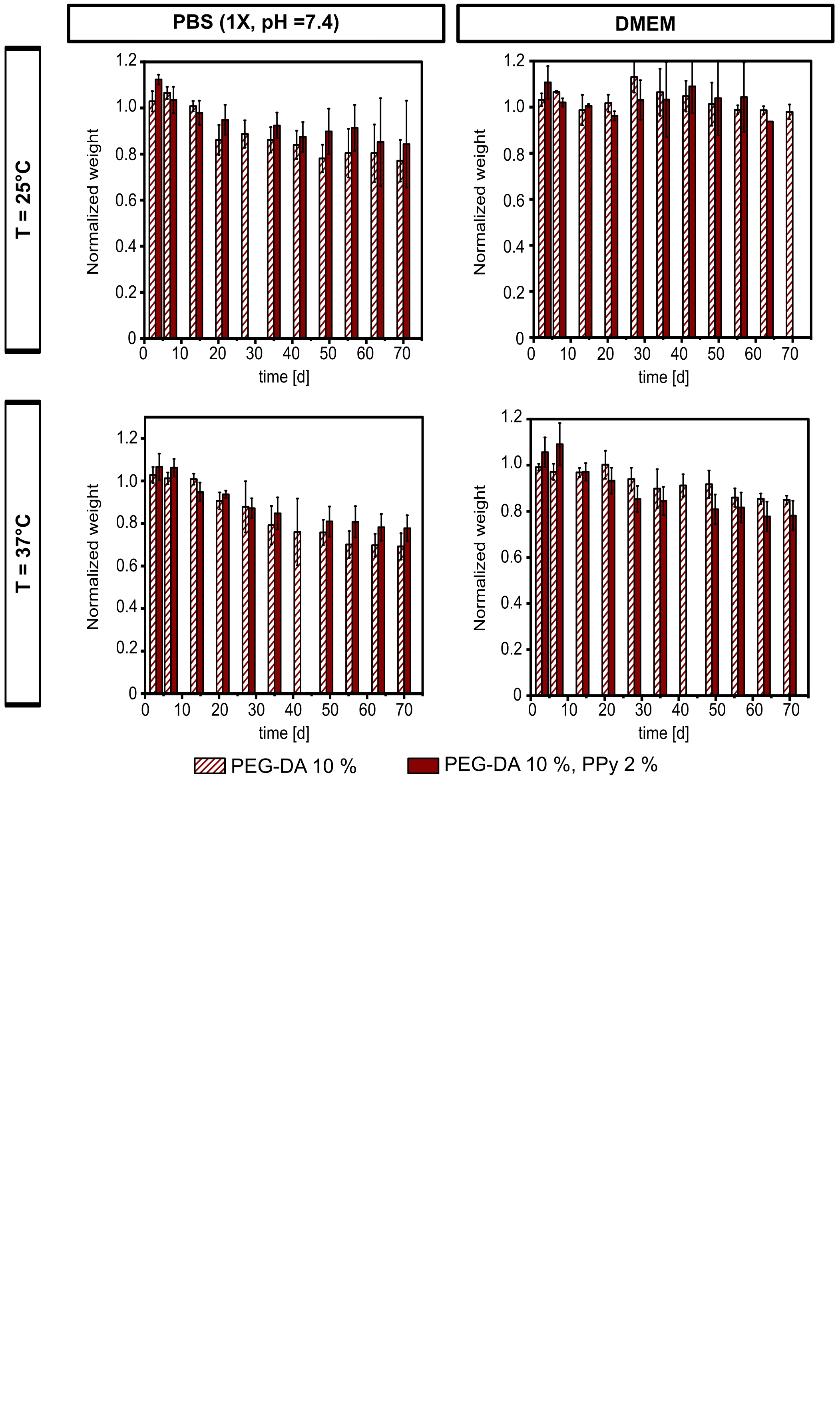


**Figure S12.** Degradation study on PEG-DA 10% hydrogels and PEG-DA 10% + PPy 2% hydrogels in PBS and DMEM at 25°C and 37°C respectively. Number of samples: 3 (*N*= 3). Data is presented as the mean ± the standard deviation.


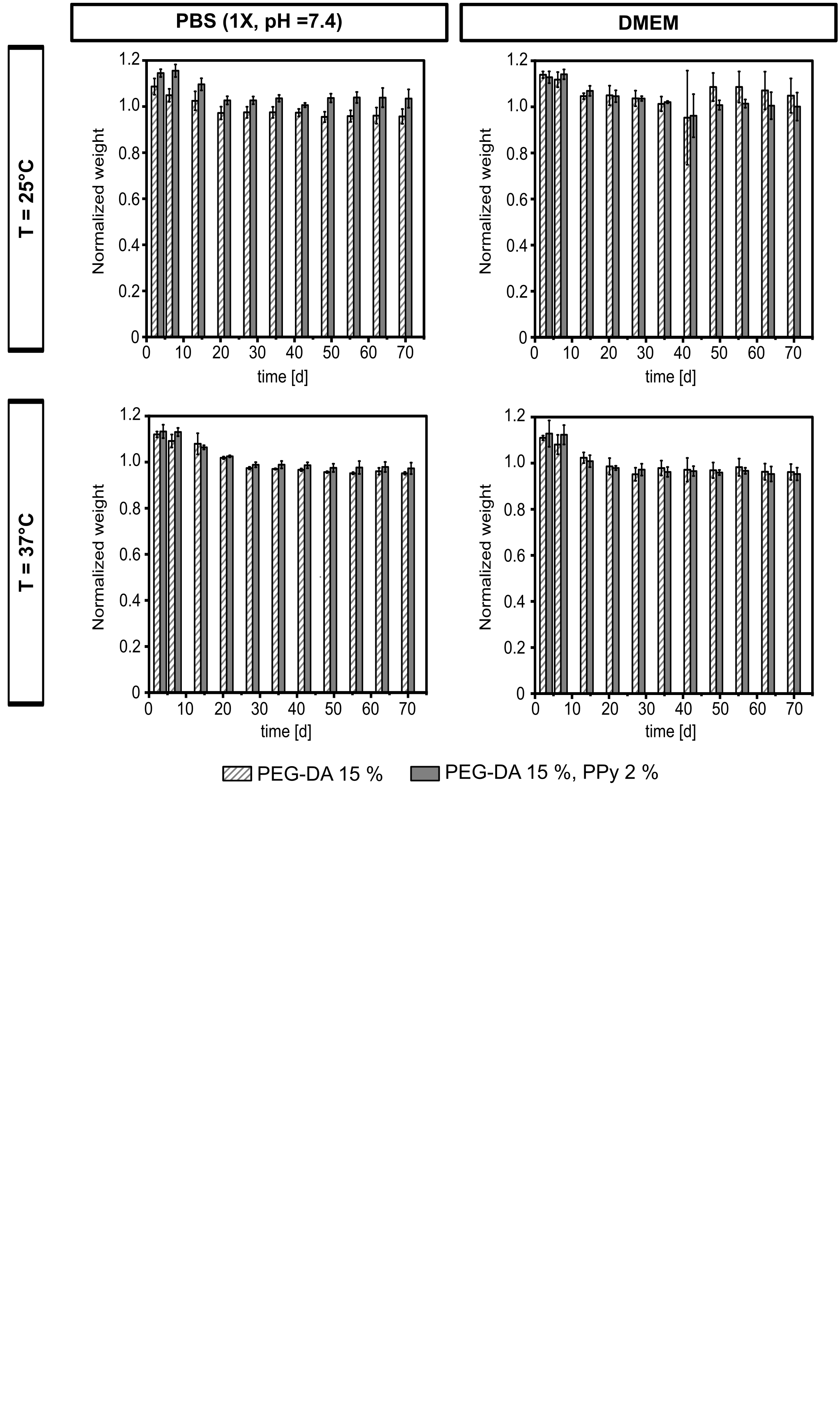


**Figure S13.** Degradation study on PEG-DA 15% hydrogels and PEG-DA 15% + PPy 2% hydrogels in PBS and DMEM at 25 °C and 37 °C respectively. Number of samples: 3 (*N*= 3). Data is presented as the mean ± the standard deviation.


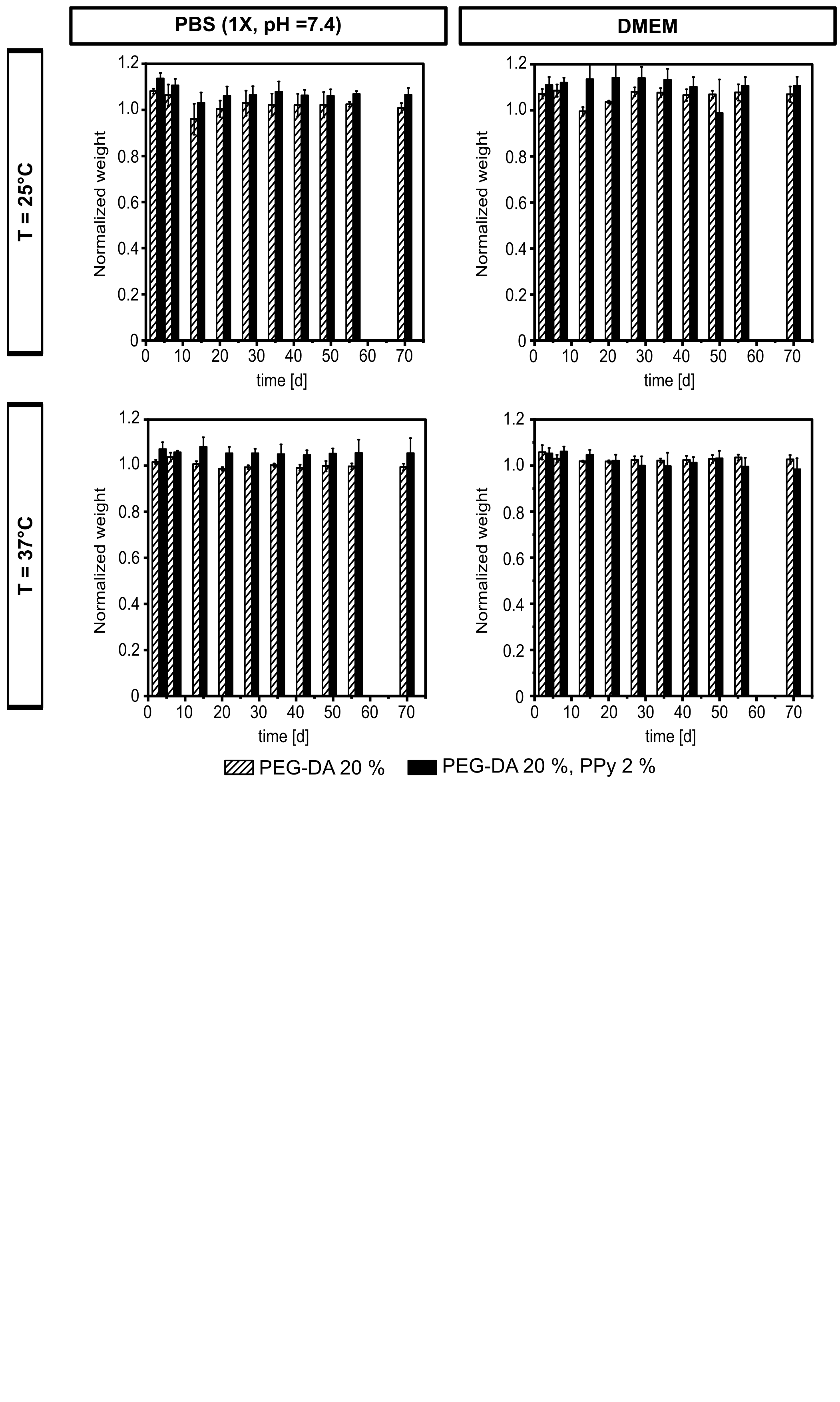


**Figure S14.** Degradation study on PEG-DA 20% hydrogels and PEG-DA 20% + PPy 2% hydrogels in PBS and DMEM at 25 °C and 37 °C respectively. Number of samples: 3 (*N*= 3). Data is presented as the mean ± the standard deviation.

**S6. Electrical and Electrochemical Properties of PEG-PPy Hydrogels**

The composite PEG-PPy hydrogels were further characterized for their electrochemical properties, which are critical for their prospective application in biosensing. The composite hydrogels were placed on a glass slide with gold electrodes attached at both ends. Two probe measurements were conducted, and the conductivity was obtained by relating the resistance (*R*) to the length of the sample ($l$) and the cross-sectional area (*A*) by **equation S3**: ^[4]^

$\sigma=\frac{1}{(R\cdot\frac{l}{A})}$ (S3)

Hydrogels were equilibrated by swelling in deionized water for 48 h at room temperature prior to measurement. Electrical resistance measurements confirmed that the incorporation of PPy as a conductive phase into the hydrogel network enhanced its conductivity. However, after reaching swelling equilibrium, a reduction in conductivity was observed **(Figure S16)**. This decline is attributed to the increased water content within the hydrogel, which expands the polymer network and increases the distance between conductive PPy domains, thereby disrupting the percolation pathways necessary for efficient electron transport.


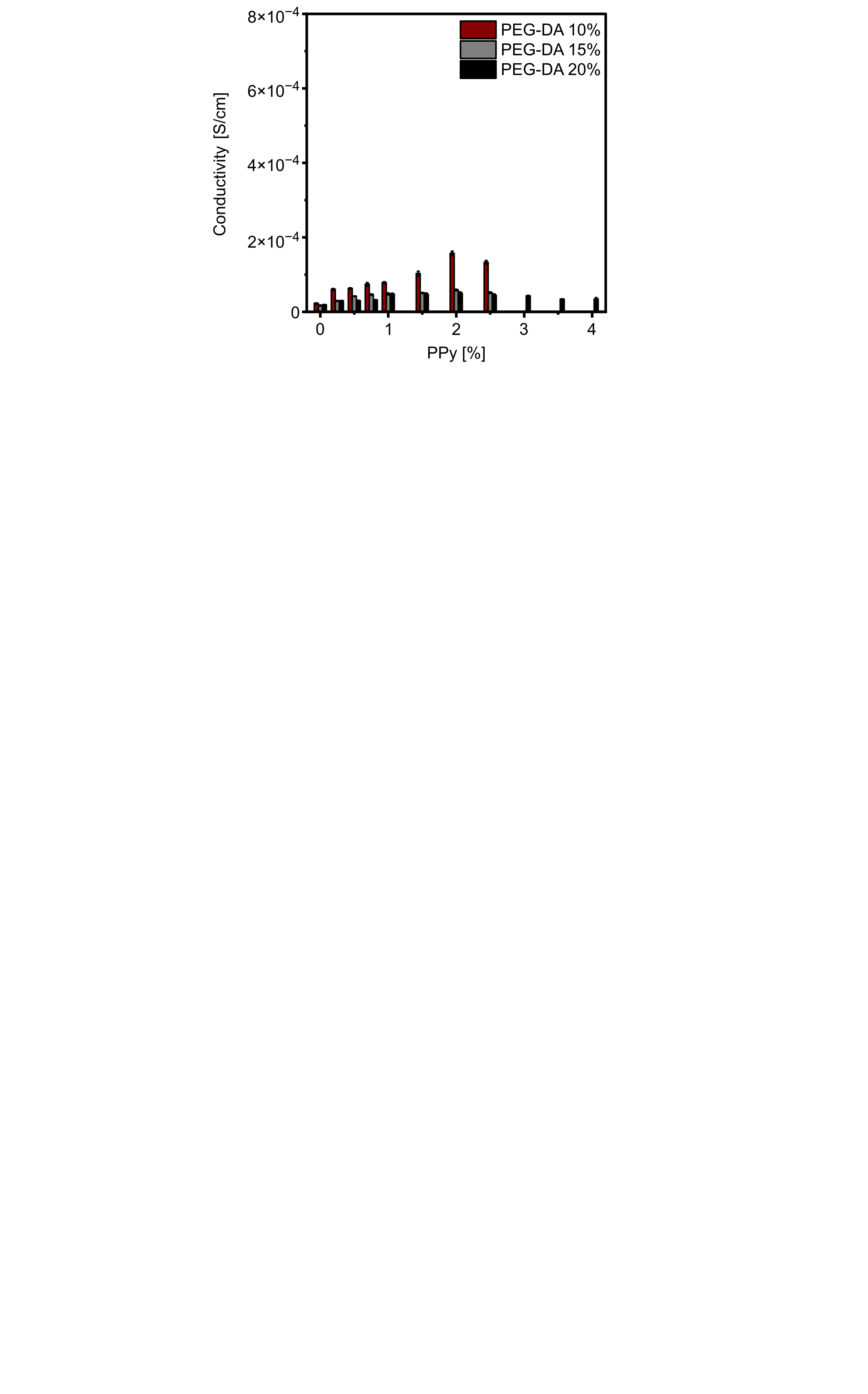


**Figure S15.** Conductivity values of PEG-PPy hydrogels after reaching swelling equilibrium. Hydrogels were swollen in DI-water for 48 h at ambient temperature and measured in the same setup as before. We observe decreased conductivity for all parameters. Number of repetitions: 3 (*N*= 3). Data is presented as the mean ± the standard deviation.

To evaluate the mechanical flexibility and specifically examine the influence of bending on the hydrogel samples electrical properties, three poly(ethyleneterephthalat-glycol) (PETG) angle fixtures with bending angles of 45°, 67.5°, and 90° were fabricated using a *Prusa CoreOne* FDM 3D printer. The hydrogels were positioned on each fixture and electrical characterization was performed by attaching two gold electrodes to the opposing ends of each sample (**Figure S16a-c**). The measured resistance values were normalized by dividing each value by the corresponding resistance obtained at 0°. For bending angles up to 90°, a slight increase in resistance was observed; however, this change was not statistically significant. (**Figure S16d**).


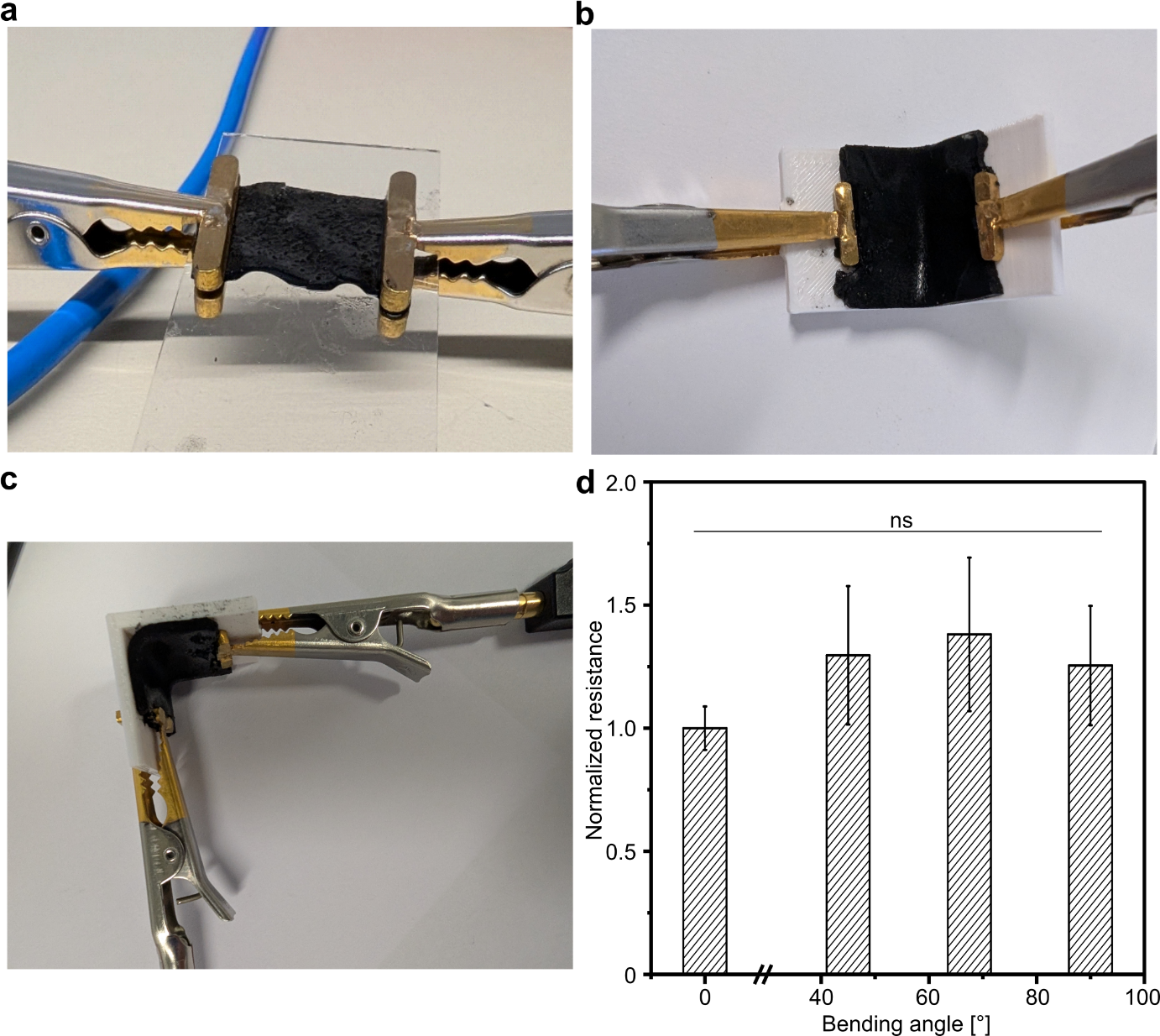


**Figure S16.** Experimental setup of conductivity measurements and current voltage characteristics. **a.** Rectangle composite hydrogel is placed on a glass slide and two gold electrodes are clipped onto each end of the hydrogel. **b.** Electrical measurements at 45° bending. **c.** Electrical measurements at 90°. **d.** Resistance change of PEG-PPy hydrogel during bending. Number of technical replicates: 3 *(N =* 3). Data is presented as the mean ± the standard deviation and analyzed by variance analysis (ANOVA) with ns *p* > 0.05.

The electrochemical properties of the PEG–PPy composite hydrogels were characterized using cyclic voltammetry (CV) and electrochemical impedance spectroscopy (EIS) in a three-electrode setup. A platinum counter electrode, Ag/AgCl reference electrode, and the PEG–PPy hydrogel served as the working electrode. The hydrogel was mounted between two gold-coated glass slides (100 nm Au) and functioned as a self-standing electrode (**Figure S17**). Measurements were conducted in 1× PBS (pH 7.4), with 2 mm of the hydrogel submerged in the electrolyte.


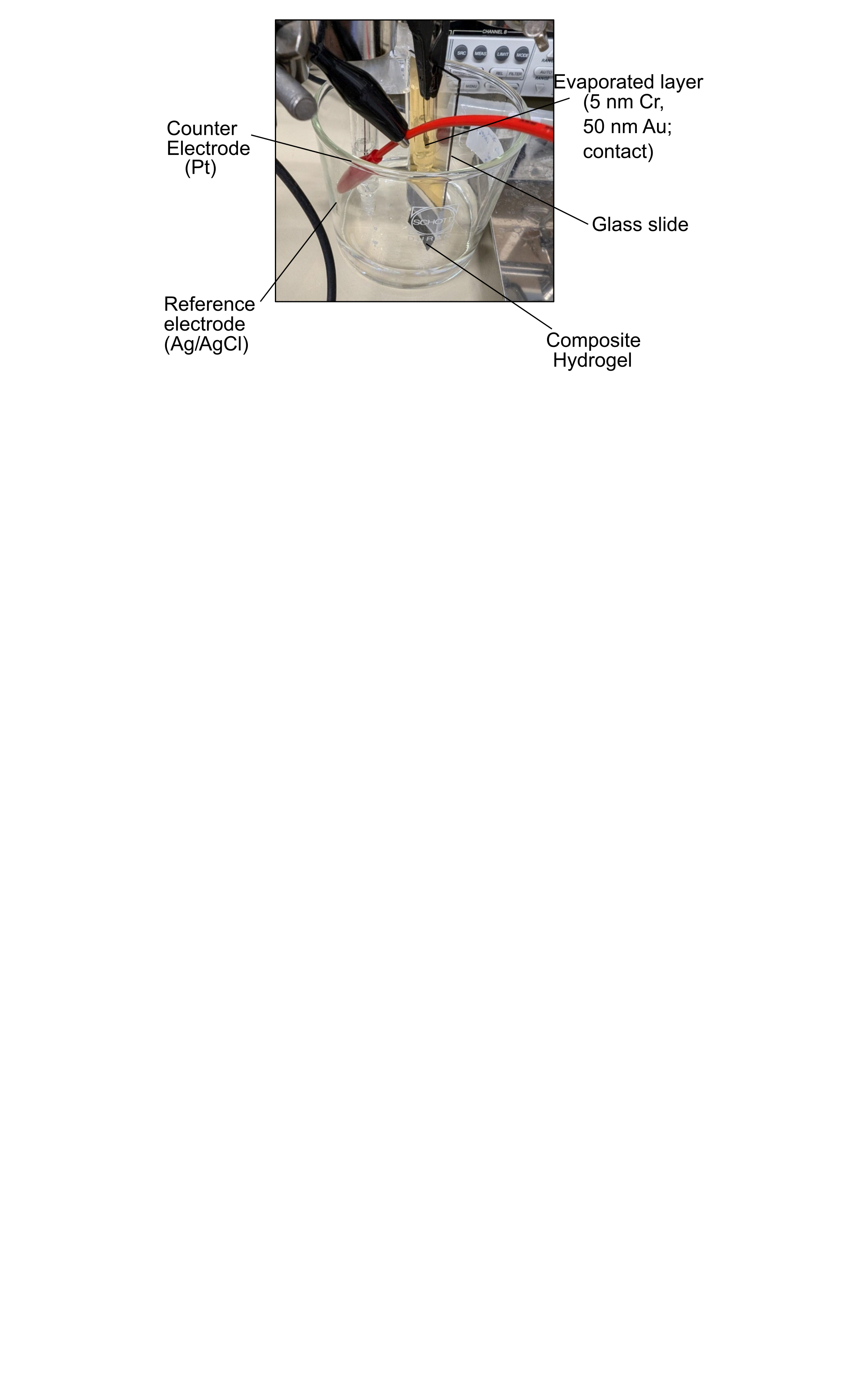


**Figure S17.** Experimental setup for electrochemical measurements (cyclovoltammetry (CV) and electrochemical impedance spectroscopy (EIS)). The composite hydrogel is utilized as working electrode in a three electrode setup. A platinum electrode is used as counter electrode and an Ag/AgCl electrode as reference. PBS is used as electrolyte. The gate electrode was attached to glass slide with 5 nm Cr and 50 nm Au, to ensure good electrical connection.

**S7. PEG-PPy Hydrogel as a Gate Electrode in OECTs**

**Figure S18**. **Output and transfer curves**. Output curves shown in **a**, **b**, **c** and transfer curves with corresponding gate currents (inset) shown in **d**, **e**, and **f**. The PEDOT:PSS channels dimensions were 0.3 mm (width) × 1 mm (length), and PBS was used as the electrolyte.


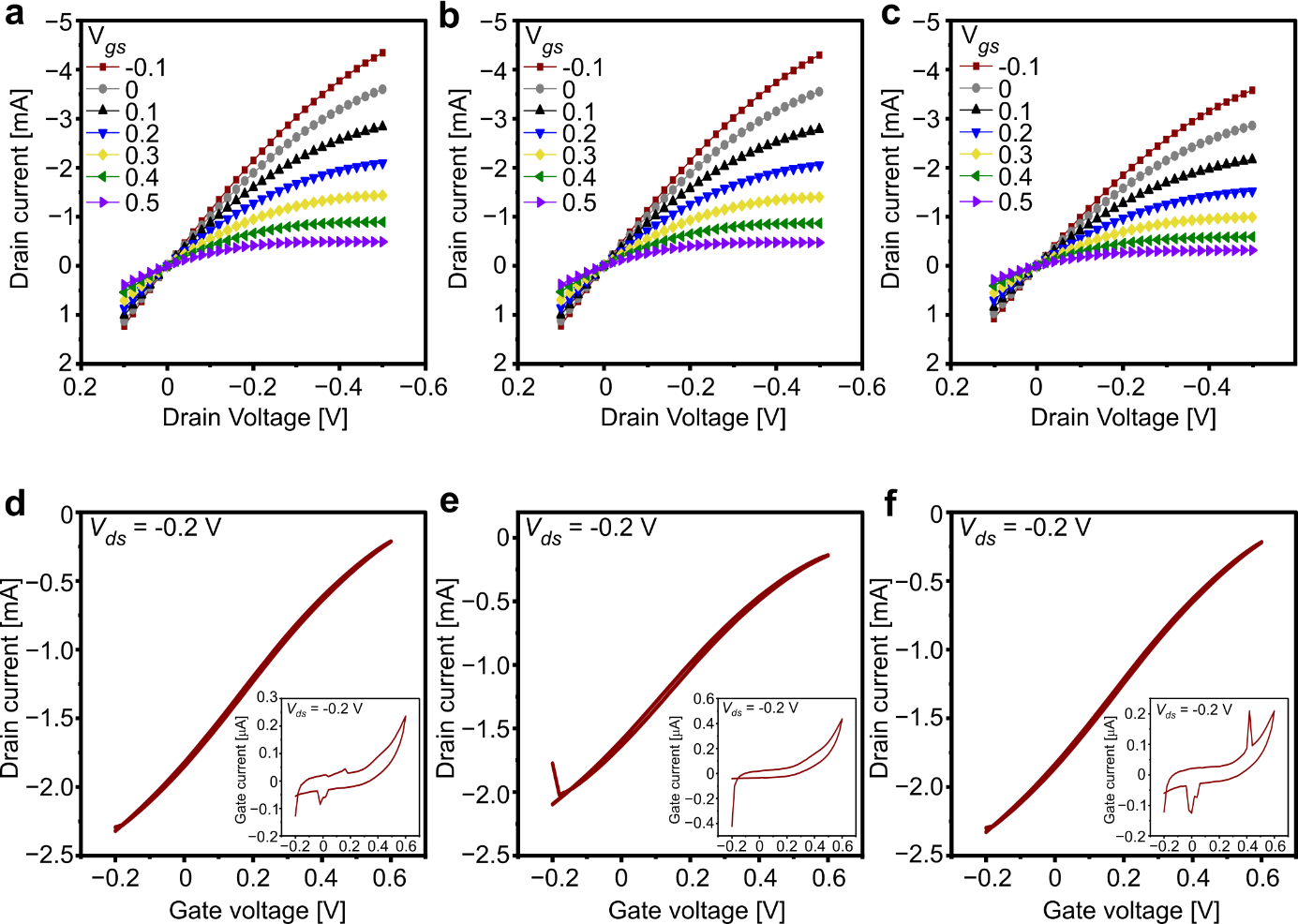


The PEG–PPy hydrogel was evaluated as a gate electrode in OECTs, showing comparable performance to a standard Ag/AgCl pellet. The Ag/AgCl gate achieved peak transconductance of 4.2 mS at 0.2 V, while the PEG–PPy hydrogel reached 3.9 mS at 0.4 V (**Figures S18**).


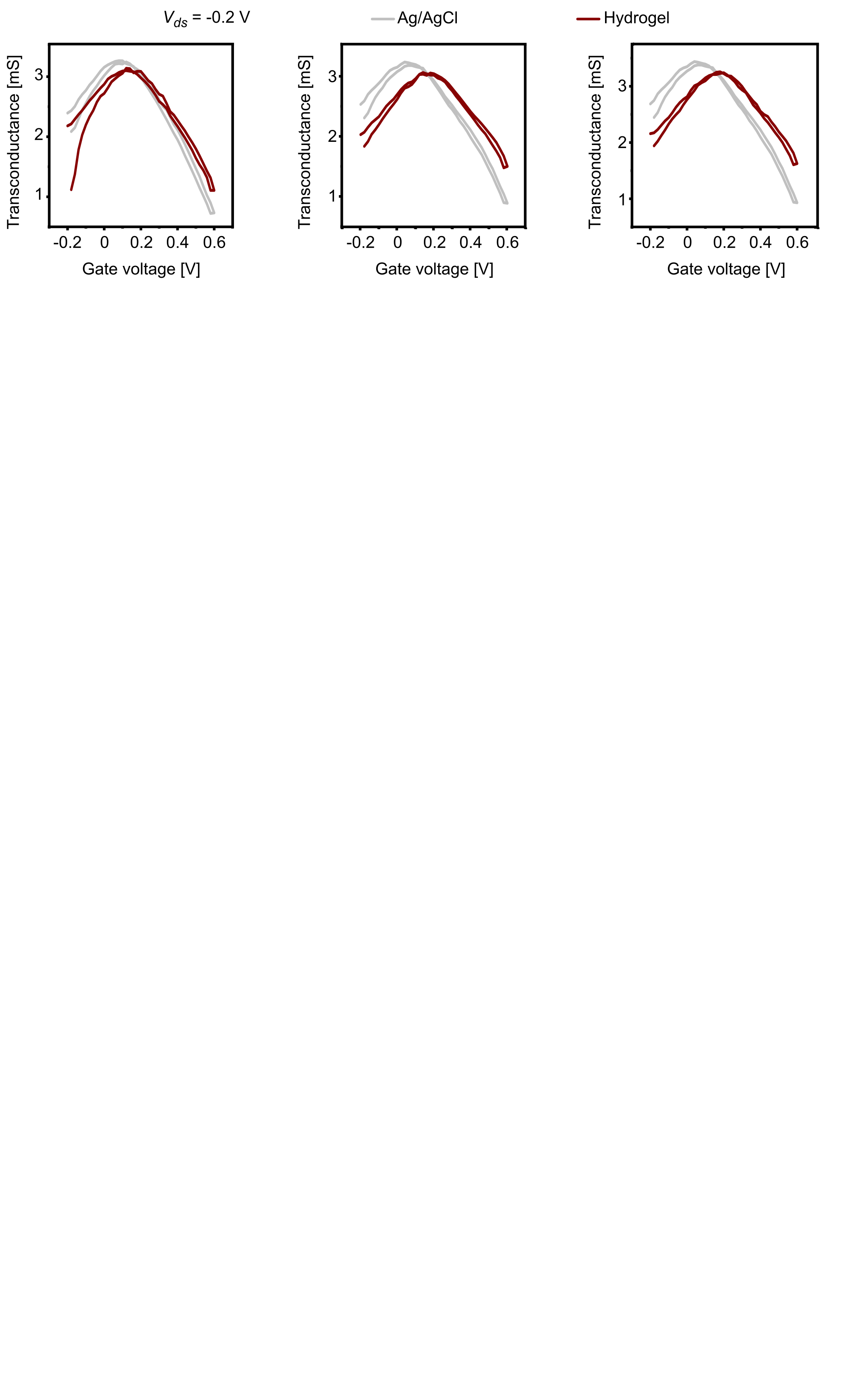


**Figure S19.** Comparison of the transconductance of three OECTs with Ag/AgCl pellet gate electrode and PEG-PPy hydrogel gate electrode.


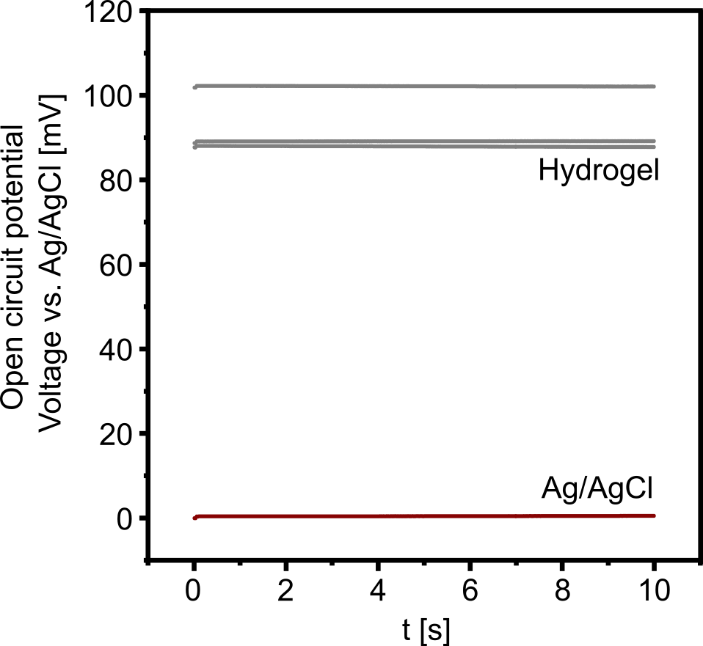


**Figure S20**: Open circuit potential measured in PBS of the gate electrodes in OECT configuration. The source and drain electrodes were short-circuited and used as the counter electrode; the gate electrode (Ag/AgCl pellet or hydrogel) served as the working electrode; and a second Ag/AgCl pellet was used as the reference electrode.


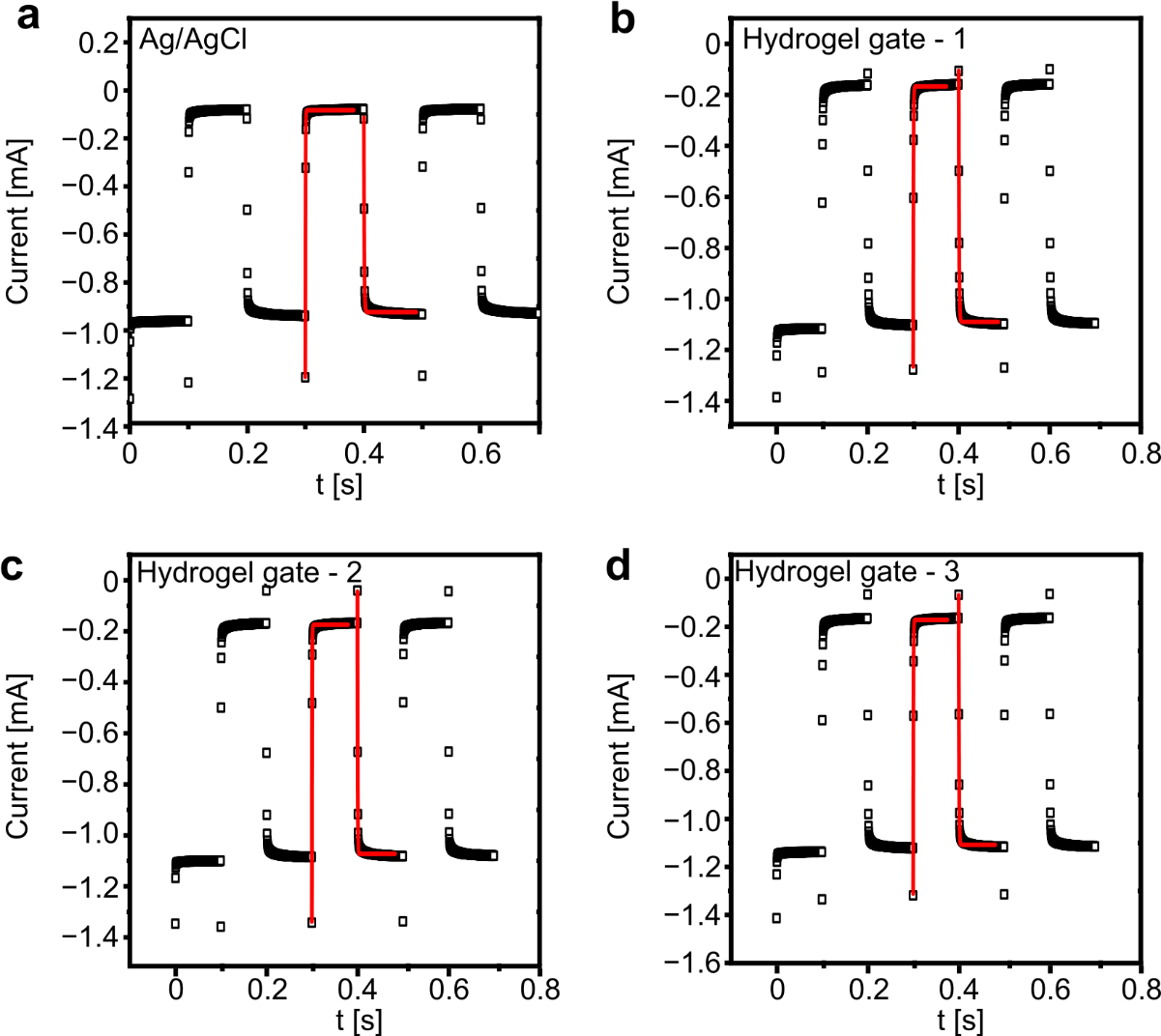


**Figure S21**. **Transient response** of the OECT together with exponential decay fitting curves (red line), from which the characteristic response time (τ_off_ and τ_on_) is obtained. Measurements were performed using **a** an Ag/AgCl gate electrode and **b, c, d** three similar hydrogel gate electrodes. The PEDOT:PSS channels dimensions were 0.3 mm (width) × 1 mm (length), and PBS was used as the electrolyte.


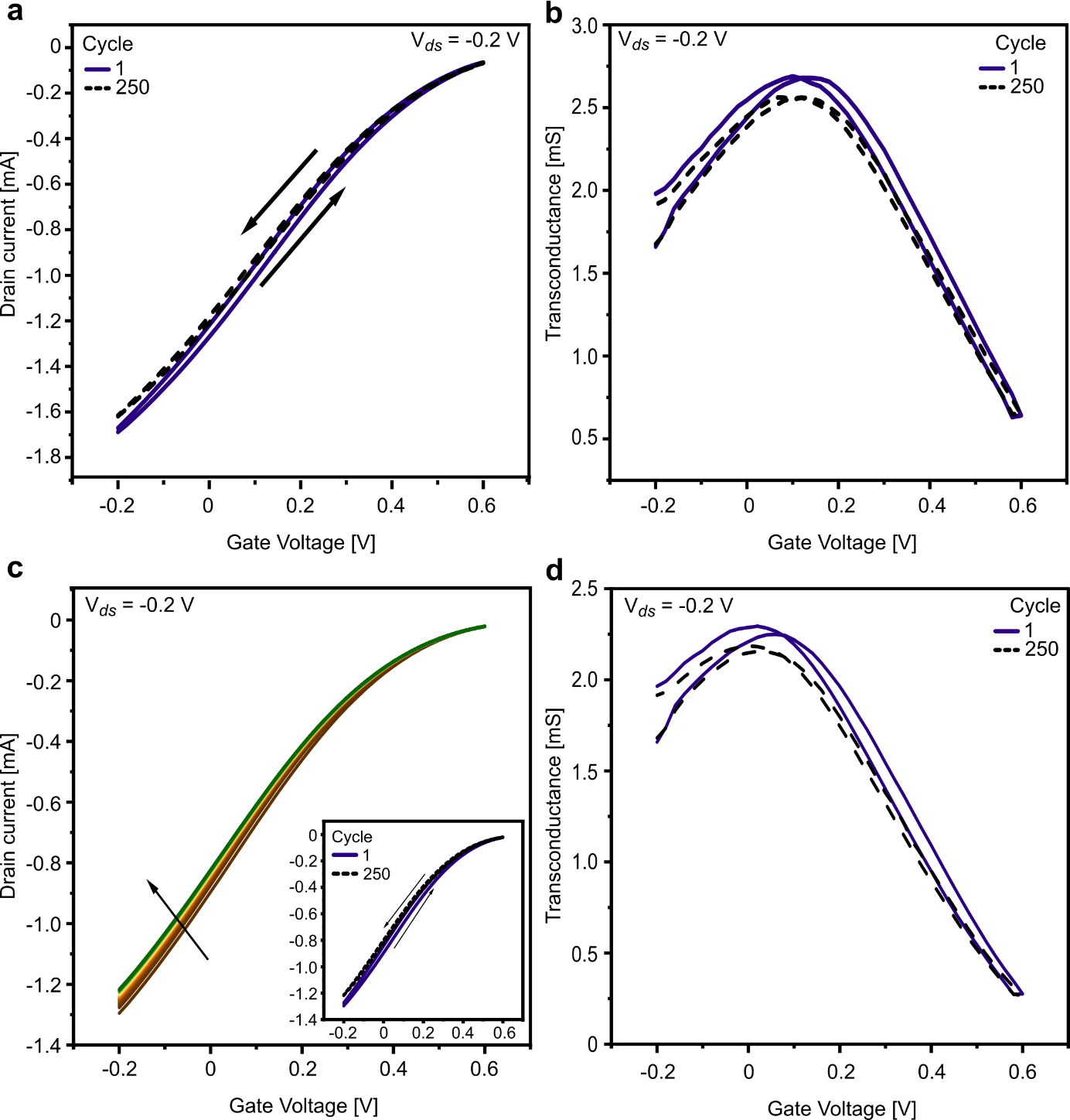


**Figure S22. Stability test of hydrogel-gated OECT**. **a**. Transfer curve over 250 cycles. **b.** Transconductance for cycle 1 and cycle 250. **Stability test of Ag/AgCl-gated OECTs**. **c**. Transfer curve over 250 cycles. **d.** Transconductance for cycle 1 and cycle 250. The PEDOT:PSS channels dimensions were 0.3 mm (width) × 1 mm (length), and PBS was used as the electrolyte.

**Figure S23. Process from 3D-printing to gate electrode.** Fabrication of PEG/PPy hydrogel scaffolds by extrusion-based 3D-printing. The hydrogel scaffold is attached to a gold slide and utilized as gate electrode in an OECT. We used both lattice structure and a honeycomb structure to compare the performance of the scaffolds to mold-casted hydrogels and the Ag/AgCl pellet gate electrode.


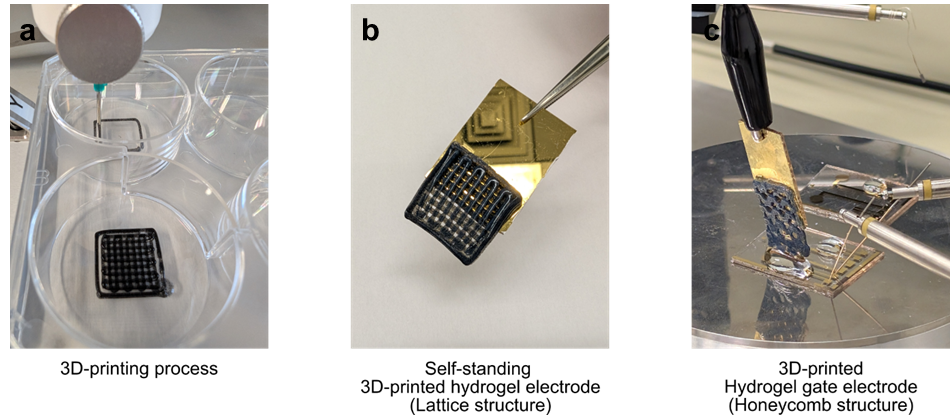


We furthermore compared the performance of PEDOT:PSS coated electrode with the two previous ones (**Figures S24**). The PEDOT:PSS coated electrode achieved similar transconductance values (4 mS). However, at all tested drain-source voltages (-0.2 V, -0.4 V, -0.6 V) the peak transconductance was only achieved at higher voltage (0.6 V).


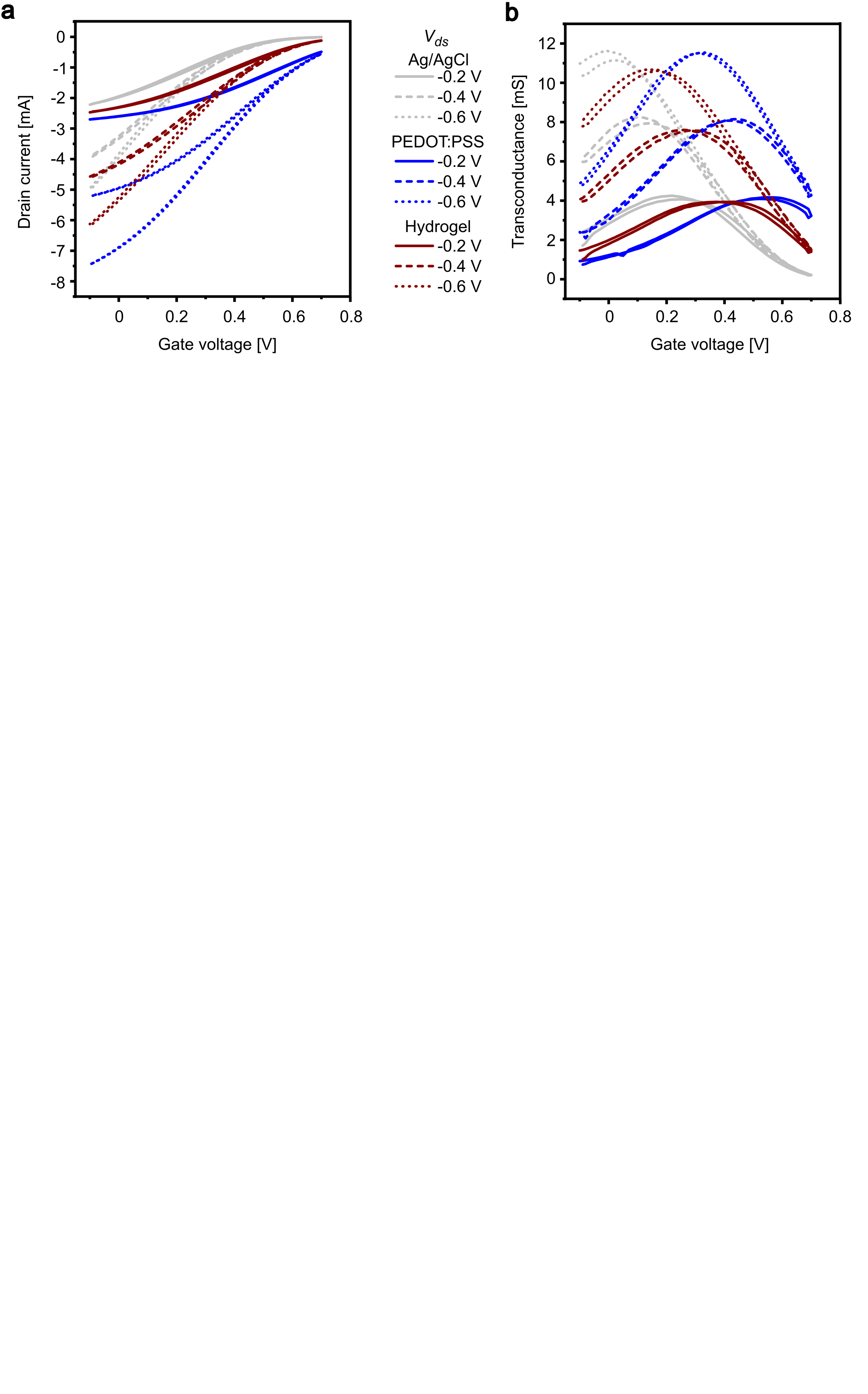


**Figure S24. Comparison of OECTs** with either Ag/AgCl pellet gate electrode, PEG-PPy hydrogel gate electrode, or PEDOT:PSS coated electrode. **a.** OECTs operating in depletion mode at *V_ds_* = -0.2 V, -0.4 V, or -0.6 V. **b.** Transconductance curves at *V_ds_* = -0.2 V, -0.4 V, or -0.6 V. PEDOT:PSS channel dimensions: 0.10 mm (width) x 0.02 mm (length).

**S8. Glucose Oxidase Activity in Enzyme-Loaded PEG-PPy Conductive Hydrogels**

To electrochemically sense glucose we utilized the GOx loaded PEG/PPy-hydrogels as a self-standing electrode in a three electrode setup. A platinum wire was used as the working electrode, an Ag/AgCl pellet electrode as reference and the hydrogel as counter electrode. Upon incremental addition of glucose (every 2 min) we observed an increase in the steady-state current for the chronoamperometry measurements (**Figure S25**).


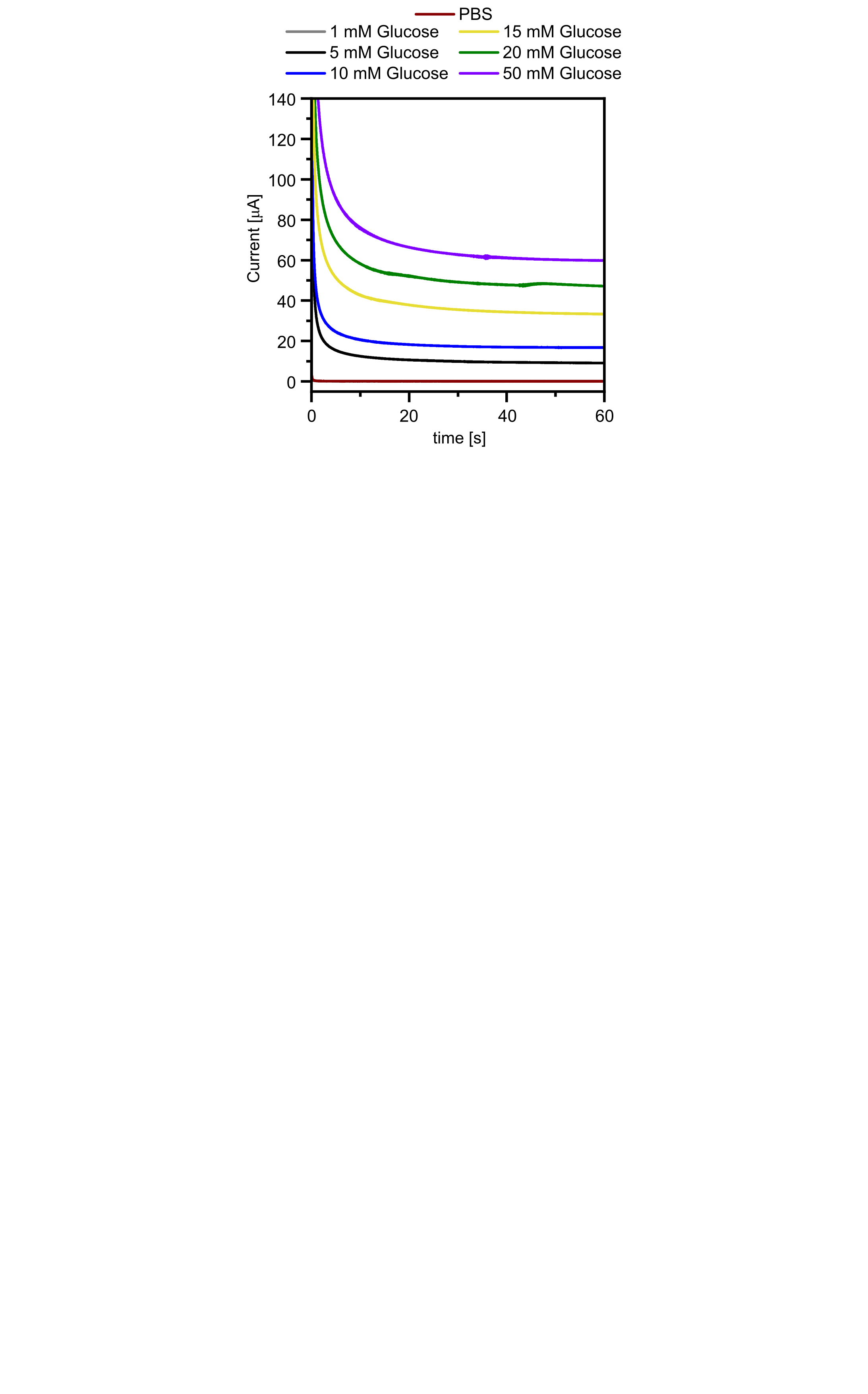


**Figure S25. Chronoamperometric glucose sensing,** with a constant potential of 0.6 V. The same three electrode setup as in **Figure 5d**.

Furthermore, it was tested whether the GOx loaded PEG/PPy hydrogel could be utilized as working electrode. The platinum wire was used as the counter electrode and Ag/AgCl pellet electrode as reference. Upon incremental addition of glucose (every 2 min) we did not observe any oxidation or reduction peaks in cyclic voltammetry (**Figure S26**).


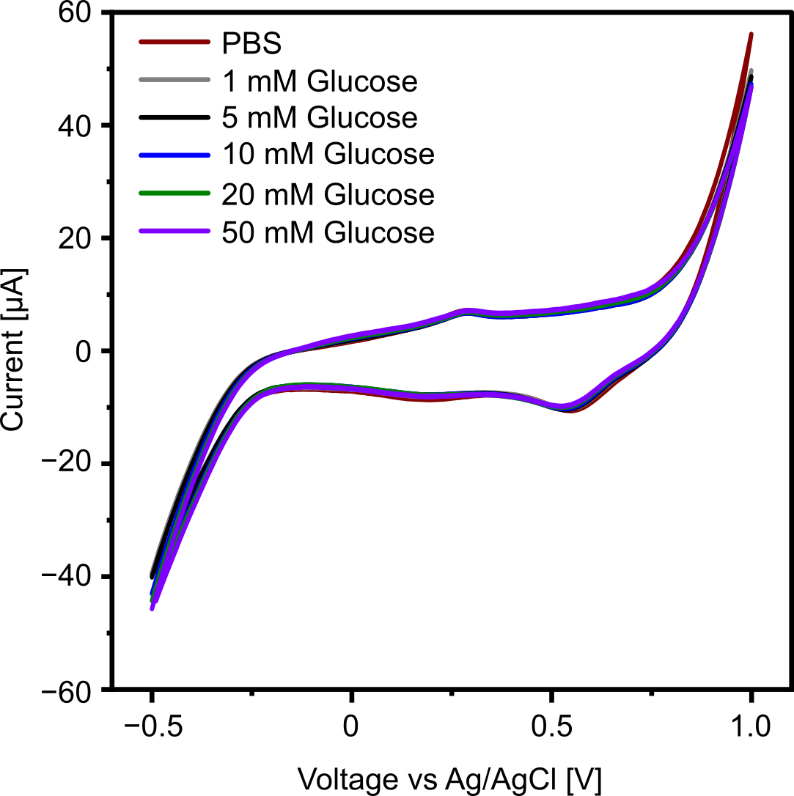


**Figure S26. Cyclic voltammetry measurements with PEG-PPy as working electrode,** potential range of -0.5 V – 1.0 V and a scan rate of 100 mV/s. No glucose sensing can be observed.

For the cyclic voltammetry measurements with GOx loaded PEG/PPy hydrogel as counter electrode and platinum as working electrode, we observed an increasing oxidation peak at 0.6 V as well as an increasing reduction peak at -0.1 V (**Figure S27**).


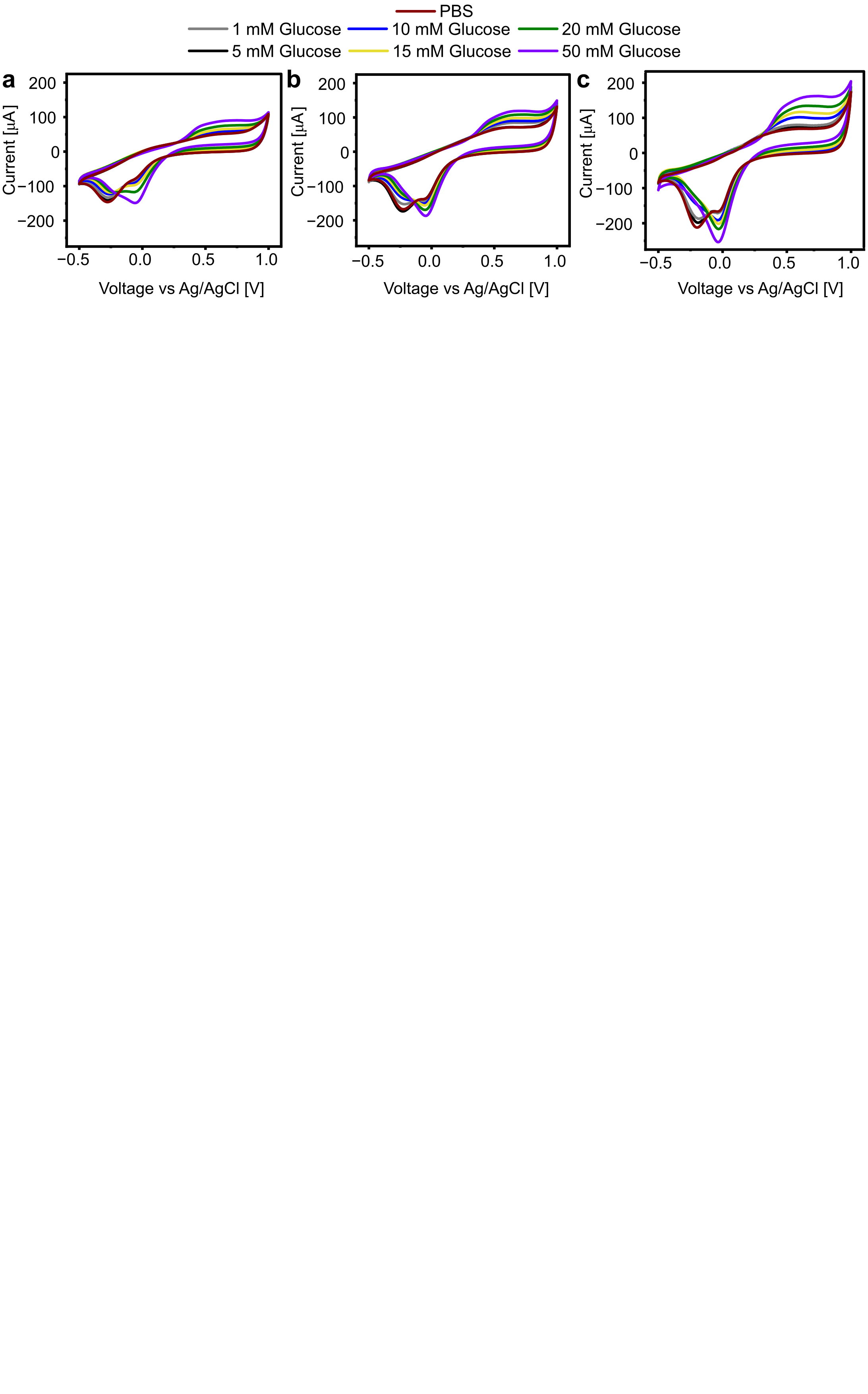


**Figure S27. Cyclic voltametric glucose sensing,** with a potential range of -0.5 V – 1.0 V and a scan rate of 100 mV/s. The same three electrode setup as in **Figure 6d**. For all three independent samples/measurements we observe equal behavior upon incremental glucose addition.

Moreover, we assessed how the PEG/PPy composite material with entrapped GOx (Figure 6e) performs in comparison to a PEG hydrogel with GOx (**Figure S28a-d**), a PEG hydrogel without GOx (**Figure S28e**) and a PEG/PPy hydrogel without GOx (**Figure S28f**) respectively. As described in the experimental section, all measurements were carried out by immersing the working electrode (Pt), the reference electrode (Ag/AgCl) and the counter electrode (hydrogel) in PBS (1X, pH = 7.4). Cyclic voltammetry was carried out in the range of -0.5 V − 1.0 V with a scan rate of 100 mV s^-1^. Glucose solution was added subsequently to obtain a 1 mm, 5 mm, 10 mm, 15 mm, 20 mm and 50 mm glucose solution respectively. Only a small oxidation peak is observed in the measurements even at the highest glucose concentration (50 mm). After normalizing the current we find that the change in current at 20 mm glucose is only 5% in comparison to 66% with the conjugated polymer (**Figure S28d, Figure 6f**). Both hydrogel iterations (PEG and PEG/PPy) without GOx show no oxidation peak independent of the amount of glucose added.


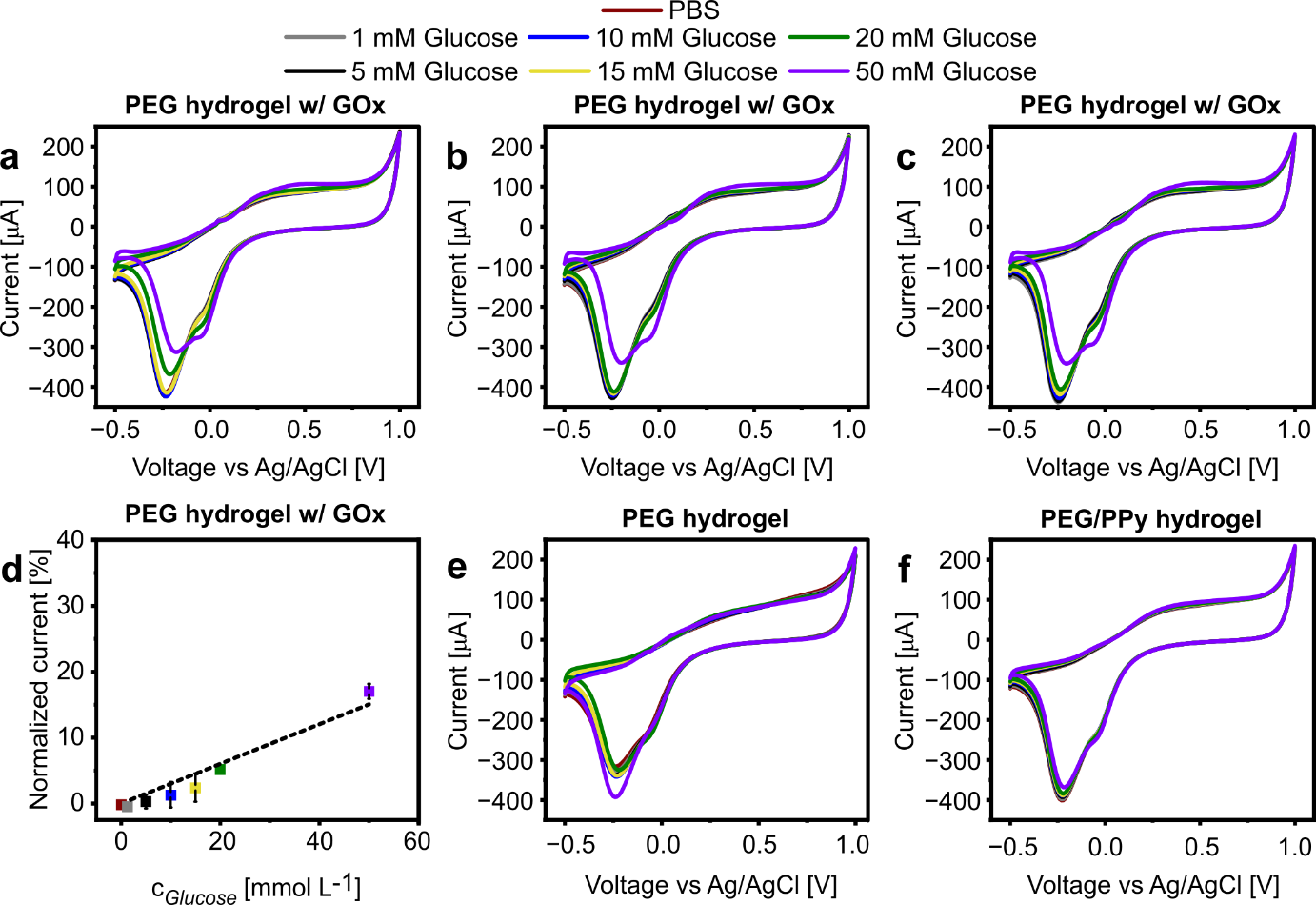


**Figure S28. Comparison of cyclic voltametric glucose sensing,** with a potential range of -0.5 V – 1.0 V and a scan rate of 100 mV/s. The same three electrode setup as in **Figure 6d and Figure S27**. **Figure S28a-c** show three samples of GOx loaded PEG gels without PPy. For all three independent samples/measurements we observe equal behavior (only a small oxidation peak) upon incremental glucose addition. **Figure S28d** displays the analytical curve of the three glucose sensors in **a-c**. The normalized current variation at the oxidation peak is shown as mean ± standard deviation (N = 3 devices). **Figure S28e+f** show the comparison to a PEG and PEG/PPy hydrogel without Gox (no oxidation peak)..


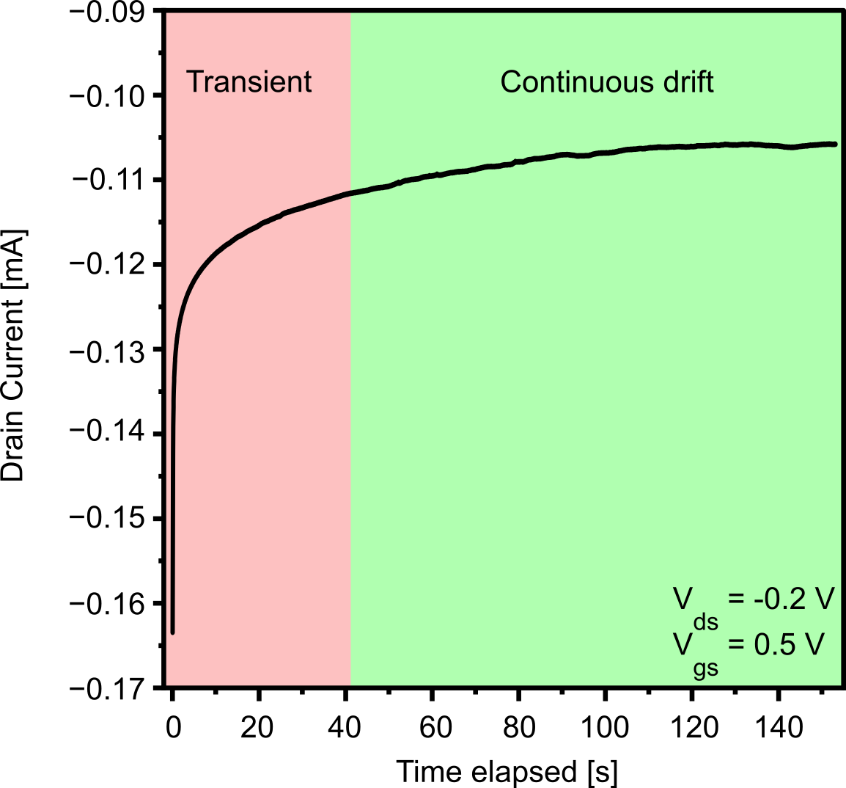


**Figure S29.** Transient response of a hydrogel-gated OECT (V_ds_ = -0.2 V, V_gs_ = 0.5 V).


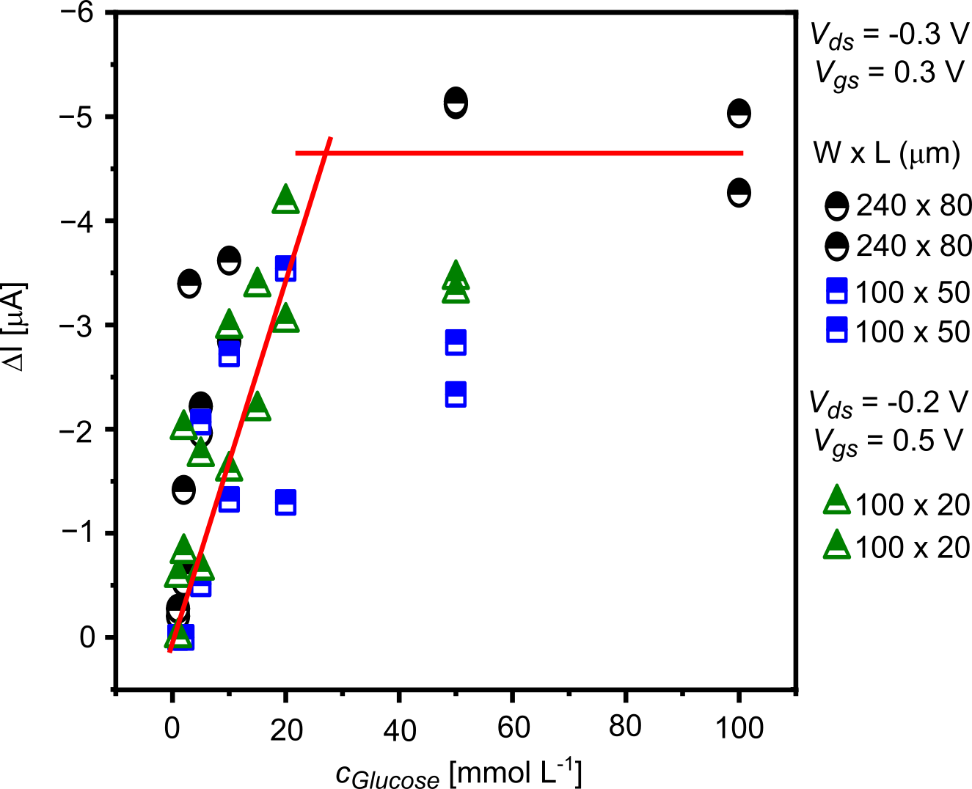


**Figure S30.** Analytical curves of the hydrogel-gated OECT sensors for different channels geometry: 0.240 mm (width) x 0.080 mm (length), 0.100 mm (width) x 0.050 mm (length) and 0.100 mm (width) x 0.020 mm (length).

We further evaluated whether and how much the enzyme is leaking from the hydrogel matrix. To do so we altered the synthesis of the hydrogels by replacing Glucose oxidase (GOx) by CY-5 labeled Glucose oxidase (GOx-CY5, 5mg/mL in PBS). The standard synthesis procedure was utilized and the gels were submerged in 3 mL PBS solution (1X, pH = 7.4). After 1 d, 2 d, 3 d, 7 d, 10 d, and 14 d aliquots from the supernatant were analyzed in a plate reader with equal amounts of PBS as blank and a GOx-CY5 solution as reference. For plate reader analytics the solutions were linearly shaken for 5 s with 1 mm amplitude and 1440 rpm frequency. A wavelength of *λ* = 600 nm (10 nm bandwidth, monochromator) was used for excitation and emission was measured at *λ* = 670 nm (10 nm bandwidth, monochromator). The measured fluorescence values were first baseline-corrected by subtracting the corresponding blank signal. Subsequently, the baseline-corrected data were normalized by dividing each value by that of the GOx-CY5 solution to obtain the percentage of GOx that leaked from the hydrogel into the supernatant.

We found that GOx slowly leaks from the hydrogel over longer time periods in a near linear manner. After 14 days approximately 13.5 ± 3.4% GOx have leached from the hydrogel (**Figure S31**).


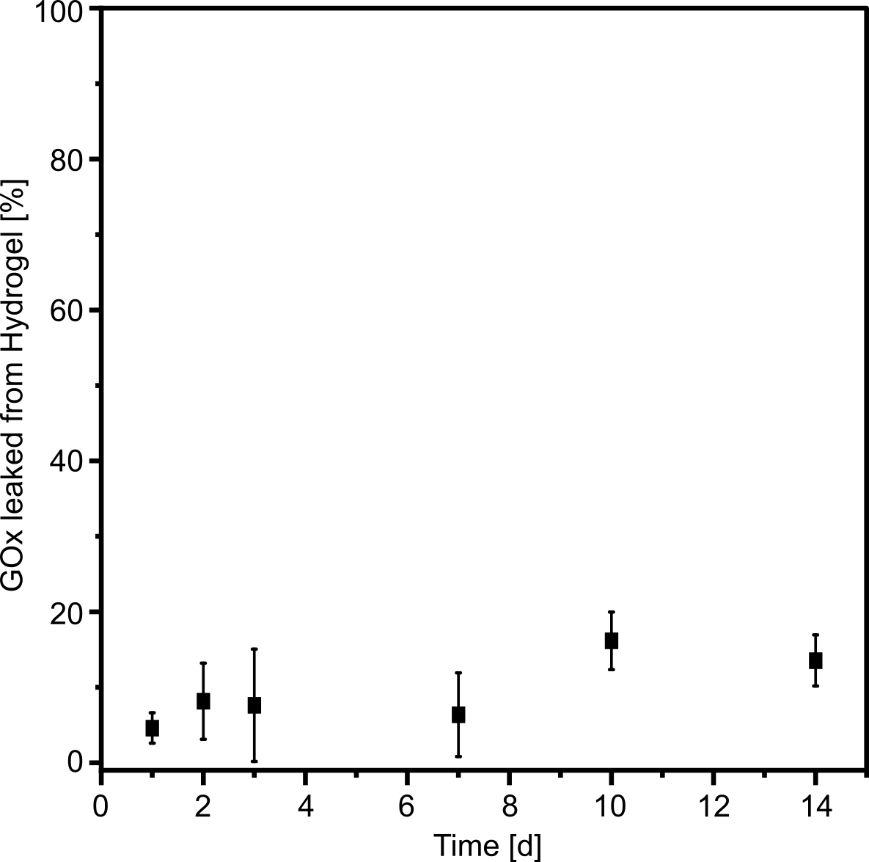


**Figure S31**: CY5 labeled Glucose oxidase (GOx-CY5) leakage from PEG-PPy hydrogels over 14 d. Number of samples: 3 (*N*= 3). Data is presented as the mean ± the standard deviation.

**Table S1.** Comparison of reported soft bioelectronic materials for OECTs.

| Material | Conductivity | Elastic modulus | Printability | Trans-conductance | Reference |
| --- | --- | --- | --- | --- | --- |
| PAAc/p(g2T-T) | Not reported | ~80-900 kPa | Not reported | 8.54 mS | Dai et al.^[5]^ |
| pristine PEDOT:PSS | 40 S cm^-1^ | ~2 MPa | Patterning/printable | Not reported | Lu et al.^[6]^ |
| PVA/PAA borax | 0.09 S cm^-1^ | 79-96 kPa | Not reported | Not reported | Sun et al.^[7]^ |
| Gelatine | Not reported | Not reported | Not reported | 0.05 - 0.27 mS | Jo et al.^[8]^ |
| PNIPAM/  PEDOT:PSS | Not reported | 190-460 kPa | DLP printable | 11.4 - 13.1 mS | Lopez-Larrea et al.^[9]^ |
| PEDOT-PAM-PDA | 0.04 S cm^-1^ | 4 kPa | Not reported | Not reported | Lao et al.^[10]^ |
| PEDOT:PSS / poly(AAm-*ran*-AMPS) | 50-80 S cm-1 | Not reported | Not reported | ∼40 mS | Alex C. Tseng et al.^[11]^ |
| Poly(Cu-NIPAm) | 144 S cm^-1^ | 70-160 kPa | Not reported | Not reported | Xia et al.^[12]^ |
| PAA/PEDOT:PSS | 0.09 S cm^-1^ | 650 kPa | 3D  printable | Not reported | Wang et al.^[13]^ |
| PEDOT:PSS | 0.1 S cm^-1^ | 1 kPa | extrudable/injectable | 1.4 mS | Zhang et al.^[14]^ |
| PEDOT:PSS/ PAAMPSA/IL | 320 S cm^−1^ | 800 kPa | spray-coating | 12.95 mS | Su et al.^[15]^ |
| PEI/PEDOT:PSS | 400 S cm^−1^ | 1.62 GPa | extrudable/injectable | 19 mS | Jo et al.^[16]^ |
| GelMA/  PEDOT:PSS | 1.1•10^-3^ S cm^-1^ | 200-300 kPa | extrudable/injectable | 0.1 mS | Chou et al.^[17]^ |
| PEG-PPy | 7∙10^-4^ ± 7∙10^-5^ S cm^-1^ | 15 – 100 kPa | 3D printable | 3.2 ± 0.1 mS | This work |

**S9. References**

[1] M. Seike, M. Uda, T. Suzuki, H. Minami, S. Higashimoto, T. Hirai, Y. Nakamura, S. Fujii, *ACS omega* **2022**, *7*, 13010-13021.

[2] A. Nimpaiboon, S. Amnuaypornsri, J. Sakdapipanich, *Polymer Testing* **2013**, *32*, 1135 1144.

[3] S. Scott, M. Villiou, F. Colombo, A. D. la Cruz – Garcia, L. Tydecks, L. Toelke, K. Siemsen, C. Selhuber – Unkel, *Advanced Materials* **2025,** 2408616.

[4] D. Halliday, R. Resnick, J. Walker, *Physik*, John Wiley & Sons, **2013**.

[5] Y. Dai, S. Wai, P. Li, N. Shan, Z. Cao, Y. Li, Y. Wang, Y. Liu, W. Liu, K. Tang, *Science* **2024**, *386*, 431-439.

[6] B. Lu, H. Yuk, S. Lin, N. Jian, K. Qu, J. Xu, X. Zhao, *Nature communications* **2019**, *10*, 1043.

[7] H. Sun, S. Wang, F. Yang, M. Tan, L. Bai, P. Wang, Y. Feng, W. Liu, R. Wang, X. He, *Materials Horizons* **2023**, *10*, 5805-5821.

[8] Y. J. Jo, K. Y. Kwon, Z. U. Khan, X. Crispin, T.-i. Kim, *ACS applied materials & interfaces* **2018**, *10*, 39083-39090.

[9] N. Lopez‐Larrea, S. Wustoni, M. I. Peñas, J. Uribe, A. Dominguez‐Alfaro, A. Gallastegui, S. Inal, D. Mecerreyes, *Advanced Functional Materials* **2024**, *34*, 2403708.

[10] J. Lao, Y. Jiao, Y. Zhang, H. Xu, Y. Wang, Y. Ma, X. Feng, J. Yu, *ACS nano* **2025**, *19*, 7755-7766.

[11] A. C. Tseng, T. Sakata, *ACS Applied Materials & Interfaces* **2022**, *14*, 24729-24740.

[12] X. Xia, Q. Liang, X. Sun, D. Yu, X. Huang, S. M. Mugo, W. Chen, D. Wang, Q. Zhang, *Advanced Functional Materials* **2022**, *32*, 2208024.

[13] F. Wang, Y. Xue, X. Chen, P. Zhang, L. Shan, Q. Duan, J. Xing, Y. Lan, B. Lu, J. Liu, *Advanced Functional Materials* **2024**, *34*, 2314471.

[14] S. Zhang, Y. Chen, H. Liu, Z. Wang, H. Ling, C. Wang, J. Ni, B. Çelebi‐Saltik, X. Wang, X. Meng, *Advanced Materials* **2020**, *32*, 1904752.

[15] X. Su, X. Wu, S. Chen, A. M. Nedumaran, M. Stephen, K. Hou, B. Czarny, W. L. Leong, *Advanced Materials* **2022**, *34*, 2200682.

[16] Y. J. Jo, S. Y. Kim, J. H. Hyun, B. Park, S. Choy, G. R. Koirala, T.-i. Kim, *npj Flexible Electronics* **2022**, *6*, 31.

[17] C.-C. Chou, Y.-S. Hsiao, M.-F. Hou, P.-Y. Zhang, M.-Y. Hsieh, L.-Y. Weng, *Chemical Engineering Journal* **2025**, 168573.
